# Global burden and trends of major mental disorders in individuals under 24 years of age from 1990 to 2021, with projections to 2050: insights from the Global Burden of Disease Study 2021

**DOI:** 10.3389/fpubh.2025.1635801

**Published:** 2025-09-16

**Authors:** Wei Liu, Yu Zhang, Jie Chen, Xiayang Li, Yishuai Huang, Fuyang Zhao, Fangyao Chen, Pengfei Qu, Yajun Li

**Affiliations:** ^1^Northwest Women’s and Children’s Hospital, Xi'an, China; ^2^Department of Epidemiology and Biostatistics, School of Public Health, Xi'an Jiaotong University Health Science Center, Xi'an, China; ^3^School of Nursing, Shaanxi University of Chinese Medicine, Xianyang, China; ^4^Translational Medicine Center, Northwest Women’s and Children’s Hospital, Xi'an, China; ^5^Central Laboratory, Beijing Obstetrics and Gynecology Hospital, Capital Medical University, Beijing, China

**Keywords:** mental disorders, disability-adjusted life-years (DALYs), health inequality, global burden, projections to 2050

## Abstract

**Background:**

Mental disorders are the leading cause of global non-fatal disease burden, with rising prevalence among children, adolescents, and youth. This study analyzed location-, age-, and sex-specific estimates for nine mental disorders from 1990 to 2021 using GBD 2021 data, projecting burden to 2050.

**Methods:**

Methods included calculating average annual percentage change (AAPC) and annual percentage change (APC) for age-standardized prevalence rate (ASPR) and disability-adjusted life years rate (ASDR), alongside decomposition, inequality, frontier, comparative risk, and Bayesian age-period-cohort analyses.

**Results:**

Compared to 1990, the 2021 global burden significantly increased among youth [the AAPC of ASPR = 0.15, 95% confidence interval (CI): 0.14 to 0.16; the AAPC of ASDR = 0.40, 95% CI: 0.29 to 0.51], accelerating sharply after 2019 (the APC of ASPR = 4.74, 95% CI 4.55 to 4.93; the APC of ASDR = 6.64, 95% CI 4.88 to 8.42). Males experienced a higher burden than females, with variations in sex-specific patterns across age groups. Burden varied substantially by socio-demographic index (SDI), being highest in high-SDI regions [ASPR = 12913.13, 95% uncertainty interval (UI): 11135.82 to 14874.98; ASDR = 1750.41, 95% UI: 1253.46 to 2328.87]. We found that the burden changes of nine mental disorders vary at the global, regional, and national levels. Decomposition analysis highlighted that the changes in prevalence and the disability-adjusted life years (DALYs) were predominantly driven by population growth (84.86% and 57.92%), with the most significant improvements observed in higher SDI regions. Frontier analysis revealed the potential for burden reduction in higher income countries and territories. Globally, key risk factors included childhood sexual abuse, bullying, intimate partner violence, and lead exposure were identified for anxiety disorders, depressive disorders, and idiopathic developmental intellectual disability, respectively. Projections indicated that the burden of mental disorders is likely to continue its decline in 2050 (ASPR = 6120.71 per 100,000, 95% CI: 3973.57 to 8267.85; ASDR = 844.71 per 100,000, 95% CI: 529.48 to 1159.94).

**Conclusion:**

Despite projected rate declines by 2050, the global burden of mental disorders is increasing, with significant disparities across populations and a recent surge demanding intensified prevention and equitable healthcare expansion worldwide.

## 1 Introduction

Over the past decade, mental disorders have been increasingly recognized as a major contributor to the global disease burden, exhibiting particularly high prevalence among younger populations, with rates peaking around age 14 (1, 2). According to the World Health Organization (WHO) estimates, nearly 970 million people worldwide lived with a mental disorder in 2019. Globally, mental disorders accounted for 5.1% of the disease burden and are the leading cause of years lived with disability (YLD), responsible for one in every six (15.6%) YLDs. Critically, more than 80% of individuals with mental disorders reside in low- and middle-income countries (LMICs). LMICs often rooted in profound socio-economic challenges, including severe shortages of mental health professionals, fragmented service delivery systems, pervasive stigma, high poverty rates, and in some cases, conflict or displacement, creating formidable barriers to accessing care. In these settings, a vicious cycle links mental health challenges and poverty, exacerbated by inadequate welfare safety nets and limited access to effective treatment. Beyond the direct costs of treatment, these conditions incur substantial indirect costs through reduced economic productivity, higher unemployment rates, and other socioeconomic impacts. Researchers from the World Economic Forum calculated that a broadly defined set of mental health conditions cost the global economy approximately US$ 2.5 trillion in 2010, with projections soaring to US$ 6 trillion by 2030 due to rising social costs. LMICs are expected to bear 35% of this escalating economic burden. This projected figure exceeds the combined projected costs of cancer, diabetes, and chronic respiratory diseases.

Population aged under 24 years (including children, adolescents, and youth) represents pivotal developmental phases for mental health, characterized by rapid brain development (3). Early onset of mental disorders can obstruct the transition to a healthy adulthood and restrict opportunities for a productive life thereafter (4, 5). Therefore, understanding the burden of mental disorders in early life is crucial, as it can help shape public policies, improve service organization, and enhance care delivery throughout a person’s life.

The Global Burden of Diseases, Injuries, and Risk Factors Study 2021 (GBD 2021) is a comprehensive international initiative that analyzes and synthesizes global health data. It serves as a vital resource for studying the burden of mental disorders in children, adolescents, and youth at global, regional, and national levels. Many studies based on the GBD framework have explored the burden of mental disorders, focusing on specific time points (6), regions (1, 7, 8), and sub-disorders (9–11). Some have also conducted time series analyses using data collected before 2019 (2, 12). Such longitudinal studies tracking individuals from childhood into adulthood reveal that many disorders, such as attention-deficit/hyperactivity disorder (ADHD), anxiety disorders, and conduct disorders identified early in life, exhibit significant persistence and can profoundly shape long-term outcomes (2, 12). Additionally, there is a notable gap in research specifically addressing mental disorders in children, adolescents, and youth. Using GBD 2019, Kieling et al. (2) estimated the prevalence and disability from mental disorders across children, adolescents, and youth globally. Li et al. understood the burden of mental disorders among children and adolescents considering both co-morbidities and suicide in Northeastern China (6). However, critical gaps persist in the current literature. First, existing analyses are heavily reliant on pre-2019 data. Second, comprehensive analyses specifically targeting the burden across detailed developmental age groups (e.g., <5 years, 5–9 years, 10–14 years, 15–19 years, 20–24 years) remain scarce, hindering the development of age-tailored interventions. Third, the assessment of health equity (disparities in burden across populations) for these disorders among children, adolescents, and youth demands rigorous investigation using the most recent data. Fourth, understanding the relative importance of specific risk factors for major mental disorder subtypes across different global regions remains inadequately characterized. Finally, forecasts of future burden for children, adolescents, and youth mental disorders, which are essential for proactive health system planning, are conspicuously lacking.

To address these significant gaps, this study aims to generate comprehensive evidence for optimizing early-life mental health interventions globally by quantifying the disease burden, health inequities, and evolving trajectories of major mental disorders across critical developmental stages from infancy to young adulthood (<5 to 24 years). By leveraging the GBD 2021, our study estimates to (i) evaluate the global burden and health equity of several major mental disorders in children, adolescents, and youth across five critical developmental age groups (<5, 5–9 years, 10 to 14 years, 15–19 years, and 20–24 years) according to the WHO definition, (ii) identify critical temporal shifts in disease trends, (iii) ascertain the relative importance of various risk factors for key mental disorder subtypes across different global regions, and (iii) forecast the global prevalence and disability burden of these disorders in children, adolescents, and youth up to the year 2050.

## 2 Materials and methods

### 2.1 Data source and definition

All data of mental disorders were obtained from the Global Health Data Exchange (GHDx) online resource (https://vizhub.healthdata.org/gbd-results/), managed by the Institute for Health Metrics and Evaluation (IHME). GBD 2021 represents a thorough and collaborative effort aimed at assessing health loss attributed to 371 diseases, injuries, and 88 risk factors across 204 countries and territories from 1990 to 2021 (13). The methodology for data acquisition, processing, and analysis in the GBD 2021 study is detailed in previous publications (13, 14). Specially, Gaussian process regression imputed sparse data (e.g., conflict-affected regions), with uncertainty intervals reflecting imputation variance.

#### 2.1.1 Socio-demographic index (SDI)

The GBD 2021 study assessed the socio-demographic status of each location using a composite indicator called the SDI, which is calculated using three key indicators: educational attainment for individuals aged 15 and older, the total fertility rate for women under 25, and average income per person. The SDI is represented on a scale from 0 (low SDI) to 100 (high SDI), with categorizing into low, low-middle, middle, high-middle, and high SDI. Additionally, for administrative and data analysis purposes, all countries and territories were divided into 21 GBD regions based on their epidemiological similarities and geographic proximity.

#### 2.1.2 Risk factors for mental disorders

The GBD 2021 evaluated the health impacts of various behavioral, metabolic, environmental, and occupational risk factors, as well as their combinations, across 204 countries and territories from 1990 to 2021 (14, 15). This assessment measured the disability-adjusted life years (DALYs) linked to lead exposure, childhood sexual abuse, and bullying victimization for three mental disorders: anxiety disorders, depressive disorders, and idiopathic developmental intellectual disability (IDII). The population attributable fractions (PAFs) for each risk factor were calculated by gathering exposure data, relative risk estimates, and theoretical minimum risk exposure levels (TMRELs). Then, the burden of these three mental disorders due to risk factors was assessed by calculating the total DALYs and multiplying by the PAF.

### 2.2 Data processing

#### 2.2.1 Descriptive analysis

The burden of mental disorders was assessed based on the annual number of cases for prevalence and DALYs. Our analysis focused specifically on nine mental/neurodevelopmental disorders for which the GBD 2021 study provides comprehensive, age-stratified burden estimates: depressive disorders, anxiety disorders, schizophrenia, bipolar disorder, conduct disorder, autism spectrum disorders (ASD), eating disorders, IDII, and ADHD. The selected nine disorders adhered to GBD 2021’s rigorous protocols for mental disorder inclusion: (1) clinical significance: conditions with International Classification of Diseases, 10th revision (ICD-10) codes (Supplementary Table 1) and disability weights >0.1; (2) early-life burden concentration: they collectively represent the dominant contributors to mental YLDs in under-25 populations globally according to GBD methodology; (3) data completeness: age-stratified estimates available for all 5 developmental stages (<5, 5–9, 10–14, 15–19, 20–24 years). In this analysis, all age-standardized rates (ASRs) were calculated per 100,000 population, accompanied by a 95% uncertainty interval (UI), which represents the 2.5th and 97.5th percentiles derived from 1,000 iterations of the posterior distribution. The formula for ASR is as follows: $\mathrm{ASR}=\frac{\sum_{i=1}^{n}(r_{i}\times w_{i})}{\sum_{i=1}^{n}w_{i}}$, where $r_{i}$ denotes the age-specific rate for the *i*th age group and $w_{i}$ represents the weight of people in the corresponding age group within the selected reference population. All statistical analyses and data visualizations were executed utilizing R version 4.4.2 and JD_GBDR version 2.37 (Jingding Medical Technology Co., Ltd.). A two-tailed approach was adopted for all tests, with a significance threshold as *p*-values below 0.05.

#### 2.2.2 Temporal trend analysis

For temporal trend analysis, we employed Joinpoint Regression Program (Version 5.3.0) (16). This method was specifically selected to objectively identify significant inflection points in temporal trends (e.g., policy impacts or epidemiological shifts) while controlling for overfitting through Monte Carlo permutation testing. The model assumes: (1) linear trends between joinpoints, (2) uncorrelated errors, and (3) homoscedastic variance. To ensure robustness, we set the minimum and maximum allowed number of turning points and used Bayesian Information Criterion (BIC) for model selection. The annual percentage change (APC) alongside its 95% confidence interval (CI) was computed through a geometrically weighted average of the various annual percentage changes observed in the regression analysis. Then, the regression coefficients for each interval were weighted to derive the average annual percentage change (AAPC) and its corresponding 95% CI, summarizing trends over a specified fixed time interval. An upward trend is identified when both the AAPC estimate and its lower limit of the 95% CI exceed zero, vice versa.

#### 2.2.3 Decomposition analysis

Decomposition analysis was utilized to visually quantify the contributions of three independent factors to the overall changes in disease burden, i.e., age structure, population growth, and epidemiological changes. The methodology of decomposition has been elaborated in prior studies (17). The key assumptions of this method included additive independence of factors and constant proportional contributions over time.

#### 2.2.4 Cross-country inequalities analysis

In our study, slope index of inequality (SII) and concentration index (CII) were employed for quantifying the distribution inequality in the burden of mental disorders across different countries, utilizing the SDI for ranking purposes (18). The SII was determined through regression analysis of the country-level prevalence and DALY due to mental disorders across all age demographics, defined by the midpoint of the cumulative class range of the population ranked by SDI. The CII was computed by fitting the observed cumulative relative distribution of mental disorder burdens against the SDI, where positive CII values indicated that the burden were more concentrated within countries exhibiting higher SDI (19). A concentration curve illustrates the cumulative percentage of ASR in the burden on the *y*-axis against the cumulative percentage of the population, ranked by SDI on the *x*-axis. If the concentration curve lies below the line of equality (the 45-degree line), it implies that the burden of mental disorder is more pronounced in high-SDI nations; otherwise, it is more substantial in low-SDI nations.

#### 2.2.5 Frontier analysis

Frontier analysis delineates countries and regions exhibiting the optimal health outcomes (e.g., life expectancy, mortality rates, or disease burden) relative to their specific levels of social and demographic development (e.g., SDI). We implemented non-parametric data envelope analysis to generate a nonlinear frontier, guided by detailed methodologies outlined in previous literature (20). This frontier signifies the minimal achievable burden, as determined by the development status. The effective difference is defined as the gap between the observed ASR for a given country and its corresponding frontier, representing a potential health gain that remains unrealized given the current developmental level of that country or region.

#### 2.2.6 Projection analysis

For forecasting, we implemented a Bayesian age-period-cohort (BAPC) model (21) to disentangle developmental (age), temporal (period), and generational (cohort) effects, which critical for projecting youth mental disorders. The BAPC model incorporated integrated nested Laplace approximations for comprehensive Bayesian inference, and assumes that temporally adjacent age, period, and cohort effects are similarly influential, employing a second-order random walk to smooth the priors associated with these three effects. The prior information of unknown parameters, along with sample information, are estimated to obtain a posterior distribution, from which inferences about the unknown parameters are made. Model fitness was rigorously assessed through comparing posterior predictive observed and simulated data.

## 3 Results

### 3.1 Overview global burden

**Figure 1:**
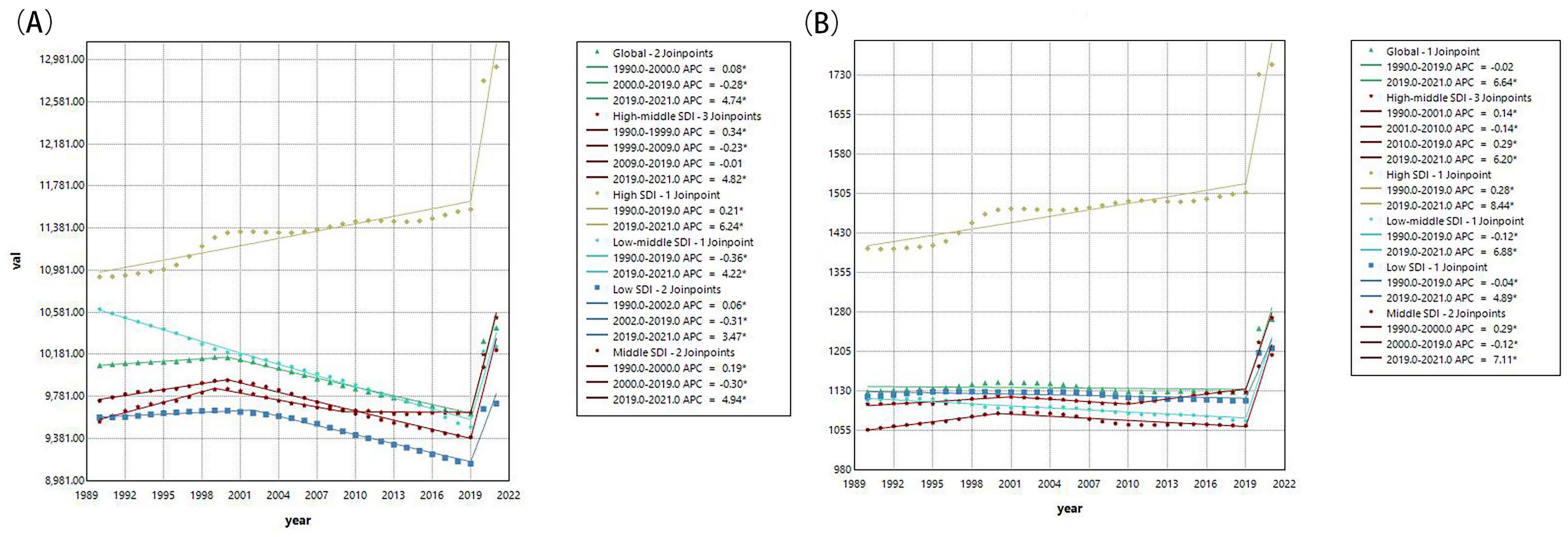
The changes of ASPR and ASDR for mental disorders from 1990 to 2021. **(A)** Changes of age-standardized prevalence rate; **(B)** Changes of age-standardized DALY rate. ASPR, age-standardized prevalence rates; ASDR, age-standardized DALY rates; DALY, the disability-adjusted life years; APC, annual percentage change; AAPC, average annual percentage change; SDI, socio-demographic index.

An overview of the global burden in population aged under 24 years reveals a slight increase in the prevalence and DALYs related to mental disorders. The age-standardized prevalence rate (ASPR) of mental disorders increased from 10075.76 (95% UI 8578.00 to 11697.92) per 100,000 in 1990 to 10433.45 (95% UI 8925.95 to 12104.94) per 100,000 in 2021 (Table 1). The AAPC was found to be 0.15 (95% CI 0.14 to 0.16), suggesting a slight upward trajectory. Specifically, the ASPR demonstrated a decline starting in 2000, showing a more tempered decreasing trend until 2019 [APC −0.28 (95% CI −0.29 to −0.27)] before experiencing a sharp increase from 2019 to 2021 [APC 4.74 (95% CI 4.55 to 4.93); Figure 1A and Supplementary Table 2]. Globally, the age-standardized DALYs rate (ASDR) among individuals aged under 24 years was 1128.12 (95% UI 816.40 to 1493.91) per 100,000 persons in 1990 and rose to 1266.19 (95% UI 905.51 to 1690.17) per 100,000 persons in 2021 (Table 1). The AAPC was 0.40 (95% CI 0.29 to 0.51), reflecting a positive trend with a flattening decline observed from 1990 to 2019 [APC −0.02 (95% CI −0.04 to 0.01)], followed by a rapid increase from 2019 to 2021 [APC 6.64 (95% CI 4.88 to 8.42); Table 1 and Figure 1B].

**Table 1:** The burden and temporal trends of mental disorders by sex, SDI, and global in 1990 and 2021.

Location	Prevalence					DALYs				
	Number (95% UI)		ASPR (95% UI)		AAPC (95% CI)	Number (95% UI)		ASDR (95% UI)		AAPC (95% CI)
	1990	2021	1990	2021		1990	2021	1990	2021	
Global	277736208.65 (236539575.95,322310108.38)	346358694.87 (296686630.84,401435795.20)	10075.76 (8578.00,11697.92)	10433.45 (8925.95,12104.94)	0.15 (0.14, 0.16)	31207306.29 (22591423.08,41316442.56)	42264639.03 (30223443.26,56412619.83)	1128.12 (816.40,1493.91)	1266.19 (905.51,1690.17)	0.40 (0.29,0.51)
Sex										
Female	130424137.12 (110486446.26,152099827.04)	166883701.28 (141730054.33,194663687.35)	9621.63 (8145.16,11226.03)	10292.05 (8728.78,12017.74)	0.27 (0.25,0.29)	15897249.67 (11346064.78,21289006.92)	21819618.31 (15408423.05,29369989.09)	1166.88 (832.51,1563.25)	1336.77 (943.97,1799.68)	0.48 (0.36,0.60)
Male	147312071.53 (124969883.80,171363828.03)	179474993.59 (153779955.22,207267482.61)	10501.73 (8908.27,12218.14)	10558.42 (9035.42,12206.62)	0.04 (0.02,0.06)	15310056.62 (11148209.23,20153200.20)	20445020.73 (14732414.90,26982770.40)	1089.17 (792.85,1433.91)	1198.02 (863.28,1581.17)	0.32 (0.24,0.41)
Age										
<5 years	20209503.45 (14666318.92,25832426.94)	18064398.38 (13524120.98,22824045.64)	3259.92 (2365.77,4166.94)	2744.63 (2054.80,3467.80)		1662419.01 (1230362.45,2229564.72)	1673641.27 (1239962.98,2227660.97)	268.16 (198.47,359.64)	254.29 (188.40,338.46)	−0.17(−0.20,−0.14)
5–9 years	43328460.82 (35723224.51,52524132.37)	49316995.89 (41194475.33,59159139.81)	7425.22 (6121.91,9001.09)	7178.04 (5995.82,8610.56)		3431722.89 (2468301.95,4496325.78)	4275421.02 (3046344.90,5673881.87)	588.10 (422.99,770.54)	622.28 (443.39,825.83)	
10–14 tears	68672334.76 (58535713.82,79881387.99)	88775337.17 (75266189.92,102788136.26)	12819.59 (10927.31,14912.07)	13316.91 (11290.45,15418.93)		6997118.47 (4939451.12,9460605.94)	9799527.63 (6848122.86,13404837.85)	1306.21 (922.09,1766.08)	1470.00 (1027.27,2010.82)	
15–19 years	74428835.55 (65400132.80,83721022.85)	96099880.52 (85181745.41,108967842.95)	14329.13 (12590.91,16118.07)	15401.12 (13651.36,17463.36)		9165583.45 (6663559.84,12105692.88)	12718844.65 (9146967.48,16817965.90)	1764.57 (1282.88,2330.60)	2038.34 (1465.91,2695.27)	
20–24 years	71097074.06 (62214185.90,80351138.22)	94102082.91 (81520099.21,107696630.53)	14448.04 (12642.90,16328.61)	15758.32 (13651.34,18034.86)		9950462.47 (7289747.72,13024253.24)	13797204.46 (9942045.04,18288273.25)	2022.09 (1481.39,2646.73)	2310.48 (1664.89,3062.55)	
SDI										
High SDI	37124588.92 (32236052.19,42428444.08)	41565553.06 (35920866.10,47765832.97)	10916.10 (9451.54,12515.64)	12913.13 (11135.82,14874.98)	0.58 (0.45,0.72)	4853138.97 (3505722.57,6416219.99)	5729096.69 (4109534.13,7615752.07)	1400.49 (1010.15,1853.65)	1750.41 (1253.46,2328.87)	0.79 (0.59,0.98)
High-middle SDI	46744121.06 (40196885.98,53965871.70)	41742434.53 (35539316.09,48592876.75)	9540.08 (8177.55,11038.35)	10529.48 (8955.82,12268.44)	0.33 (0.30,0.36)	5508256.55 (3970149.39,7300870.10)	5061399.09 (3583010.81,6760377.76)	1082.66 (769.01,1445.47)	1268.59 (897.73,1694.83)	0.48 (0.44,0.53)
Middle SDI	93710359.50 (80135309.21,108748594.18)	98766237.05 (84593963.57,113999153.49)	9738.25 (8312.87,11317.89)	10220.64 (8738.44,11813.13)	0.19 (0.16,0.21)	10265731.15 (7402233.78,13612984.06)	11665981.48 (8313860.86,15587853.84)	1055.59 (760.69,1400.32)	1197.92 (853.73,1600.63)	0.46 (0.40,0.53)
Low-middle SDI	71389796.41 (59179180.48,84114255.98)	99002370.64 (83685686.92,115151346.50)	10609.87 (8837.18,12466.25)	10256.60 (8651.31,11946.64)	−0.07(−0.11,−0.03)	7321490.48 (5257751.17,9751732.01)	11753047.31 (8394010.06,15695528.11)	1110.61 (797.85,1477.96)	1206.98 (862.23,1612.06)	0.32 (0.27,0.37)
Low SDI	28534822.76 (23619401.02,33620329.74)	65013738.42 (54747679.11,76190799.40)	10256.60 (8651.31,11946.64)	9712.76 (8190.02,11371.76)	0.19 (0.05,0.09)	3231868.82 (2316204.50,4315723.11)	8022370.55 (5736706.08,10765635.15)	1118.18 (801.01,1493.11)	1210.81 (866.04,1624.25)	0.27 (0.20,0.35)
Regions										
High-income Asia Pacific	6586618.42 (5714807.03,7522664.16)	4451782.68 (3860038.74,5088551.39)	9304.13 (8041.38,10676.77)	10020.89 (8672.03,11486.67)	0.24 (0.22,0.26)	859645.31 (628814.83,1122728.07)	614308.52 (445239.99,811002.87)	1196.42 (872.99,1564.35)	1359.57 (982.82,1796.91)	0.42 (0.40,0.44)
High-income North America	12639066.03 (10988095.50,14456196.80)	17901703.64 (15583911.48,20493286.15)	11723.97 (10161.08,13453.01)	14442.17 (12530.45,16594.29)	0.73 (0.55,0.91)	1633080.15 (1178639.07,2147589.23)	2508626.02 (1799772.70,3311339.03)	1485.33 (1071.21,1953.61)	1986.01 (1423.73,2621.59)	1.10 (0.89,1.32)
Central Asia	2850256.34 (2413104.15,3327141.17)	3391312.03 (2867770.05,3990906.76)	7869.01 (6674.23,9170.35)	8411.46 (7123.36,9884.81)	0.26 (0.20,0.32)	340849.92 (244677.61,454091.65)	426051.04 (303854.07,570324.33)	946.05 (679.17,1260.28)	1063.04 (758.38,1423.09)	0.42 (0.37,0.47)
East Asia	57375114.41 (48740715.33,66921419.25)	40656106.45 (34229390.79,47957648.52)	9118.33 (7704.54,10683.51)	9348.40 (7871.00,11022.26)	0.04(−0.04,0.12)	6152683.59 (4425155.46,8164878.77)	4025853.05 (2855250.31,5355505.29)	947.31 (678.62,1258.50)	929.37 (659.64,1236.31)	−0.09(−0.17,−0.02)
South Asia	69972995.72 (56593934.03,83425441.82)	93455161.17 (78257836.99,109464344.16)	11176.10− (9087.03,13292.06)	10478.53 (8723.24,12313.76)	−0.17(−0.21,−0.13)	6502341.94 (4638307.96,8705408.16)	10237059.40 (7311473.98,13665756.29)	1060.71 (757.58,1417.81)	1121.53 (800.57,1498.65)	0.20 (0.09,0.32)
Southeast Asia	22726829.03 (19207589.27,26517244.37)	25947110.85 (22126951.71,30156130.77)	8577.75 (7249.51,10007.99)	8703.78 (7407.30,10129.42)	0.05 (0.04,0.05)	2517895.73 (1806035.37,3356475.94)	3258468.23 (2313358.01,4376176.17)	952.75 (683.63,1269.75)	1082.70 (768.40,1454.25)	0.41 (0.40,0.42)
Australasia	1297148.36 (1118043.07,1503152.29)	1648399.41 (1421503.64,1911577.36)	15467.39 (13294.78,17997.59)	16389.77 (14118.71,19018.95)	0.21 (0.19,0.23)	167948.73 (120399.87,222750.42)	216699.84 (155757.97,290312.14)	1956.35 (1400.27,2597.44)	2120.47 (1523.27,2843.44)	0.29 (0.26,0.31)
Caribbean	2238564.04 (1880795.40,2666148.78)	2545925.09 (2113785.94,3047072.88)	11915.57 (9993.81,14217.41)	12861.89 (10658.89,15420.42)	0.29 (0.24,0.33)	234223.99 (167930.15,318044.71)	274598.47 (194065.32,375764.92)	1230.26 (881.43,1671.14)	1365.32 (964.67,1868.92)	0.39 (0.37,0.41)
Central Europe	4131234.60 (3515091.34,4824525.89)	2871457.30 (2436510.17,3365002.48)	8165.58 (6935.76,9551.41)	9155.14 (7759.81,10739.79)	0.37 (0.32,0.43)	505143.31 (362493.23,669913.61)	372743.82 (265368.97,500578.36)	995.51 (714.33,1319.99)	1180.16 (839.88,1584.68)	0.57 (0.53,0.61)
Eastern Europe	7052447.83 (6057947.13,8129709.28)	5706997.65 (4901010.76,6584418.34)	8420.02 (7226.51,9712.76)	9783.44 (8399.64,11293.41)	0.49 (0.47,0.51)	882084.37 (630806.40,1167350.42)	744011.34 (527226.02,993245.87)	1050.73 (751.15,1390.71)	1280.93 (908.40,1709.58)	0.65 (0.62,0.67)
Western Europe	17650382.12 (15216840.79,20342224.82)	17739345.85 (15036616.58,20741048.64)	12587.86 (10816.34,14552.25)	14233.90 (12044.93,16672.50)	0.52 (0.41,0.63)	2402565.79 (1716779.67,3213519.51)	2461499.61 (1739814.66,3328834.12)	1671.85 (1190.26,2239.56)	1949.90 (1375.85,2638.55)	0.63 (0.57,0.69)
Andean Latin America	2397340.80 (1990822.07,2869454.51)	3868686.63 (3199308.82,4690367.33)	10834.69 (9003.62,12960.89)	12698.37 (10493.37,15403.77)	0.65 (0.64.0.66)	260630.44 (184936.33,354218.28)	462246.39 (320293.08,635490.44)	1187.68 (843.34,1613.35)	1501.61 (1040.28,2064.11)	0.96 (0.94,0.97)
Central Latin America	8713664.62 (7422974.72,10131728.98)	11837041.39 (10066977.08,13810038.62)	8968.41 (7643.61,10423.51)	10439.17 (8861.32,12196.63)	0.58 (0.38,0.78)	1042974.88 (752000.40,1387996.42)	1528626.25 (1085269.52,2059312.97)	1501.61 (1040.28,2064.11)	1332.62 (945.08,1796.20)	0.78 (0.53,1.03)
Southern Latin America	2671799.86 (2290842.31,3094355.29)	3434572.82 (2854566.62,4087440.11)	11354.03 (9734.12,13151.35)	12848.19 (10667.29,15315.74)	0.49 (0.37,0.61)	354383.48 (253474.80,474677.31)	478725.33 (333111.30,652680.86)	1949.90 (1375.85,2638.55)	1770.56 (1230.92,2415.14)	0.62 (0.47,0.77)
Tropical Latin America	9734942.61 (8353233.75,11344252.26)	11933831.41 (10146225.01,13885425.23)	11435.10 (9814.21,13320.78)	13399.75 (11371.15,15623.98)	0.57 (0.50,0.65)	1127249.07 (804607.72,1506662.89)	1487911.86 (1045942.38,2003349.02)	1332.62 (945.08,1796.20)	1639.27 (1150.89,2208.12)	0.72 (0.64,0.81)
North Africa and Middle East	25409721.96 (21506534.49,29619927.60)	39250452.61 (32889812.39,46346749.95)	12710.13 (10782.81,14791.63)	13574.94 (11370.62,16031.56)	0.25 (0.16,0.35)	3011440.60 (2137141.17,4046430.30)	5045764.69 (3512983.71,6873756.62)	1529.15 (1085.81,2053.91)	1745.37 (1215.36,2377.35)	0.53 (0.50,0.55)
Oceania	338519.21 (279400.65,404019.94)	679105.08 (556994.17,821998.82)	8854.12 (7323.25,10553.15)	9252.21 (7600.68,11187.33)	0.13 (0.09,0.16)	39708.80 (27959.54,54009.18)	81689.36 (56425.57,113108.65)	1048.86 (738.88,1426.43)	1118.66 (772.47,1549.07)	0.18 (0.15,0.20)
Central Sub-Saharan Africa	2886016.45 (2388560.69,3445938.73)	7891503.76 (6520113.57,9485540.91)	8978.31 (7450.46,10696.59)	9681.57 (8007.74,11634.85)	0.29 (0.16,0.42)	400048.85 (279646.25,543726.88)	1094789.89 (769332.03,1513755.03)	1265.32 (884.00,1720.89)	1362.68 (957.72,1886.16)	0.29 (0.15,0.43)
Eastern Sub-Saharan Africa	9890929.08 (8323205.02,11562477.31)	23838729.57 (20108512.24,27934859.69)	8548.90 (7226.80,9957.30)	9121.85 (7698.77,10683.49)	0.22 (0.19,0.25)	1288981.74 (920321.53,1724762.18)	3258766.16 (2311415.21,4388744.63)	1135.32 (810.99,1518.52)	1255.40 (890.82,1690.59)	0.34 (0.31,0.37)
Southern Sub-Saharan Africa	2417683.04 (2072783.02,2794495.32)	3597035.80 (3066288.04,4175916.83)	7868.69 (6750.59,9088.87)	9303.57 (7928.87,10804.03)	0.59 (0.54,0.64)	332861.15 (238542.68,443536.53)	513248.85 (364061.63,688864.95)	1087.63 (779.75,1449.08)	1327.27 (941.49,1781.19)	0.73 (0.66,0.80)
Western Sub-Saharan Africa	8754934.14 (7368275.62,10258985.33)	23712433.68 (20045662.25,27802193.80)	7780.57 (6576.83,9085.86)	8009.02 (6784.55,9375.59)	0.14 (0.04,0.25)	1150564.43 (819830.83,1536856.87)	3172950.92 (2252557.93,4237296.10)	1036.22 (739.02,1383.26)	1082.66 (769.01,1445.47)	0.21(0.08,0.34)

**Figure 2:**
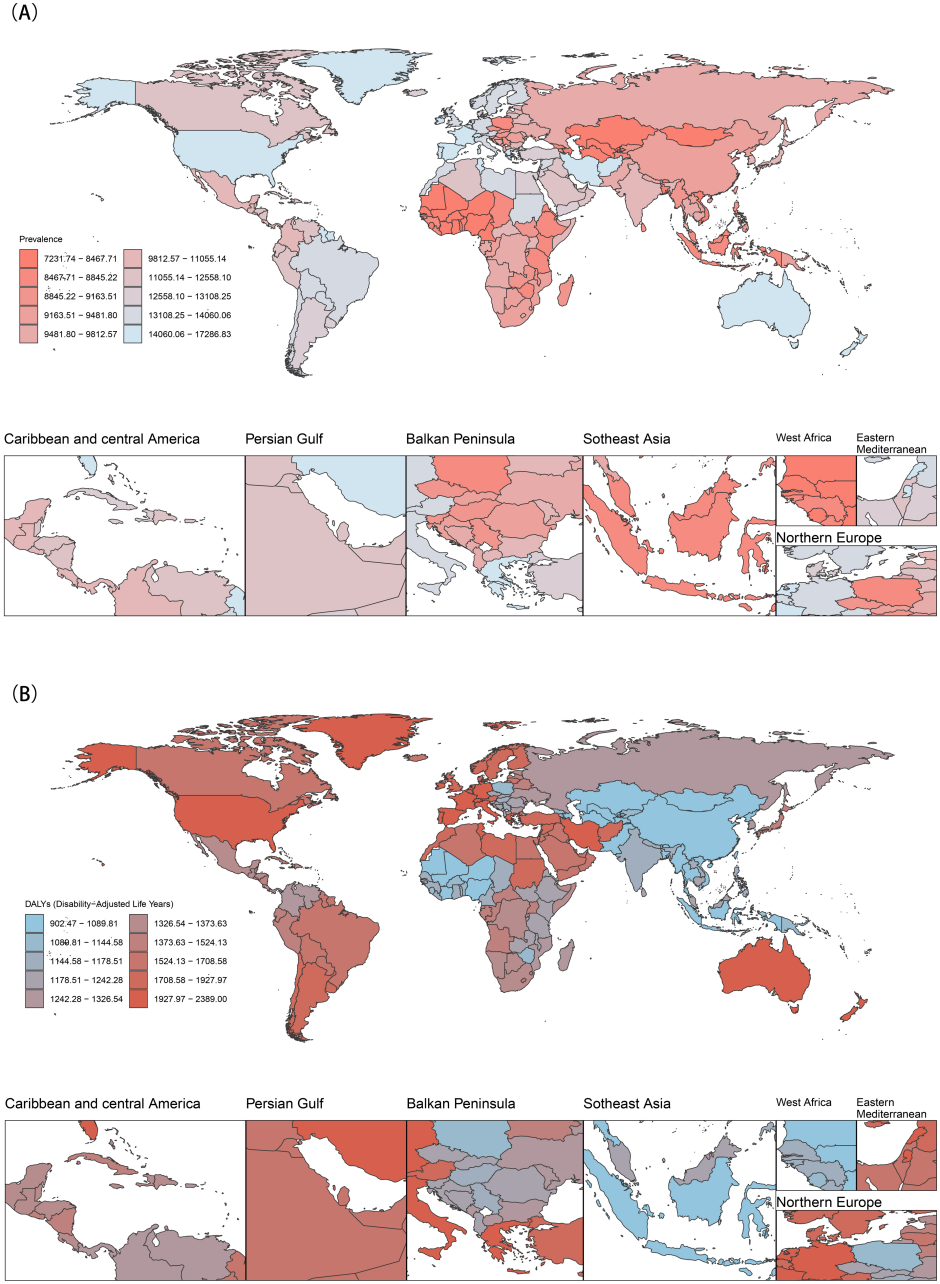
Global burden of mental disorders in 2021. **(A)** Age-standardized prevalence rates; **(B)** Age-standardized DALY rates.

As showed in Table 1, among individuals aged under 24 years, higher SDI tend to have higher ASPR and ASDR, with the highest found in high SDI region. The most pronounced increases in both ASPR and ASDR were also identified in high SDI regions, followed by high-middle SDI regions (Supplementary Figure 1). In 2021, Australasia consistently recorded the highest ASPR and ASDR for mental disorders, and Western Sub-Saharan Africa exhibited the lowest ASPR and ASDR. Over the past three decades, both ASPR and ASDR has shown an upward trend in the majority of the 21 GBD regions, with the exception of South Asia and East Asia (Supplementary Table 2). An overall upward trend was observed in 204 countries and territories regarding both ASPR and ASDR. Among them, Spain, Iran and Portugal showed the highest ASPR (Figure 2A), while Taiwan (Province of China), Bangladesh, and Uzbekistan reported the highest ASDR in 2021 (Figure 2B).

In a word, the global ASPR and ASDR of mental disorders in population aged under 24 years increased slightly from 1990 to 2021. Both metrics declined moderately until 2019 but surged sharply during 2019–2021, coinciding with the COVID-19 pandemic. High SDI regions bore the highest burden, with Australasia having the highest rates and Western Sub-Saharan Africa the lowest in 2021.

### 3.2 The sex and age pattern

In 2021, The ASPR among individuals aged under 24 years was higher in males [10558.42 (95% UI 9035.42 to 12206.62)] compared to females [10292.05 (95% UI 8728.78 to 12017.74)] globally (Supplementary Figure 3) and five SDI regions, except for high SDI region (Table 1 and Supplementary Figure 4A). This trend that was also reflected across numerous regions (Supplementary Figure 4B). However, in Western Europe, Tropical Latin America, and Southern Latin America, the ASPR for females aged under 24 years significantly exceeded that of males. On the contrary, the ASDR for females aged under 24 years [1336.77 (95% UI 943.97 to 1799.68)] surpassed that of males globally and among most regions (Table 1 and Supplementary Figures 4C,D). As age increases, the burden of mental disorders escalated, with a faster increase observed in females compared to males in population aged under 24 years (Supplementary Figure 4). In the population under 15 years, males displayed a higher burden for mental disorders than in females, while the burden were higher in females among population in 15–24 aged groups (Supplementary Figure 5).

Globally, males aged under 24 years had higher ASPR, while females aged under 24 years had higher disability burden. This sex difference varied regionally, that females aged under 24 years in Western Europe and parts of Latin America had higher ASPR. The burden escalated with age, rising faster in females. Males under 15 had higher burden than females, but females aged 15–24 faced the greatest burden.

### 3.3 The burden of nine major mental disorders

**Figure 3:**
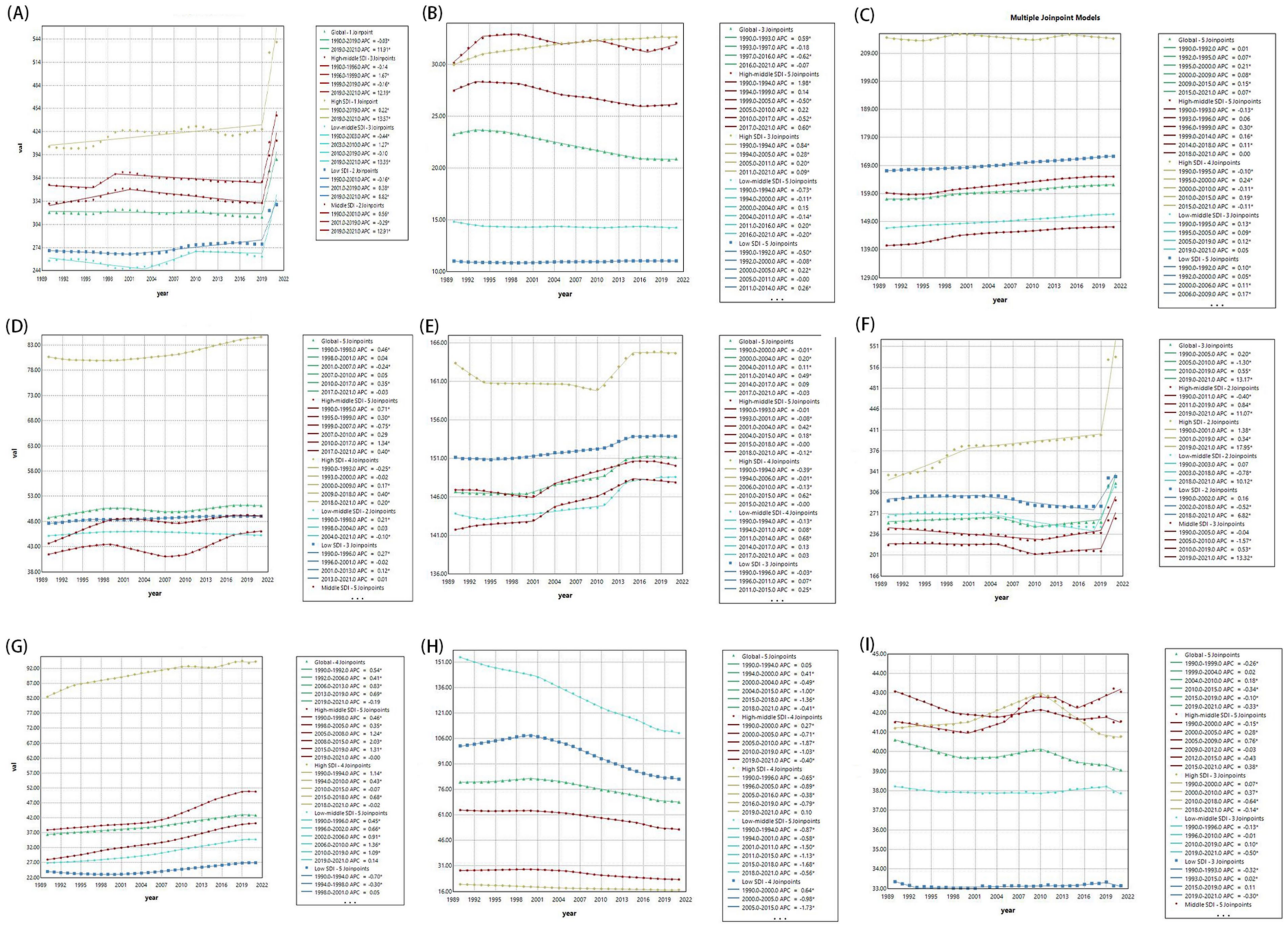
Global and five SDI regions changes of age-standardized DALY rates of nine mental disorders from 1990 to 2021. **(A)** Anxiety disorders; **(B)** Attention-deficit/hyperactivity disorder; **(C)** Autism spectrum disorders; **(D)** Bipolar disorder; **(E)** Conduct disorder; **(F)** Depressive disorders; **(G)** Eating disorders; **(H)** Idiopathic developmental intellectual disability; **(I)** Schizophrenia. APC, annual percentage change; AAPC, average annual percentage change; SDI, socio-demographic index.

**Figure 4:**
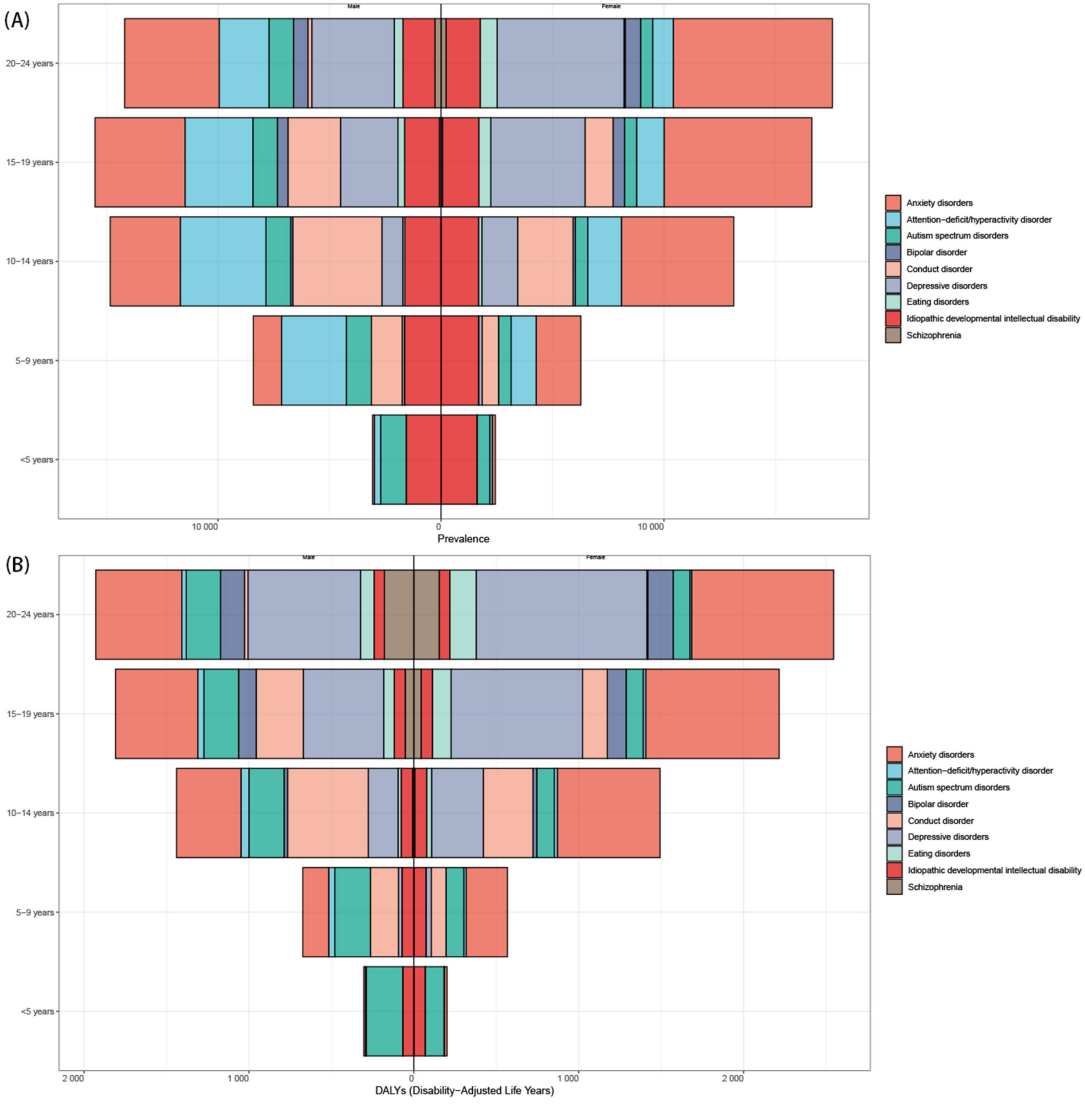
The distributions of ASPR and ASDR for mental disorders from 1990 to 2021. **(A)** Global prevalence by mental disorders, sex, and age in 2021; **(B)** Global DALYs distribution by mental disorders, sex, and age. ASPR, age-standardized prevalence rates; ASDR, age-standardized DALY rates; DALY, the disability-adjusted life years.

**Figure 5:**
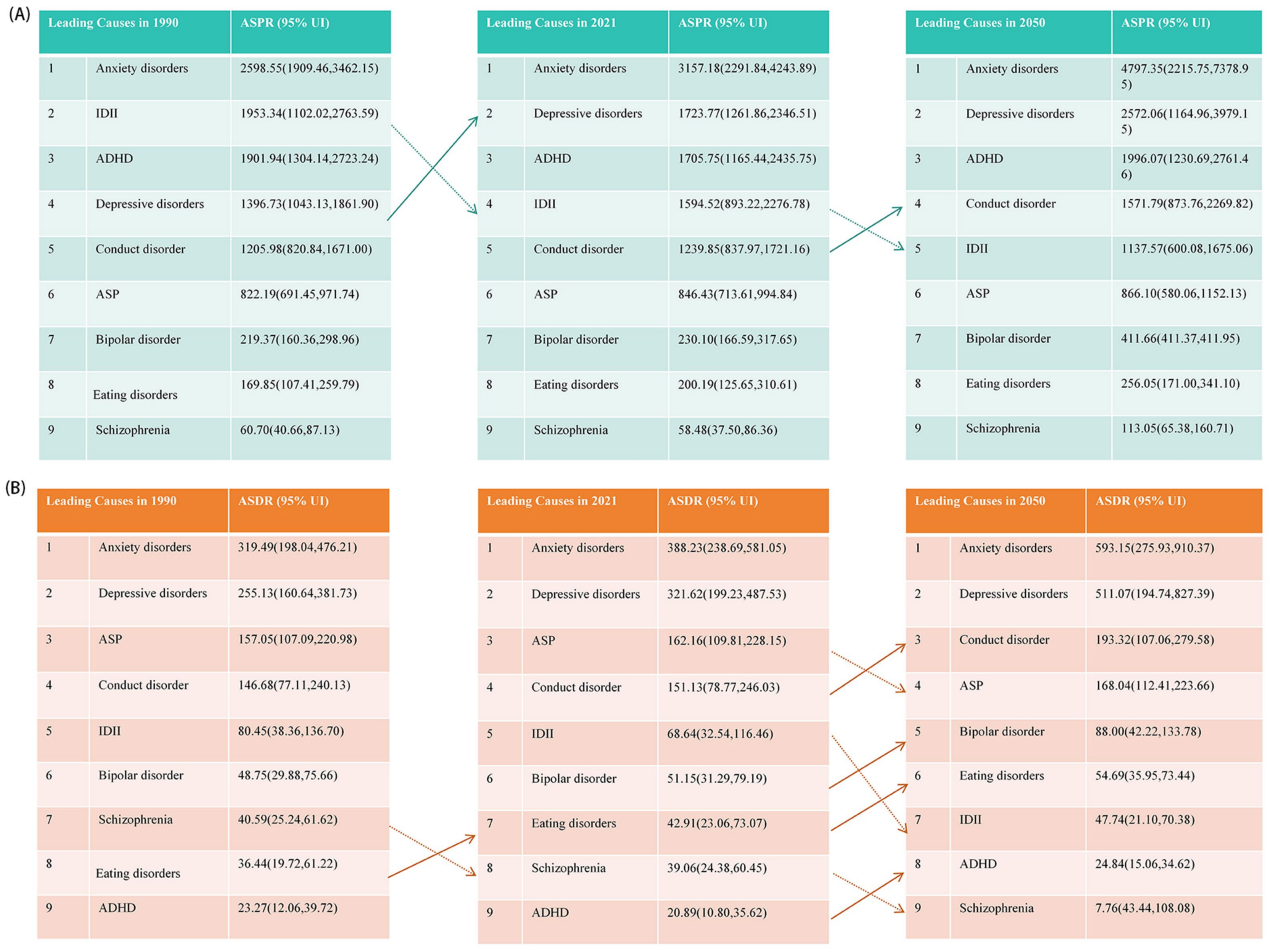
The categories of mental disorders, ranked by ASPR and ASDR from 1990 to 2050. **(A)** For ASPR; **(B)** For ASDR. ASPR, age-standardized prevalence rates; ASDR, age-standardized DALY rates; DALY, the disability-adjusted life years. ADHD, attention-deficit/hyperactivity disorder; ASD, autism spectrum disorders; IDII, idiopathic developmental intellectual disability.

From 1990 to 2021, the burden of nearly all nine mental disorders in population aged under 24 years was increase, except for ADHD, IDII, and Schizophrenia (Figure 3 and Supplementary Figure 6). Globally, anxiety and depressive disorders remained the predominant contributors to the prevalence and DALYs of mental disorders, especially among female aged 15–24 years (Figure 4). Although the ASPR of IDII and ADHD among individuals aged under 24 years were higher, the ASDR were lower than that of ASD (Figure 5 and Supplementary Figure 7). It is worth noting that the burden of IDII in population aged under 24 years was especially high in low SDI regions, such as South Asia, Southeast Asia, and North Africa and Middle East (Supplementary Figure 7). In addition, the distribution of the nine disorders in population aged under 24 years revealed distinct patterns that vary over sex, age and location. In general, females had higher ASPR and ASDR for anxiety and depressive disorders, whereas males had higher burden for ASD, conduct disorder, and ADHD, especially in individual aged under 9 years (Supplementary Figures 8, 9). The burden of nine mental disorders among 204 countries and territories was showed in Supplementary Figures 10–12. In summary, most disorders increased in burden since 1990, except ADHD, intellectual disability/idiopathic developmental delay (IDII), and schizophrenia. IDII burden was highest in low-SDI regions. Sex differences were evident. Females had higher anxiety and depression burden, while males had higher ASD, ADHD, and conduct disorder burden.

### 3.4 Decomposition analysis

**Figure 6:**
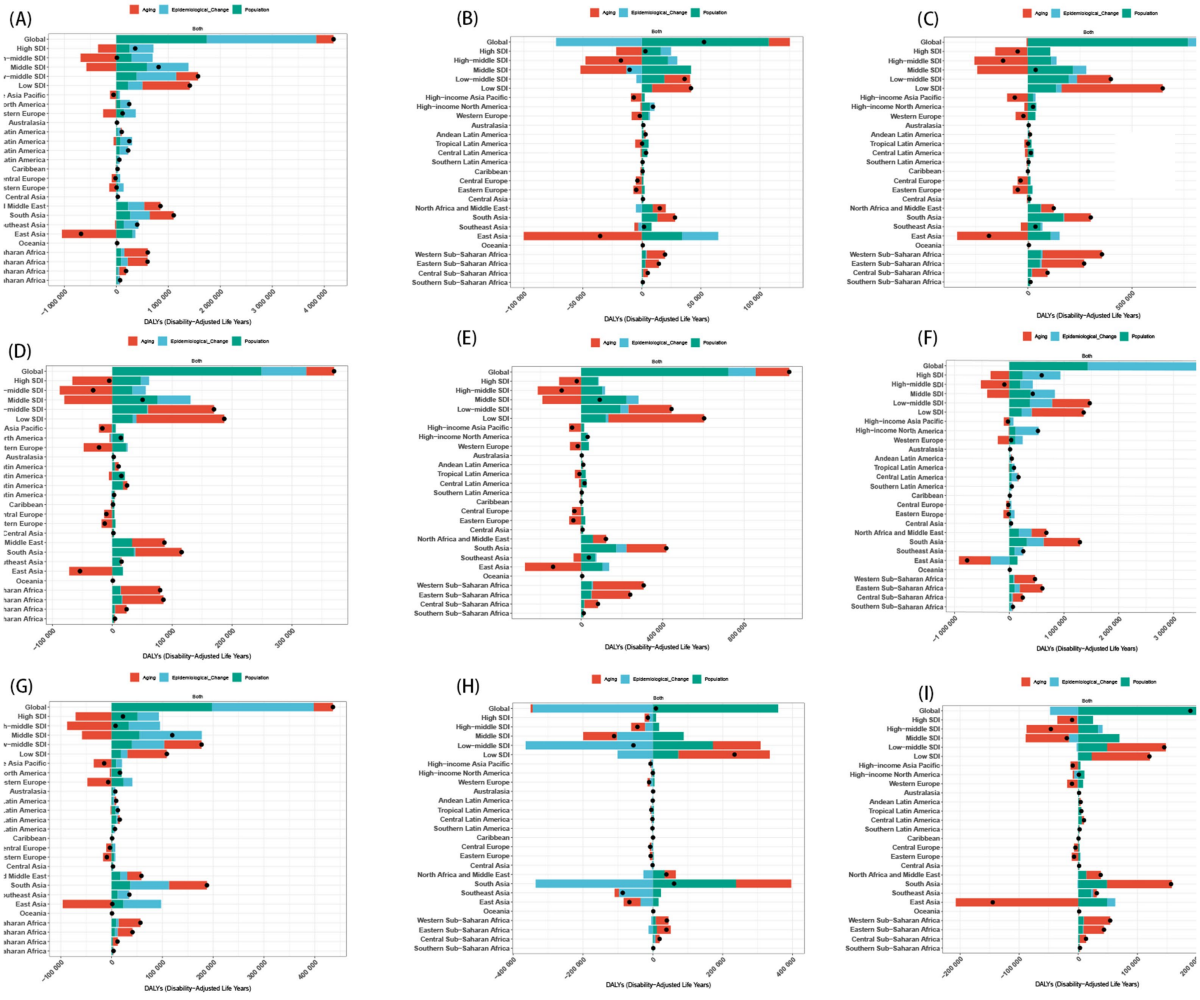
Change in DALY of nine mental disorders decomposed by three population-level determinants: aging, population and epidemiological change at the global level and various regions. **(A)** Anxiety disorders; **(B)** Attention-deficit/hyperactivity disorder; **(C)** Autism spectrum disorders; **(D)** Bipolar disorder; **(E)** Conduct disorder; **(F)** Depressive disorders; **(G)** Eating disorders; **(H)** Idiopathic developmental intellectual disability; **(I)** Schizophrenia. The black dots indicate the total value of change attributable to all three components. SDI, socio-demographic index.

Between 1990 and 2021, population growth, aging and epidemiological changes contributed 73.13%, 10.31%, and 16.57% to the increased prevalence of mental disorders in population aged under 24 years (Supplementary Figure 13A), and attributed 53.24%, 8.37%, and 38.39% to the increased DALYs of mental disorders (Supplementary Figure 13B and Table 4). Notably, a decline in overall burden among individuals under 24 years was observed in the high-middle SDI regions (such as East Asia, High-income Asia Pacific, Eastern Europe, and Central Europe), where the age structure exerted the most significant influence (334.95% and 462.54% respectively; Supplementary Table 4). When examined by sex, the impact of population growth was found to be more pronounced in males aged under 24 years, whereas epidemiological changes had a greater effect on females aged under 24 years (Supplementary Table 4). The contributions of the three population-level determinants to the burden of the 9 mental disorders among population aged under 24 years revealed significant disparities among the disorders. Globally, excepted for IDII, other 8 subtypes exhibited an increased prevalence and DALY after accounting for all factors, where the population growth exerted the most significant influence (Figure 6, Supplementary Figure 14 and Table 5). In short, population growth and epidemiological changes primarily drove increased ASPR and ASDR in population aged under 24 years. High-middle SDI regions (e.g., East Asia) saw reduced burden due to aging populations. Males aged under 24 years were more affected by population growth, while epidemiological changes impacted females more.

### 3.5 Mental disorders association with SDI quintile

**Figure 7:**
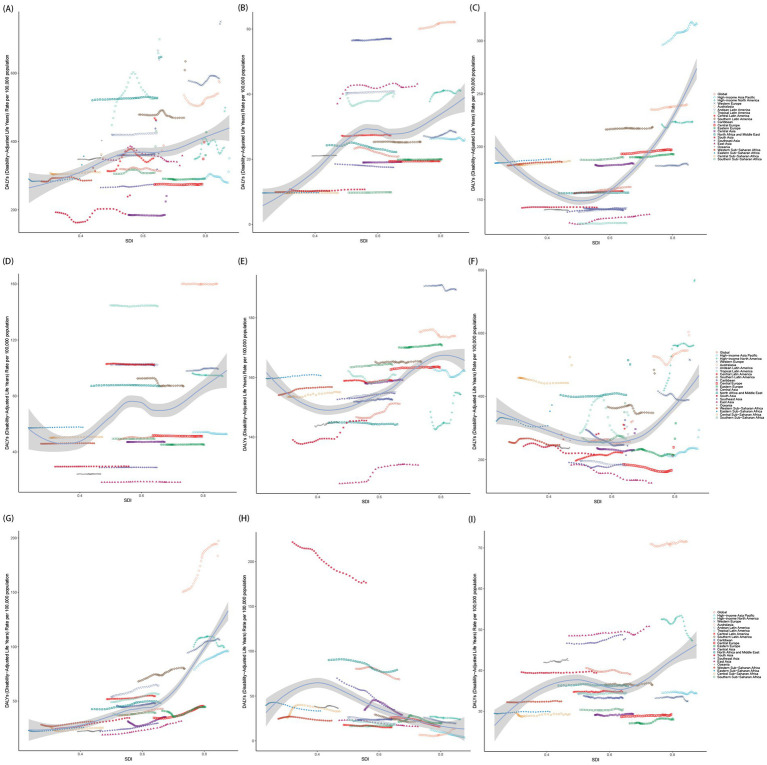
The correlation between age-standardized DALY rates of mental disorder subtypes and SDI in 21 GBD regions. **(A)** Anxiety disorders; **(B)** Attention-deficit/hyperactivity disorder; **(C)** Autism spectrum disorders; **(D)** Bipolar disorder; **(E)** Conduct disorder; **(F)** Depressive disorders; **(G)** Eating disorders; **(H)** Idiopathic developmental intellectual disability; **(I)** Schizophrenia. SDI, socio-demographic index.

The burden of mental disorders and subtypes among population aged under 24 years fluctuated depending on the SDI. A weaker positive correlation was identified between the SDI and the burden of mental disorders since 1990 (*r* = 0.34, *p* < 0.001; Supplementary Table 6), indicating that regions with elevated SDI were generally associated with higher ASPR and ASDR (Supplementary Figure 15), with the most substantial increases occurring in regions with an SDI exceeding 0.7. For 9 sub-disorders, except for IDII, other 8 mental disorders among population aged under 24 years displayed numerically higher ASPR and ASDR in higher SDI regions compared to their lower SDI counterparts, with eating disorders exhibiting the strongest correlation (*r* = 0.73, *p* < 0.001; Figure 7 and Supplementary Figure 16). Additionally, the relationship between SDI and mental disorders burden among population aged under 24 years was analyzed through the Spearman rank correlation test across the 204 countries and territories, with *p*-values below 0.05 indicating statistical significance (Supplementary Figures 17–19). All in all, higher SDI regions had significantly higher ASPR and ASDR in population aged under 24 years, with the steepest increases in regions (SDI > 0.7). All disorders except IDII showed higher burden in high-SDI areas, with eating disorders exhibiting the strongest correlation.

### 3.6 Cross-country inequalities analysis

**Figure 8:**
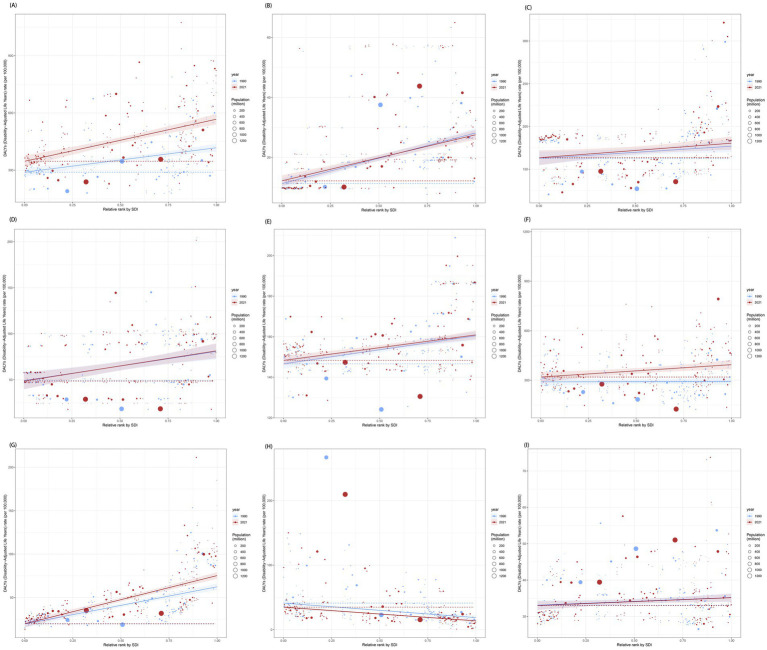
Health inequality regression curves for the DALYs of nine mental disorders. **(A)** Anxiety disorders; **(B)** Attention-deficit/hyperactivity disorder; **(C)** Autism spectrum disorders; **(D)** Bipolar disorder; **(E)** Conduct disorder; **(F)** Depressive disorders; **(G)** Eating disorders; **(H)** Idiopathic developmental intellectual disability; **(I)** Schizophrenia.

In 1990 and 2021, a higher burden of mental disorders among population aged under 24 years was disproportionately observed among higher SDI regions, with concentration curves fell below the line of equality (Supplementary Figure 20). Compared to 1990, the absolute inequalities in the ASPR and ASDR of mental disorders among population aged under 24 years related to SDI have shifted towards greater inequality, with the SII rising from 2165.61 and 247.69 in 1990 to 3268.15 and 440.23 in 2021 (Supplementary Table 7). For nine sub-disorders, the burden of nearly all mental disorders in population aged under 24 years was predominantly observed in affluent regions, except for IDII, which burden became increasingly concentrated among lower SDI regions (Figure 8 and Supplementary Figures 21–23). In brief, inequalities widened from 1990 to 2021, with high-SDI regions bearing a disproportionately larger burden among population aged under 24 years. IDII was the only disorder increasingly concentrated in low-SDI regions.

### 3.7 Frontier analysis

**Figure 9:**
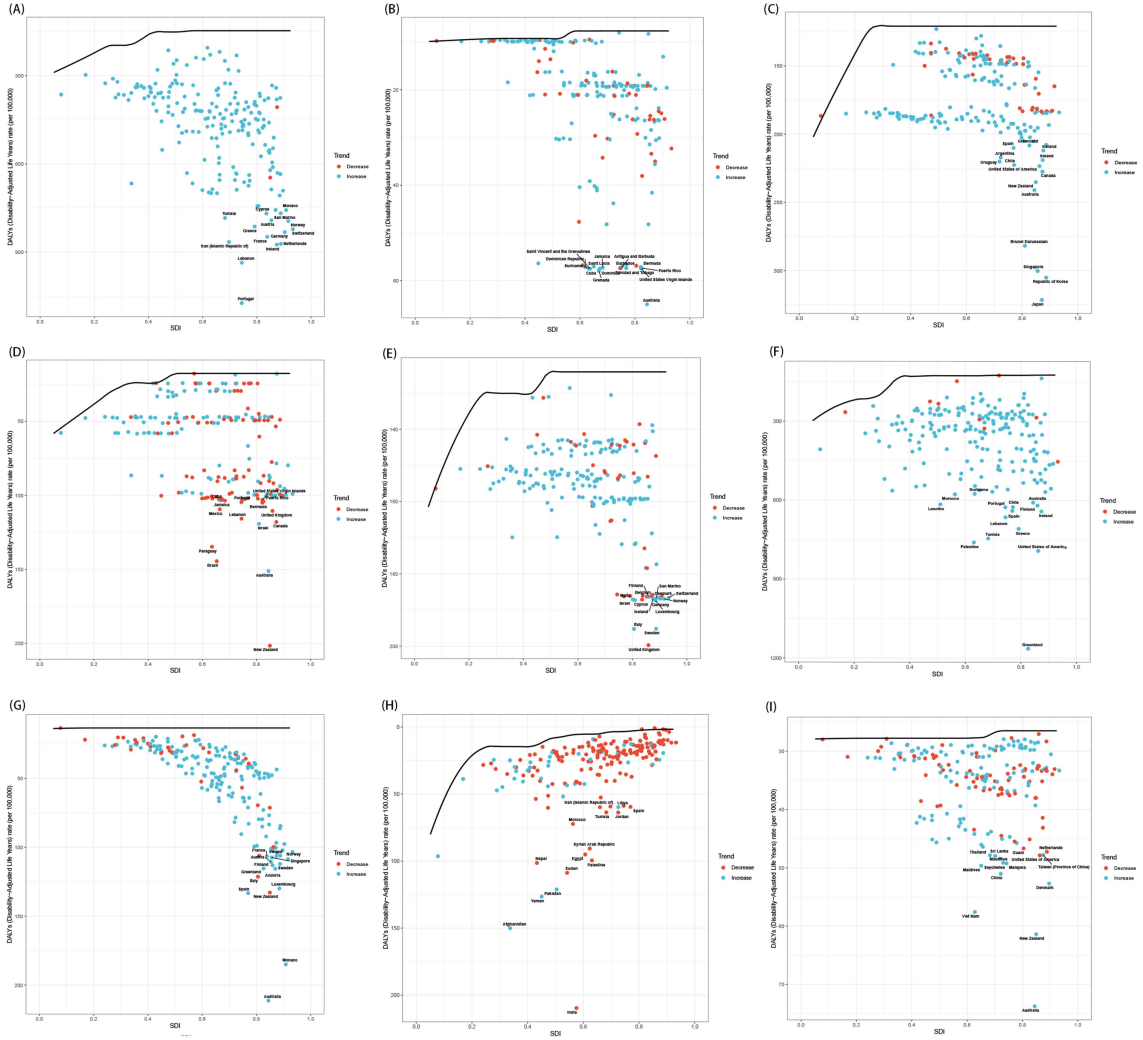
The relationship between SDI and age-standardized DALY rates for 9 mental disorders, each point represents a specific country or territory in 2021. **(A)** Anxiety disorders; **(B)** Attention-deficit/hyperactivity disorder; **(C)** Autism spectrum disorders; **(D)** Bipolar disorder; **(E)** Conduct disorder; **(F)** Depressive disorders; **(G)** Eating disorders; **(H)** Idiopathic developmental intellectual disability; **(I)** Schizophrenia.

The disease burden and effective differences in different countries among population aged under 24 years were gradually increasing with SDI quintile. Notably, the 15 high-SDI countries and territories exhibiting the most substantial actual discrepancies in potential for improvement Supplementary Figure 24. Meanwhile, San Marino, Monaco, the United States, the Netherlands, and Ireland exhibited considerable potential for advancement. Additionally, for nine sub-disorders, all analyses indicated that low SDI countries and territories, such as Mali and Niger, have basically managed to maintain relatively effective control over the disease burden among population aged under 24 years despite their resource limitations (Figure 9 and Supplementary Figures 25–27). Conversely, high SDI region, including the United States and Ireland, still possessed opportunities for further enhancement despite their advanced levels of development. We found that low-SDI countries maintained effective burden management despite resource constraints, while high-SDI countries showed the largest gaps between current and achievable burden control.

### 3.8 Risk analysis

**Figure 10:**
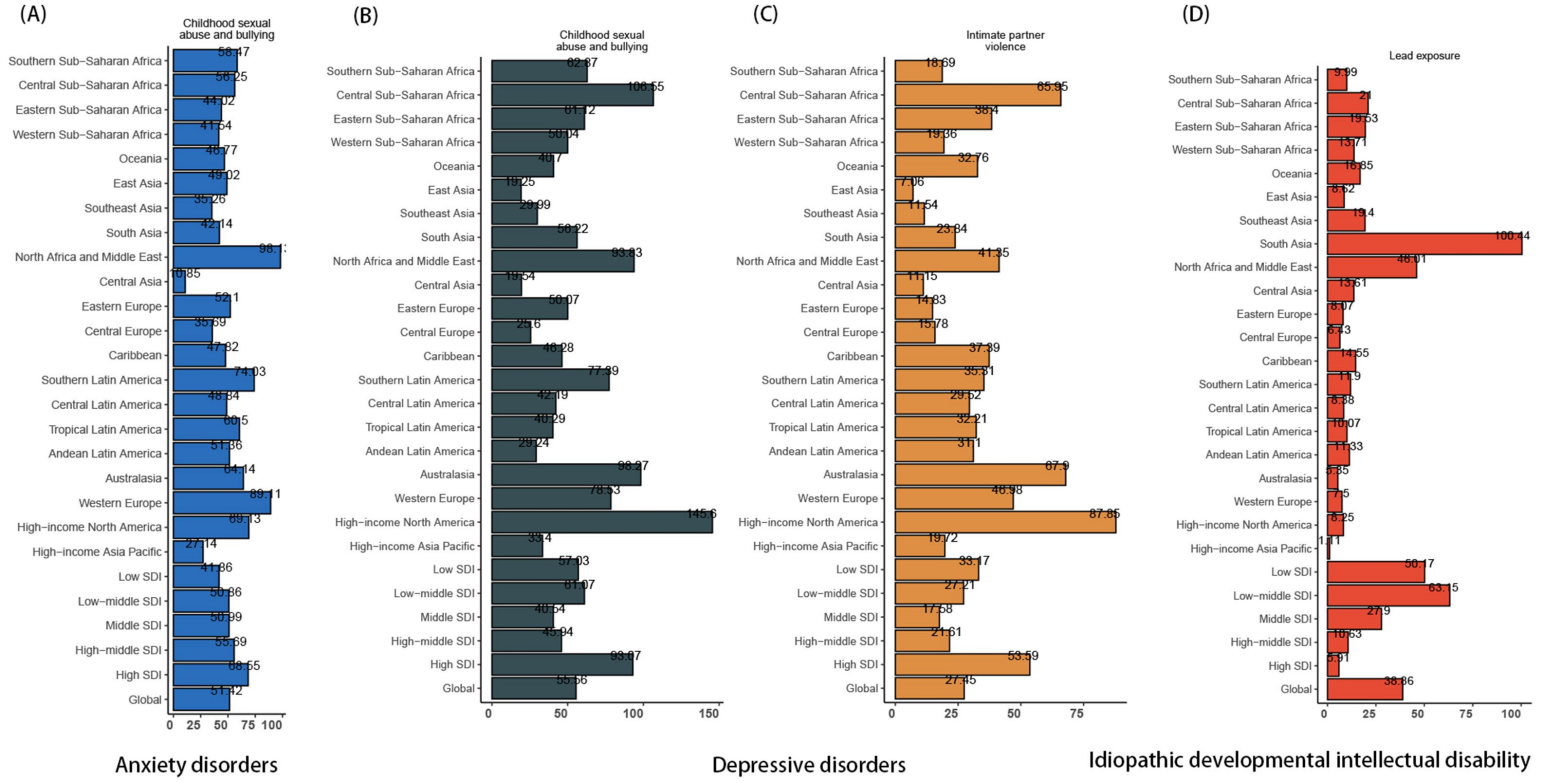
The proportion of ASDR for mental disorders attributed to risk factors across regions of different SDI levels in 2021. **(A)** Anxiety disorders; **(B)** and **(C)** Depressive disorders; **(D)** IDII. ASDR, the age-standardized DALY rates; DALYs, the disability-adjusted life years; IDII, idiopathic developmental intellectual disability; SDI, socio-demographic index.

In 2021, childhood sexual abuse and bullying [54.42% (95% CI 18.92 to 103.79%); Figure 10A] emerged as the contributor to the DALYs among population aged under 24 years that attributed to anxiety disorders, which most significantly impacted individuals aged 15–24 (Supplementary Figures 28A,B). The proportion of DALYs due to anxiety disorders attributable to childhood sexual abuse and bullying was observed to vary across SDI quintiles, which decreased from 68.55% in high SDI regions to 41.86% in low SDI regions. As showing in Figures 10B,C, two primary risk factors (i.e., childhood sexual abuse and bullying and intimate partner violence) contributing to the burden of depressive disorders in population aged under 24 years were identified, with higher attributable proportions in economically developed areas, such as High-Income North America, Australasia, and Western Europe. Remarkably, intimate partner violence only impacted female adolescents aged up 15 years (Supplementary Figures 28C–E). Other environment risk (mainly lead exposure) was identified as a risk factor for IDII DALY, which was particularly pronounced in low SDI regions among population aged under 24 years (Figure 10D). For instance, within the low-middle SDI quintile, approximately 65% of DALYs related to IDII could be traced back to lead exposure, whereas this figure drops to a mere 6% within the high SDI quintile. As an environmental/occupational risks, the attributable proportion of lead exposure were relatively consistent over sex and age (Supplementary Figures 28F,G). On the whole, childhood sexual abuse/bullying and intimate partner violence were leading risk factors for anxiety and depressive disorders, especially in high-SDI regions and females ≥15. Lead exposure drove IDII burden in low-SDI regions.

### 3.9 Projection to 2050

**Figure 11:**
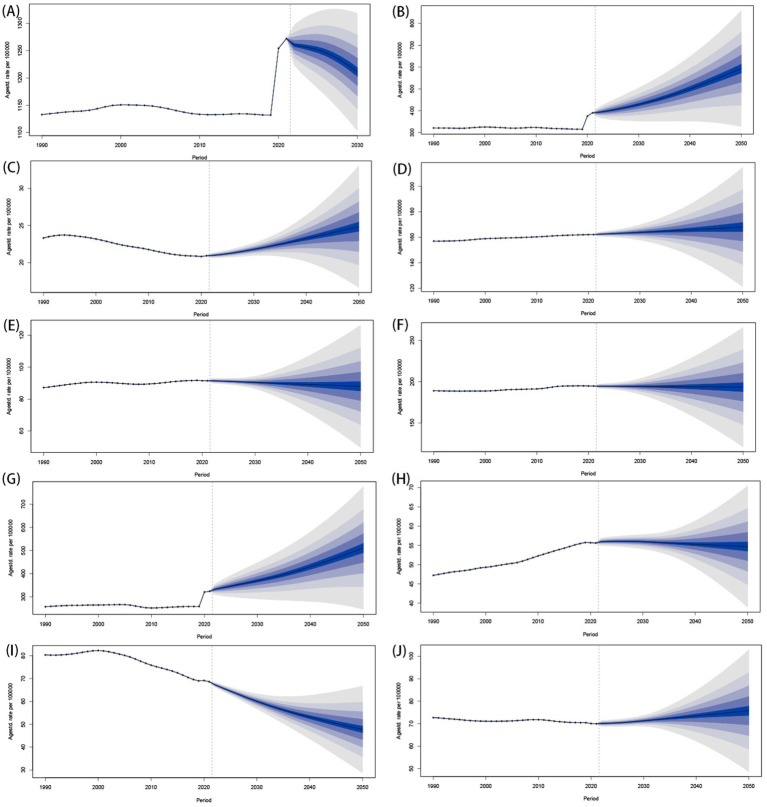
Predictions of ASDR for mental disorders globally from 2022 to 2050. **(A)** Mental disorders; **(B)** Anxiety disorders; **(C)** ADHD; **(D)** ASD; **(E)** Bipolar disorder; **(F)** Conduct disorder; **(G)** Depressive disorders; **(H)** Eating disorders; **(I)** IDII; **(J)** Schizophrenia. ASDR, the age-standardized DALY rates; DALYs, the disability-adjusted life years; ADHD, attention-deficit/hyperactivity disorder; ASD, autism spectrum disorders; IDII, idiopathic developmental intellectual disability.

Projecting towards 2050, there will been a global decrease in the burden of mental disorders among population aged under 24 years (Figure 11 and Supplementary Figure 26). The total number of prevalence cases aged under 24 years is expected to exceed 100 million (188946955.35 [95% CI 123066101.64 to 254827809.06]), with the corresponding ASPR predicting to decrease from 10433.45 (95% UI 8925.95 to 12104.94) per 100,000 in 2021 to 6120.71 (95% CI 3973.57 to 8267.85) per 100,000 by 2050 (Supplementary Table 8). In males aged under 24 years, the ASPR of mental disorders is expected to decrease from 10558.42 (95% CI 9035.42 to 12206.62) in 2021 to 6772.50 (95% CI 4543.26 to 900.90) by 2050, while females will see a drop from 10292.05 (95% CI 8728.78 to 12017.74) to 5461.36 (95% CI 3363.18 to 7559.54). Additionally, an estimated 30 million DALYs cases aged under 24 years will be attributed to mental disorders, leading to a global ASDR that continues to decline, from 1266.19 (95% UI 905.51 to 1690.17) per 100,000 in 2021 to 844.71 (95% CI 529.48 to 1159.94) per 100,000 in 2050. Moreover, both ASPR and ASDR of mental disorders are projected to decline across all age groups, with a peak among individuals aged 20–24 years (Supplementary Table 8).

The ASPRs of anxiety disorders, ADHD, and depressive disorders among children, adolescents, and youth are anticipated to constantly rise over the next three decades (Supplementary Figure 26). Meanwhile, the burden of IDII in population aged under 24 years will continue to decline, and the burden of other 5 sub-disorders (include ASP, bipolar disorder, conduct disorder, eating disorders and schizophrenia) will not experience significant fluctuations (Supplementary Figure 29). Therefore, the ASPR of conduct disorder will exceed IDII, and become one of the top four mental disorders with the highest ASPR in 2025 among population aged under 24 years (Figure 5). The predictive trend of ASDRs in sub-disorders among population aged under 24 years is consistent with ASPR. By 2025, anxiety disorders and depressive disorders are projected to remain the leading contributors of higher ASDR among population aged under 24 years (Figure 11), with respective ASDR of 593.15 (95% CI 275.93 to 910.37) and 511.07 (95% CI 194.74 to 827.39). However, the rankings of ASDRs of other disorders are expected to vary significantly compared to 2021. The conduct disorder, bipolar disorder, eating disorders, and ADHD will ascend to the 3rd, 5th, 6th, and 7th positions in 2050 (Figure 5).

Substantially, global ASPR and ASDR among population aged under 24 years are projected to decline significantly by 2050, despite rising cases due to population growth. Anxiety, ADHD, and depressive disorders will increase in prevalence, while IDII will decline. Conduct disorder will become a top 4 disorder by prevalence by 2025. DALYs will remain highest for anxiety/depression, but rankings for other disorders (e.g., conduct disorder, bipolar) will shift.

## 4 Discussion

The international community has increasingly recognized mental disorders as a significant and growing public health challenge that poses substantial difficulties for healthcare systems worldwide, especially among children, adolescents, and youth. To contribute in this endeavor, we explored and predicted the burden of mental disorders across children, adolescents, and youth, stratified by geographical and demographic factors. Over the previous three decades, there has been a notable increase in the burden of mental disorders globally among children, adolescents, and youth. In 2021, it was over 346 million children, adolescents, and youth worldwide grappled with at least one identifiable mental disorder, resulting in more than 40 million DALY cases. The age of onset for mental disorders peaks at approximately 15 years, which underscoring a critical neurodevelopmental window. This heightened vulnerability stems from the asynchronous development of key brain regions, specifically the emotion-processing limbic system and the prefrontal cortex, which governs impulse control and decision-making, combined with intense psychosocial pressures such as academic performance and social integration (22, 23). These findings compellingly argue for a reorientation of public health efforts toward proactive prevention during early adolescence (ages 10–14), including integrated school-based clinics and evidence-based digital mental health platforms, to effectively mitigate long-term negative mental health outcomes. For individuals under 5 years old, IDII constituted the primary source of disease burden, whereas, with increasing age, anxiety and depressive disorders progressively became more prominent. This finding contrasted somewhat with earlier statistics from the Centers for Disease Control (CDC), which estimated that 7.1% of children aged 2–17 have been diagnosed with anxiety, 3.2% with depression, 9.4% with ADHD, and 7.4% with behavioral disorders (24). This discrepancy may arise from fundamental methodological differences. CDC data reflect clinically diagnosed cases within the US healthcare system, while our study estimates global total burden (including undiagnosed/subclinical cases) using standardized disability weights (i.e., DALYs). In addition, CDC reports nationally representative survey data (ages 2–17), whereas our analysis specifically highlights the critical transition period from early childhood (where IDII dominates) through adolescence to youth (peak onset for anxiety/depression). Furthermore, the persistently higher burden of bipolar disorder and eating disorders is particularly alarming, as these disorders heighten the risk of negative outcomes, including diminished educational attainment, difficulties in maintaining social relationships, and an increased risk of suicide (25).

Our projections to 2050 reveal a complex future landscape for the global burden of mental disorders among children, adolescents, and youth. While the overall burden is predicted to decline significantly by 2050, this aggregate trend masks critical divergent trajectories among specific disorders. Crucially, our projection of persistently rising burdens for anxiety disorders, ADHD, and depressive disorders over the next three decades finds strong support in recent literature. In a comprehensive meta-analysis, an increases trend in the prevalence of anxiety, depression, and ADHD among youth projected to continue absent major intervention shifts (26, 27). The projected stability in burden for disorders like ASD, bipolar disorder, conduct disorder, eating disorders, and schizophrenia is broadly consistent with Erskine et al.’s (28) analysis showing relatively stable global prevalence for these conditions over time, though service demands remain high. The forecasted rise of conduct disorder to become a top contributor by ASPR ranking among population aged under 24 years, however, represents a novel projection warranting close monitoring, as it suggests changing epidemiological patterns potentially linked to evolving social determinants.

Our analysis observed a troubling shift in the burden of mental disorders among population aged under 24 years around 2019, when previously decreasing prevalence rates began to surge sharply until 2021. This trend reversal coincided with the pandemic period of COVID-19 and suggested a possible deterioration in youth mental health during this time. A number of studies have documented the increases in anxiety, depression, and related symptoms among young people during 2020–2021, especially in high-income countries. For example, Racine (29) reported a 25–30% rise in youth depression/anxiety across 11 high-income countries. Similarly, a meta-analysis of 29 studies confirmed a 2-fold increased risk of anxiety among adolescents during lockdowns (30). Although decomposition analysis indicates that overall trends were largely driven by population growth, the steep acceleration after 2019 aligns temporally with the pandemic and may reflect its specific stressors. The profound societal disruptions, including social restrictions, lockdowns, educational interruptions, heightened family stress, and pervasive uncertainty, acted as critical exacerbating factors (31, 32). While the exact causal contribution of the pandemic cannot be definitively established in our analysis, the concordance of these trends highlights the importance of further investigating how large-scale crises may interact with pre-existing vulnerabilities. These observations support recommendations that mental health support be considered an integral component of public health preparedness and recovery planning.

The results showed that the burden of mental disorders among population aged under 24 years was significantly associated with the social and economic development status. Across 21 GBD regions, Australasia, High-income North America, and Western Europe, including countries like Portugal, Greenland, and Ireland, reported increasing burdens. This pattern, showing higher burdens in many high-income regions, contrasts with some earlier assumptions that lower-income settings bear the greatest absolute burden. Some researchers contend that pronounced inequality within high-income areas fosters “relative poverty,” creating significant social hierarchies that promote social differentiation and comparison, ultimately leading to detrimental mental health outcomes (33). Allen et al. (34) found that high-SDI regions with pronounced income inequality (e.g., Gini index >35) showed 1.8-fold higher adolescent depression rates than egalitarian peers. Furthermore, the mental health of low-income populations within all regions is frequently overlooked, and their access to quality healthcare remains relatively low. The bidirectional causal relationship between poverty and mental illness is well-established, noting that individuals in poverty were more susceptible to the threat of mental health, which, in turn, can perpetuate poverty (35). Therefore, although these regions encompass a substantial proportion of the global youth population (approximately 85%), the coverage of epidemiological data, especially for vulnerable subgroups within them, remains markedly inadequate. This hampers efforts to understand the true prevalence and distribution, contributing to uncertainties in global estimations and potentially masking significant intra-regional disparities, a limitation also noted in other global burden analyses (17).

Our findings regarding sex differences indicate that females aged under 24 years were more prone to anxiety and depressive disorders, whereas disorders such as ADHD, conduct disorder, and ASP were more prevalent among males. Prior studies have suggested that these sex differences may be influenced by a combination of biological, hormonal, genetic, and socio-cultural factors (36, 37). For instance, Santos et al. (38) revealed that boys exhibit higher language and motor scores, as well as displayed greater tendencies toward hyperactivity and aggressive behavior, while girls perform better in executive function, visual reception, and facial attention, thus being more susceptible to depression and anxiety. Furthermore, socio-cultural factors and gendered social expectations profoundly shape the expression and recognition of mental health conditions. Masculine norms often discourage emotional vulnerability, which can manifest as externalizing behaviors such as aggression or impulsivity. These behaviors are more aligned with diagnoses like ADHD or ASP in males. Conversely, feminized social roles may encourage internalizing disorders such as anxiety and depression (39, 40). Concurrently, the significant risks posed by bullying to mental health, elevating risks of anxiety (1.56-fold) and depression (1.80-fold) (41), and the notable sex differences in associated burdens (e.g., higher susceptibility of females to specific forms of violence in high SDI regions) are corroborated by other large-scale studies (42, 43). While boys are more often involved in traditional school bullying, girls are disproportionately affected by cyberbullying, especially in contexts of higher digital penetration and lower gender equality (44). These nuances highlight the importance of culturally and gender-sensitive prevention strategies. Encouragingly, whole-school anti-bullying programs have been shown to reduce both perpetration and victimization. A comprehensive Campbell review of ~100 trials found that school-based anti-bullying programs reduce perpetration and victimization by about 19 and 15%, respectively (45). Randomized clinical trial evidence similarly confirms the effectiveness of such interventions across diverse global contexts (46). Together, these findings establish bullying not as a transient developmental challenge but as a modifiable and globally relevant risk factor with enduring psychiatric consequences. Scaling evidence-based, school-wide interventions, particularly those engaging bystanders and addressing gender-specific vulnerabilities, should therefore be prioritized in public health and educational agendas.

Our projection of a declining IDII burden is strongly supported by documented successes in lead abatement programs. These initiatives, such as the global phase-out of leaded gasoline, have demonstrably reduced blood lead levels and subsequently lowered intellectual disability burden. However, these policies were effectively implemented only in high-income countries. Lead exposure remains a profound environmental risk factor for neurodevelopmental and physical health in LMICs. In 2021, 85% of global IDII DALYs concentrated in low-resource regions (South Asia and North Africa/Middle East), where mean blood lead levels (BLL) in children exceed 7.8 μg/dL, which was 7-fold higher than WHO safety thresholds (47). The persistence of this problem reflects ongoing sources, including informal lead-acid battery recycling and contaminated consumer goods. Forsyth et al. (48), for instance, provided compelling evidence that turmeric in Bangladesh was adulterated with lead chromate, directly linking contaminated spices to elevated BLLs in exposed populations. These findings reveal regulatory and enforcement gaps. For example, many LMICs lack routine pediatric BLL monitoring and enforceable child-specific exposure standards. Consequently, our findings strongly advocate for targeted interventions to mitigate lead exposure as a crucial public health strategy in LMICs, aligning with priorities set by international environmental health bodies (49).

This study offers a thorough analysis of the global burden of mental disorders using GBD 2021 data, with the following limitations inherent to GBD methodology: First, the imperfection of healthcare systems in low-income countries may result in under-diagnosis, misdiagnosis, and thus, the miscalculation of disease burden. Second, although the GBD study collaborators engaged in meticulous data cleaning and sophisticated statistical modeling, the study’s reliance on indirect data and assumptions may introduce unknown biases that could further influence the present evaluation. Third, definitions, diagnostic criteria, and assessment methods for mental disorders and their specific causes have evolved and differ across time and contexts. Although the DSM and ICD classifications have been employed to maintain consistency in case definitions across studies, they may not adequately reflect all cultural contexts. Therefore, it is essential to enhance the cross-cultural applicability of case definitions. Moreover, continuously evolving classifications must be taken into account in forthcoming research endeavors. In addition, several limitations from our analytical choices persist within our current research. First, two epidemiological indicators, namely prevalence and DALYs, provide only approximate assessments regarding the burden of mental disorders. Since these disorders are not acknowledged as primary causes of mortality, data on death rates are not reported here. Second, the current analysis was conducted at a national scale without delving into specific variations at the sub-national level. Consequently, the findings may not necessarily extend to sub-national contexts. Third, the risk factors examined for each mental disorder in our research may not be exhaustive, as our criteria for inclusion were restricted to risk-outcome pairs with compelling or probable evidence of a causal association, consistent with GBD standards. Furthermore, uncertainty in PAF estimates was quantified through GBD’s 1,000 posterior draws, reflecting sampling and model stochasticity. However, formal sensitivity analyses of parameter assumptions (e.g., TMREL bounds) were not feasible within the scope of this secondary analysis. Fourth, while our projections captured the overall trend up to 2021, they do not explicitly model the unique causal pathways and magnitude of impact attributable solely to the COVID-19 pandemic on the burden of mental disorders during 2020 and 2021. Finally, this investigation relied exclusively on the GBD database and did not incorporate data from other global databases, thereby limiting the diversity of data sources.

## 5 Conclusion

This study combines decomposition, frontier, and Bayesian modeling to quantify the global mental health burden in population aged under 24 years, revealing significant mitigation potential even in high-resource settings. Adolescents and young adults (15–24 years) are identified as the most vulnerable group, with sharp rises in anxiety, depressive, and behavioral disorders. LMICs face exacerbated burdens due to resource constraints and social disparities. Critical interventions include LMIC-focused lead-abatement programs, school-based anti-bullying initiatives in high-SDI regions, and dynamic, age-specific resource allocation, particularly digital supports for youth. Future research should prioritize understanding drivers of youth mental health disparities and evaluating real-world interventions in marginalized populations.

## Data Availability

Publicly available datasets were analyzed in this study. This data can be found at: http://ghdx.healthdata.org/gbd-results-tool.
