# Supplementary material for: Global burden and trends of major mental disorders in individuals under 24 years of age from 1990 to 2021, with projections to 2050: insights from the Global Burden of Disease Study 2021
Source: Front Public Health. 2025 Sep 16;13:1635801. doi: 10.3389/fpubh.2025.1635801 (PMC12481897; doi:10.3389/fpubh.2025.1635801)
Supplement: Supplementary file 1 [file Presentation_1.zip › Supplementary Figure 1-9.DOCX]

Supplementary Figures 1


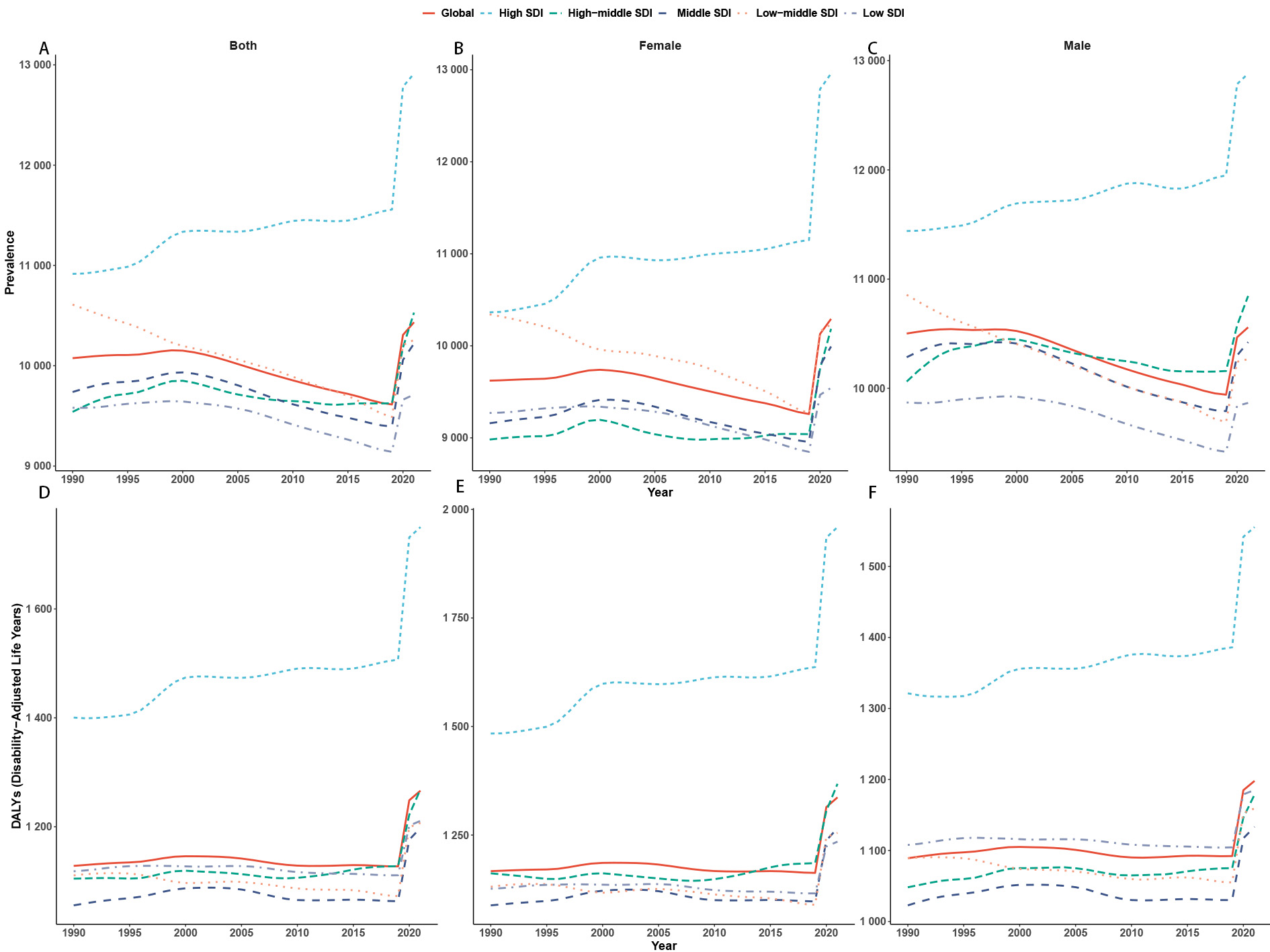


**Supplementary Figure 1.** Changes in prevalence/DALY rates by 5 SDI regions and sex. (A) ASPR of mental disorders among 5 SDI regions; (B) ASPR of mental disorders among 5 SDI regions in females; (C) ASPR of mental disorders among 5 SDI regions in males; (D) ASDR of mental disorders among 5 SDI regions; (E) ASDR of mental disorders among 5 SDI regions in females; (F) ASDR of mental disorders among 5 SDI regions in males. Abbreviation: ASPR, age-standardized prevalence rate; ASDR, age-standardized death rate.


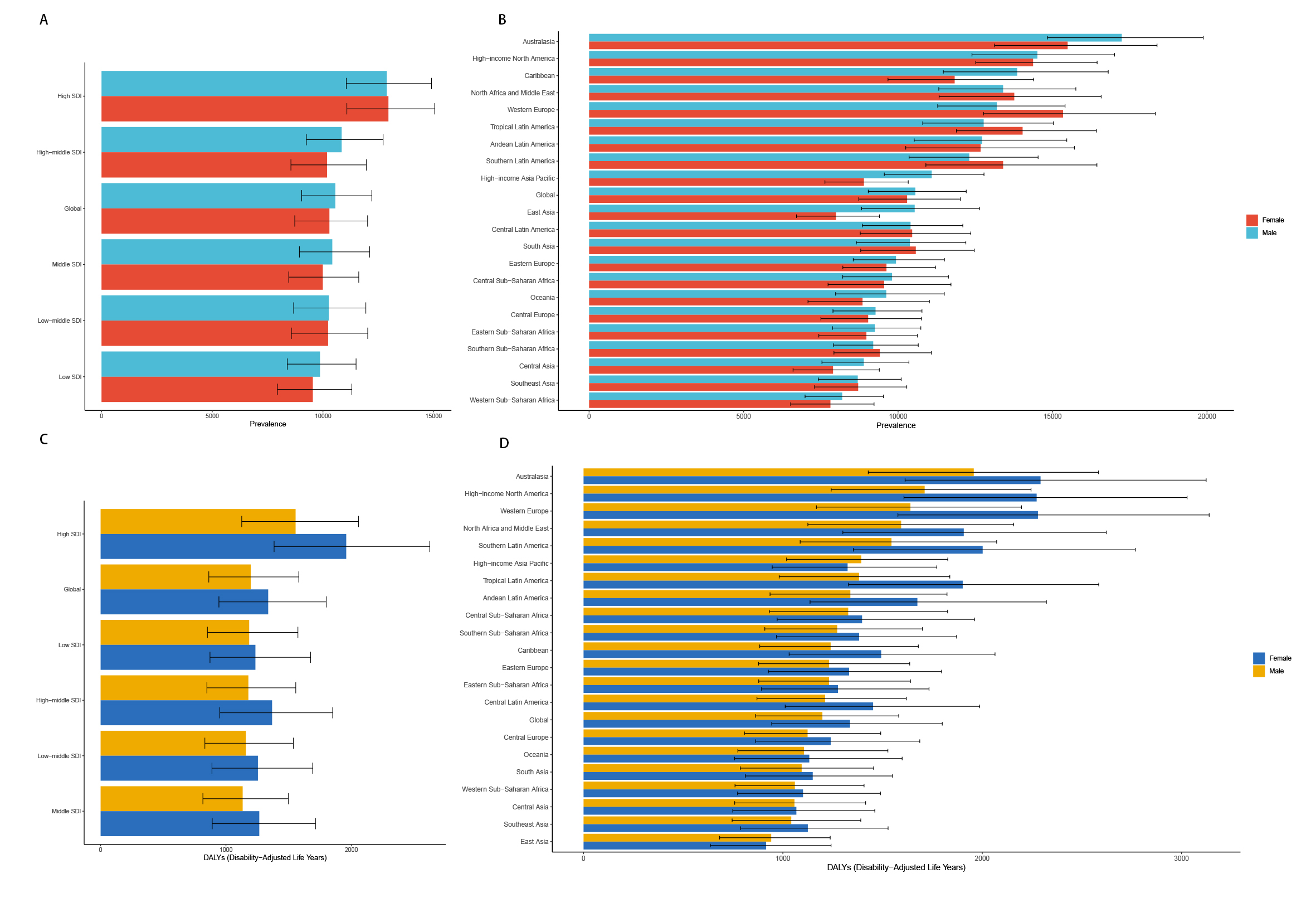


**Supplementary Figure 2.** Sex distribution of ASPR and ASDR for mental disorders among 5 SDI and 21 GBD regions in 2021. (A) ASPR of mental disorders among 5 SDI regions; (B) ASPR of mental disorders among 21 GBD regions; (C) ASDR of mental disorders among 5 SDI regions; (D) ASDR of mental disorders among 21 GBD regions. Abbreviation: ASPR, age-standardized prevalence rate; ASDR, age-standardized death rate.


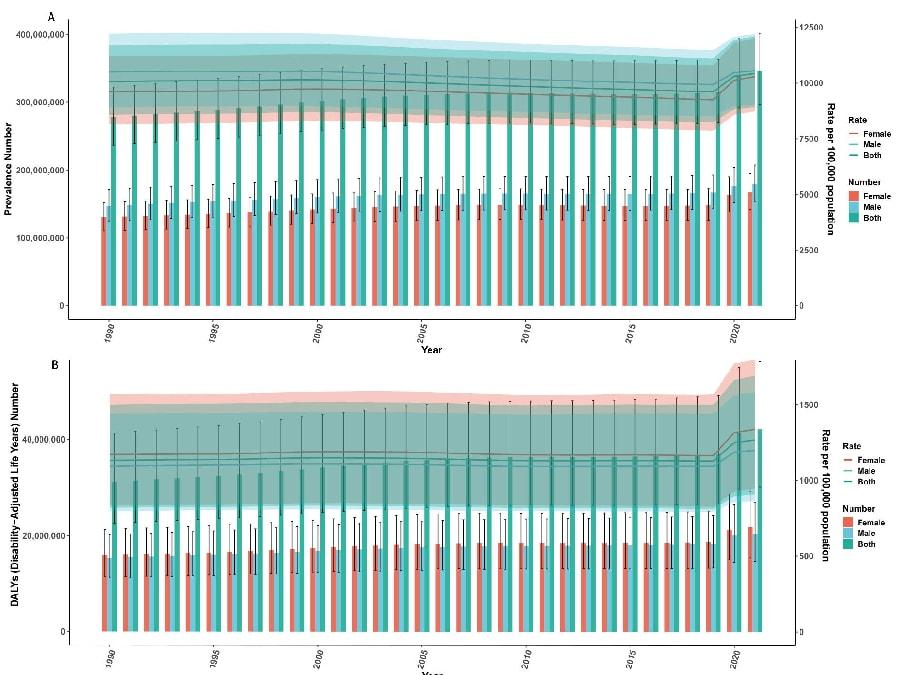


**Supplementary Figure 3.** Changes in prevalence/DALY rates by sex. (A) ASPR of mental disorders by sex; (B) ASDR of mental disorders by sex. Abbreviation: ASPR, age-standardized prevalence rate; ASDR, age-standardized death rate.


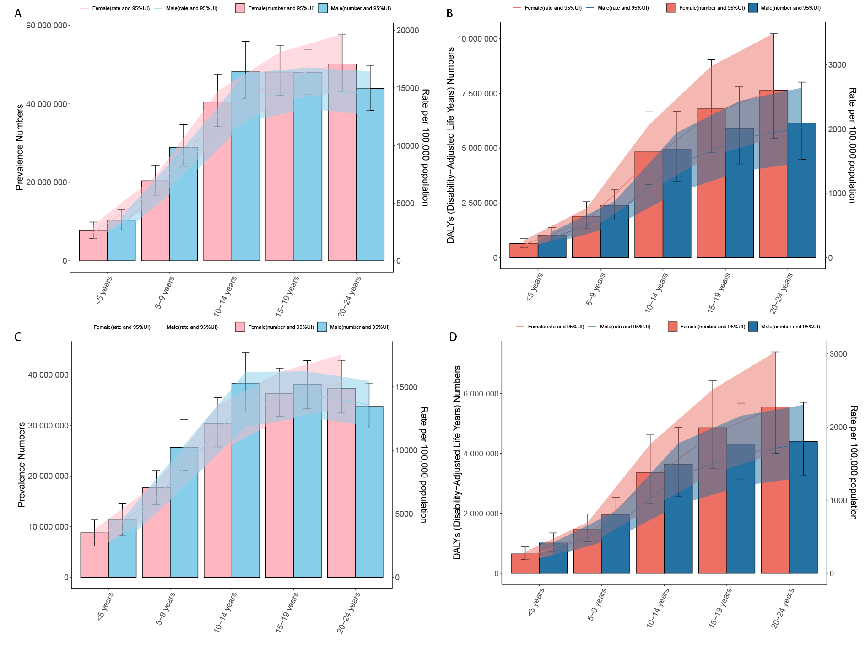


**Supplementary Figure 4.** Sex and age group analyses of the global burden of mental disorders in 1990 and 2021. (A) ASPR of mental disorders in 2021; (B) ASDR of mental disorders in 2021; (C) ASPR of mental disorders in 1990; (D) ASDR of mental disorders in 1990. Abbreviation: ASPR, age-standardized prevalence rate; ASDR, age-standardized death rate.


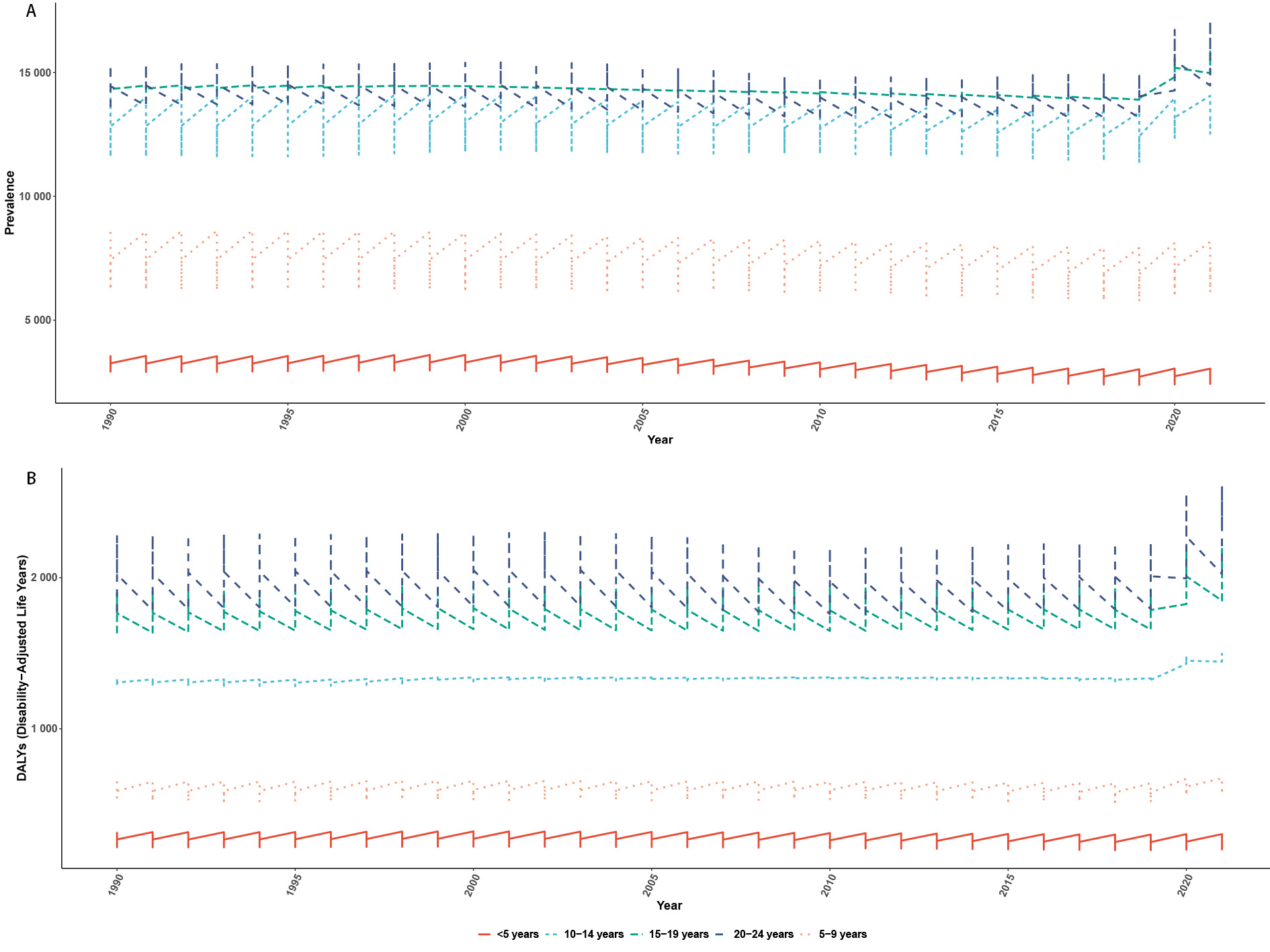


**Supplementary Figure 5.** Changes in prevalence/DALY rates by age. (A) ASPR of mental disorders among 5 age groups; (B) ASDR of mental disorders among 5 age groups. Abbreviation: ASPR, age-standardized prevalence rate; ASDR, age-standardized death rate.


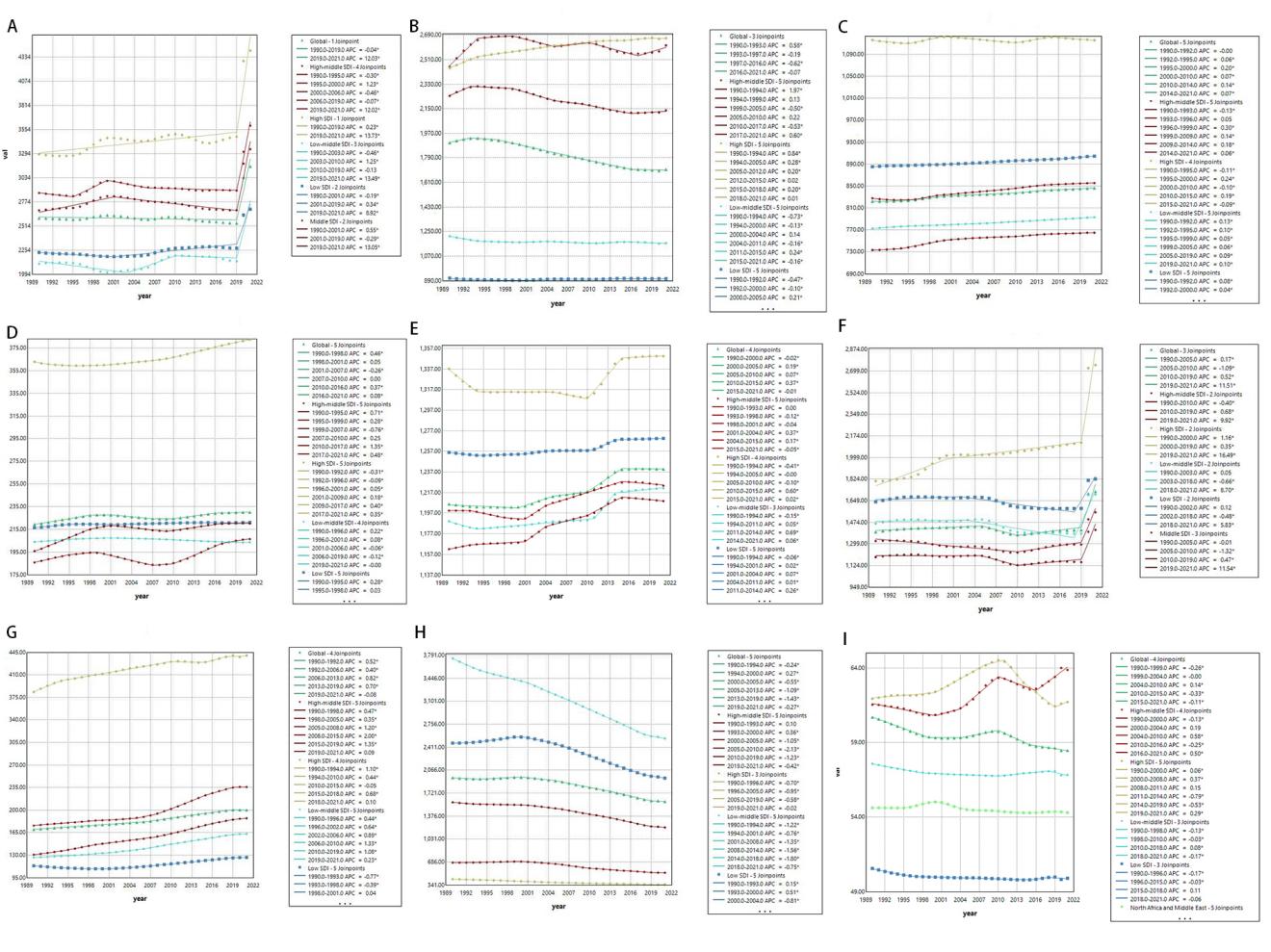


**Supplementary Figure 6.** Global and 5 SDI regions changes of age-standardized prevalence rates of 9 mental disorders from 1990 to 2021. (A) Anxiety disorders; (B) Attention-deficit/hyperactivity disorder; (C) Autism spectrum disorders; (D) Bipolar disorder; (E) Conduct disorder; (F) Depressive disorders; (G) Eating disorders; (H) Idiopathic developmental intellectual disability; (I) Schizophrenia. Abbreviation: APC, annual percentage change; AAPC, average annual percentage change; SDI, Socio-demographic Index.


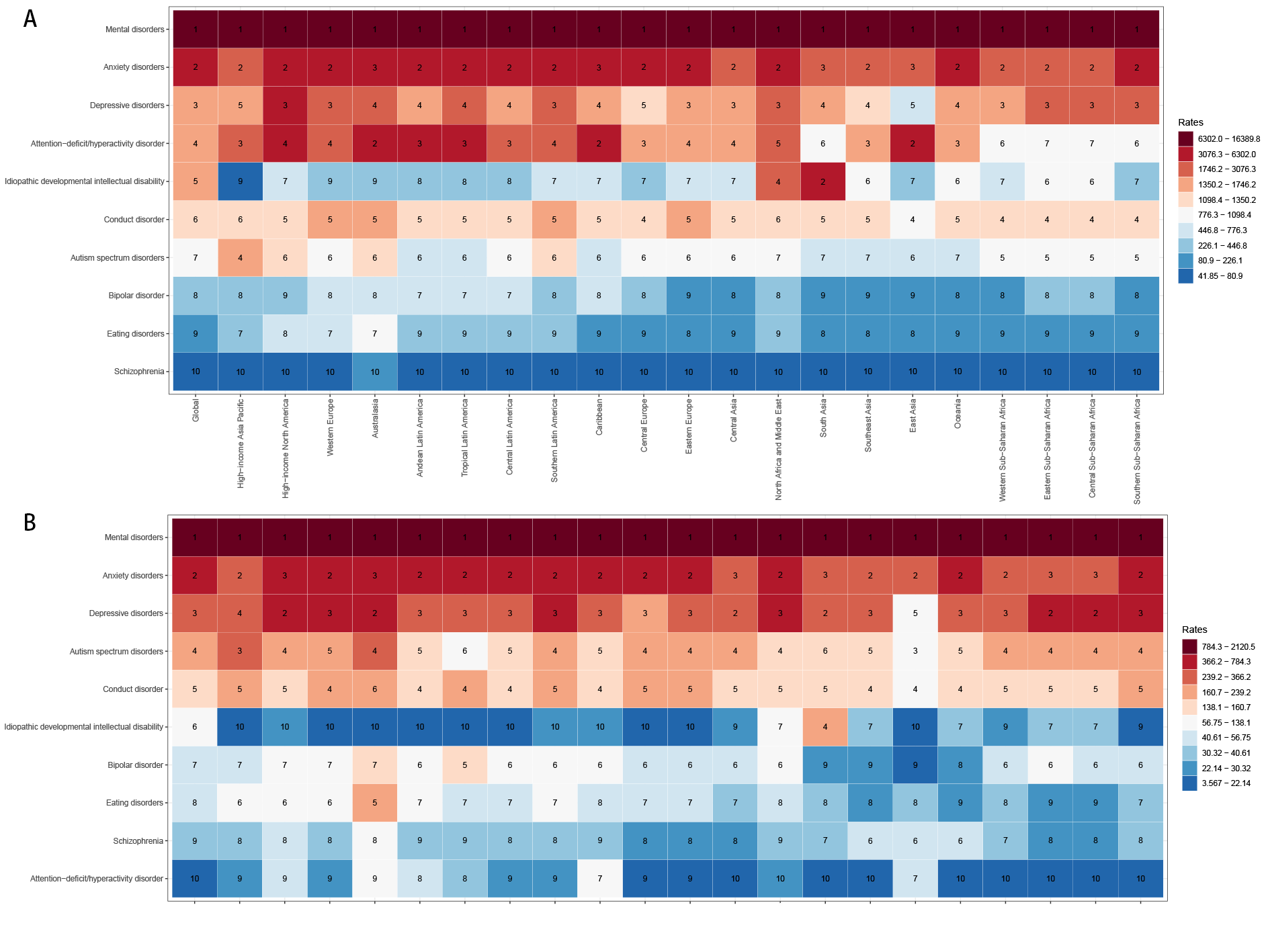


**Supplementary Figure 7.** Ranking of age-standardized prevalence and DALY rates for all mental disorders by location, 2021. (A) for prevalence (B) for DALY. The colors in the figure represent rankings from high (red) to low (blue), with the numbers representing the specific rankings of 11 types of CBDs globally, in various SDI regions, and 21 GBD regions. Abbreviation: SDI, sociodemographic index.

**
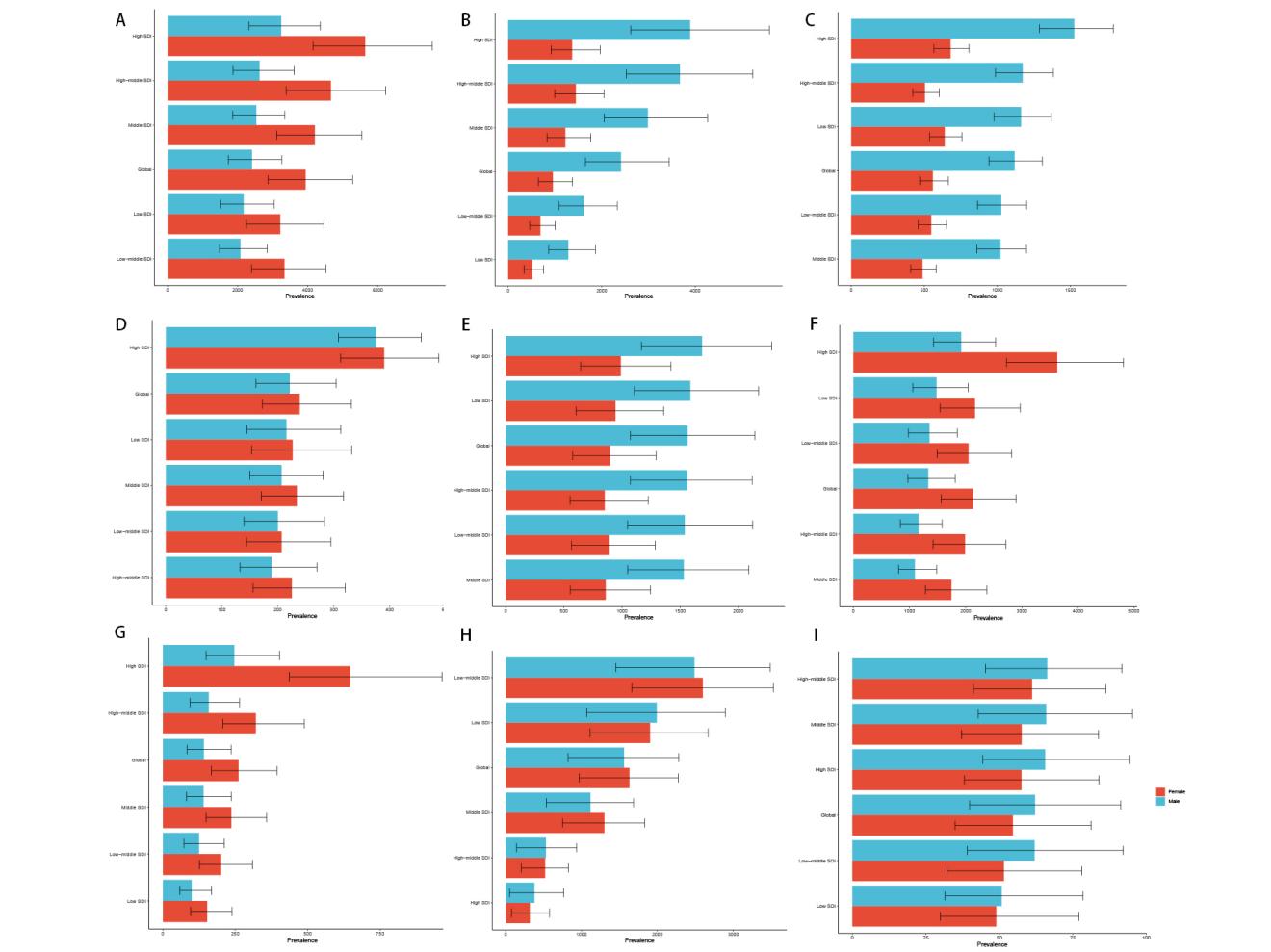
**

**Supplementary Figure 8.** Sex distribution of age-standardized prevalence rates for 9 mental disorder among 5 SDI regions in 2021. (A) Anxiety disorders; (B) Attention-deficit/ hyperactivity disorder; (C) Autism spectrum disorders; (D) Bipolar disorder; (E) Conduct disorder; (F) Depressive disorders; (G) Eating disorders; (H) Idiopathic developmental intellectual disability; (I) Schizophrenia.

**
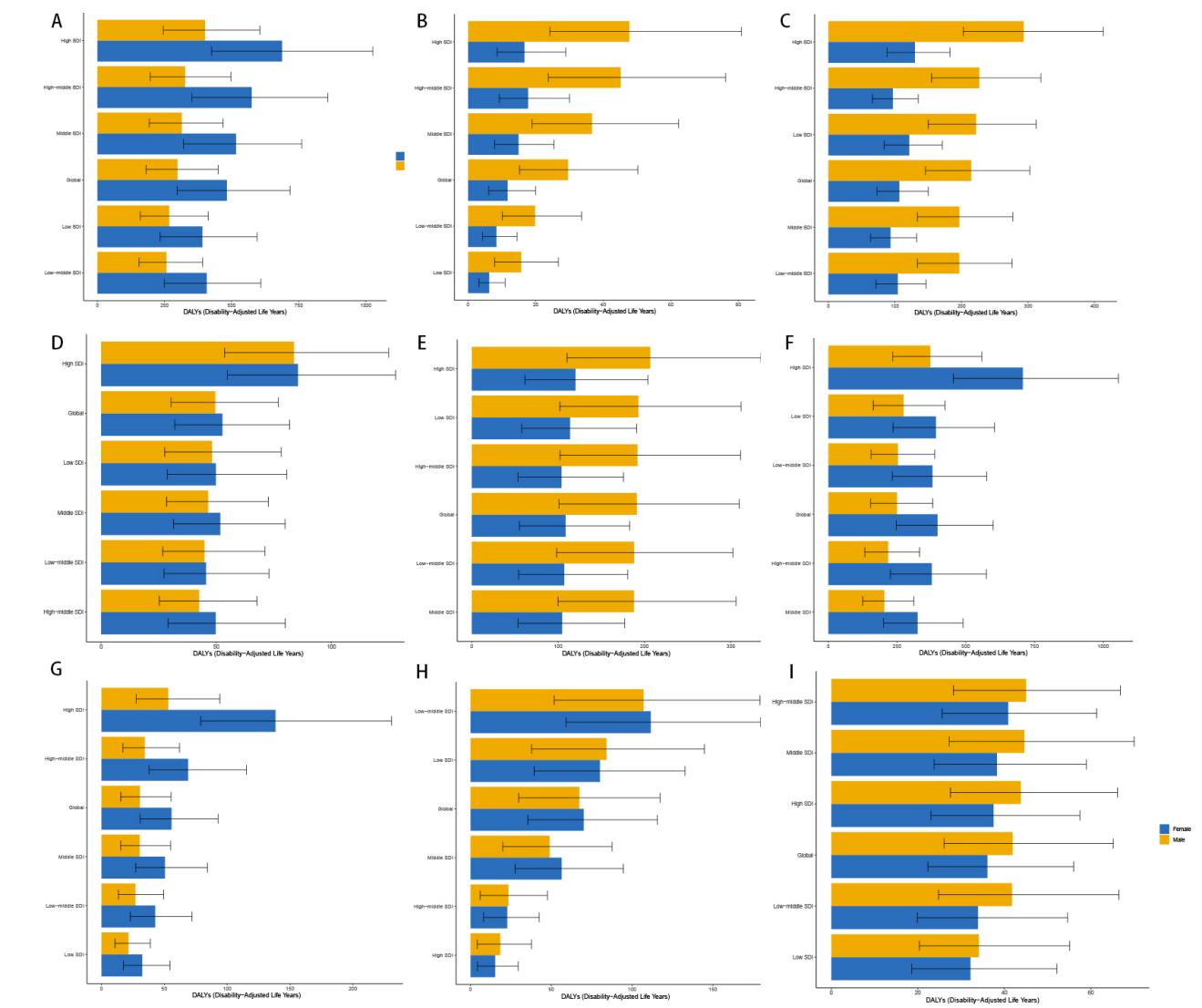
**

**Supplementary Figure 9.** Sex distribution of age-standardized DALY rates for 9 mental disorder among 5 SDI regions in 2021. (A) Anxiety disorders; (B) Attention-deficit/ hyperactivity disorder; (C) Autism spectrum disorders; (D) Bipolar disorder; (E) Conduct disorder; (F) Depressive disorders; (G) Eating disorders; (H) Idiopathic developmental intellectual disability; (I) Schizophrenia.
