# Supplementary material for: Global burden and trends of major mental disorders in individuals under 24 years of age from 1990 to 2021, with projections to 2050: insights from the Global Burden of Disease Study 2021
Source: Front Public Health. 2025 Sep 16;13:1635801. doi: 10.3389/fpubh.2025.1635801 (PMC12481897; doi:10.3389/fpubh.2025.1635801)
Supplement: Supplementary file 1 [file Presentation_1.zip › Supplementary Figure 10-14.DOCX]

Supplementary Figures 2

**
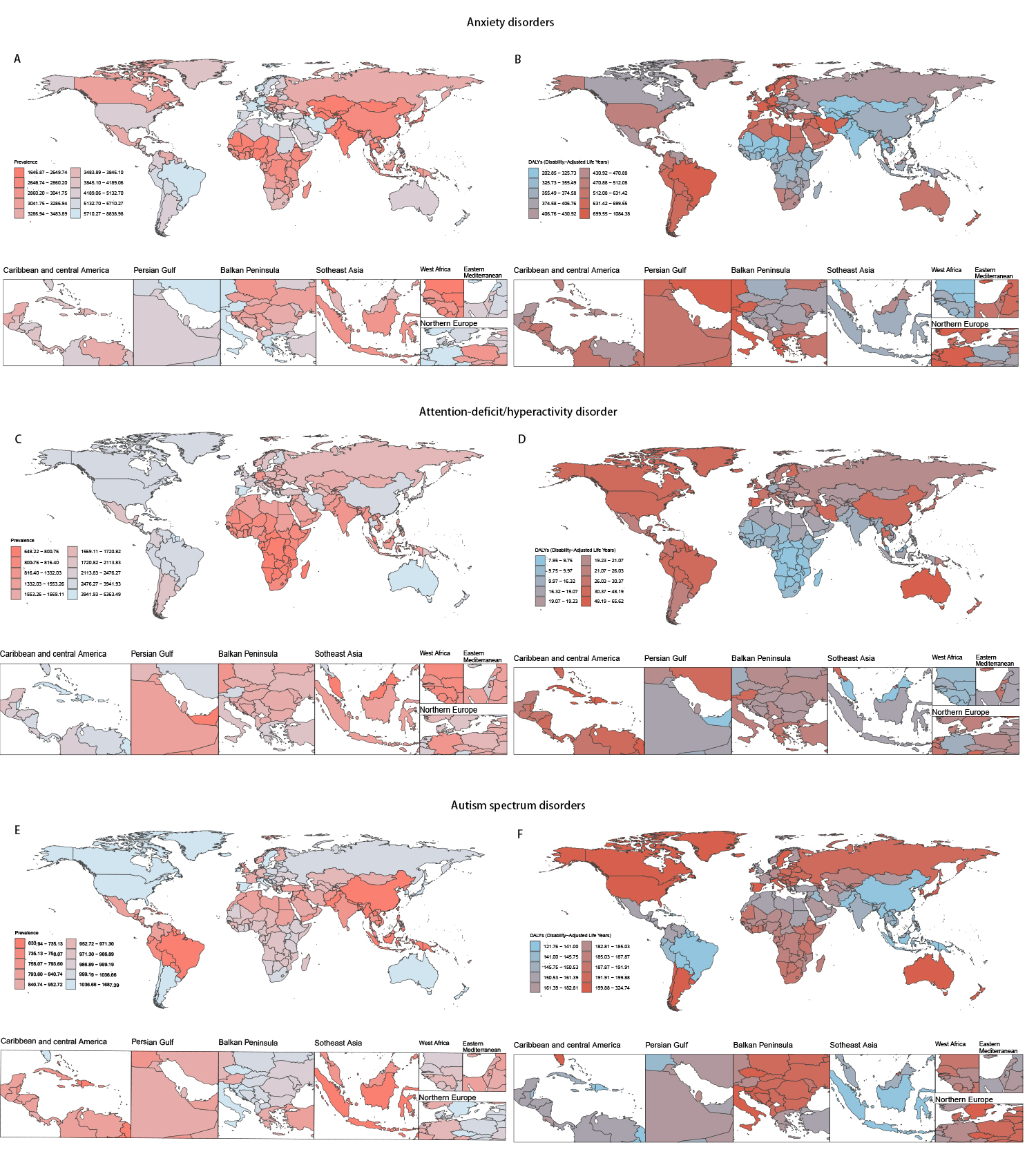
**

**Supplementary Figure 10.** Global burden of anxiety disorders, ADHD, and ASD in 2021. (A) age-standardized prevalence rates for anxiety disorders; (B) age-standardized DALY rates for anxiety disorders; (C) age-standardized prevalence rate for ADHD; (D) age-standardized DALY rate for ADHD; (E) age-standardized prevalence for ASD; (F) age-standardized DALY rate for ASD in 2021. Abbreviation: ADHD, attention-deficit hyperactivity disorder; ASD, autism spectrum disorders; IDII, idiopathic developmental intellectual disability.

**
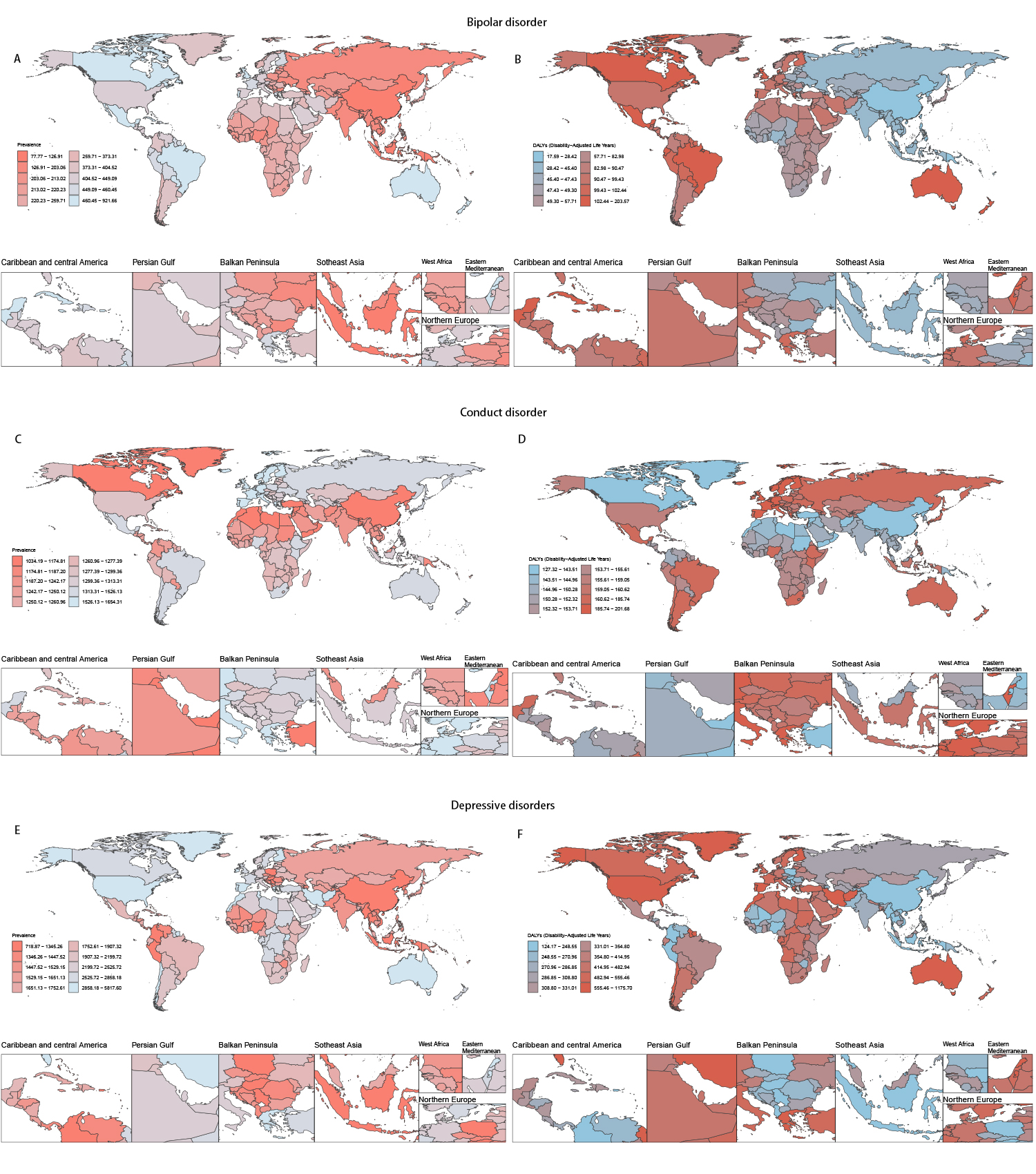
**

**Supplementary Figure 11**. Global burden of bipolar disorders, conduct disorder, and depressive disorders in 2021. (A) age-standardized prevalence rates for bipolar disorder; (B) age-standardized DALY rates for bipolar disorder; (C) age-standardized prevalence rate for conduct disorder; (D) age-standardized DALY rate for conduct disorder; (E) age-standardized prevalence for depressive disorders; (F) age-standardized DALY rate for depressive disorders in 2021.

**
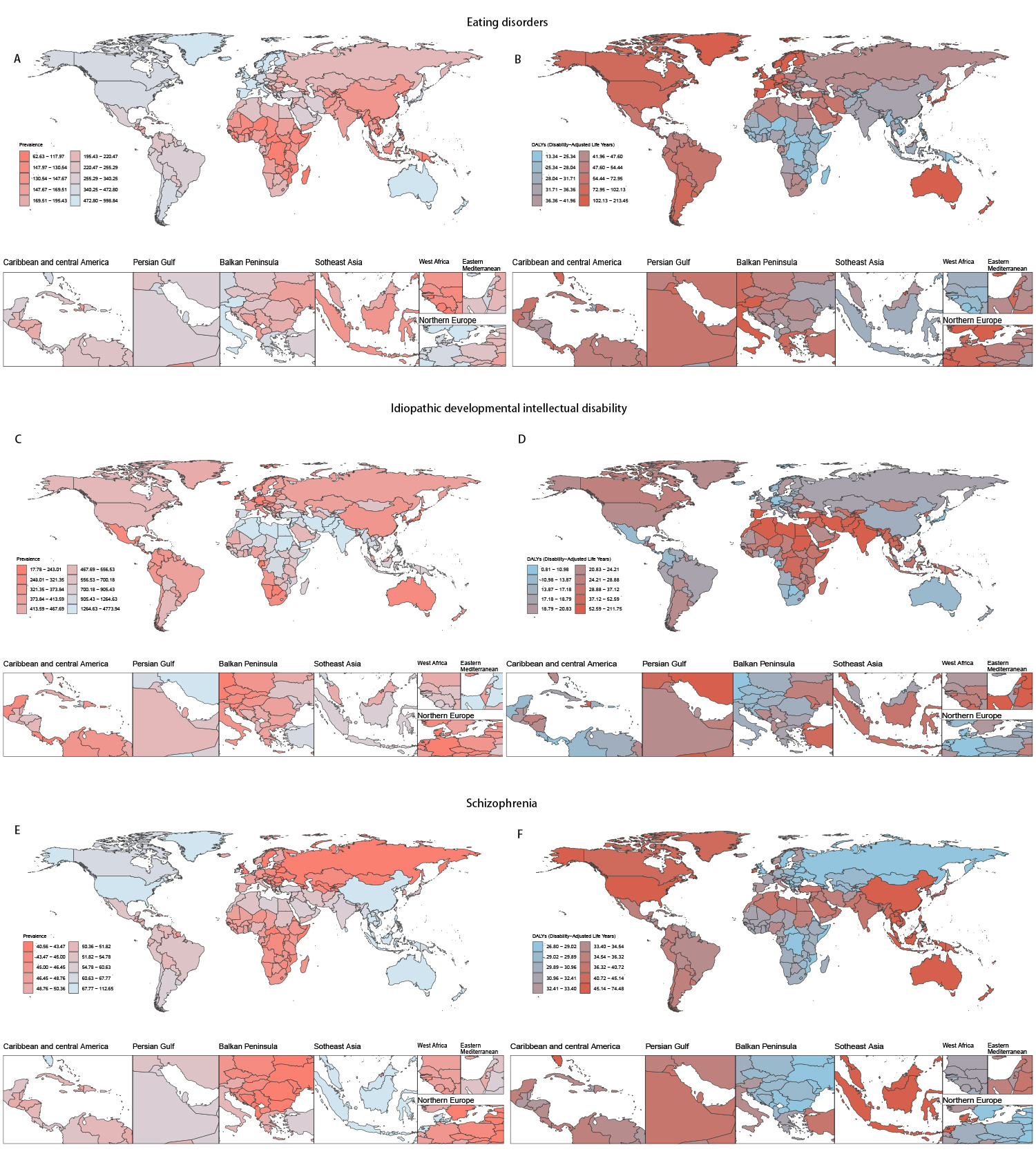
**

**Supplementary Figure 12**. Global burden of eating disorders, IDII, and schizophrenia in 2021. (A) age-standardized prevalence rates for eating disorders; (B) age-standardized DALY rates for eating disorders; (C) age-standardized prevalence rate for IDII; (D) age-standardized DALY rate for IDII; (E) age-standardized prevalence for schizophrenia; (F) age-standardized DALY rate for schizophrenia in 2021. Abbreviation: IDII, idiopathic developmental intellectual disability.


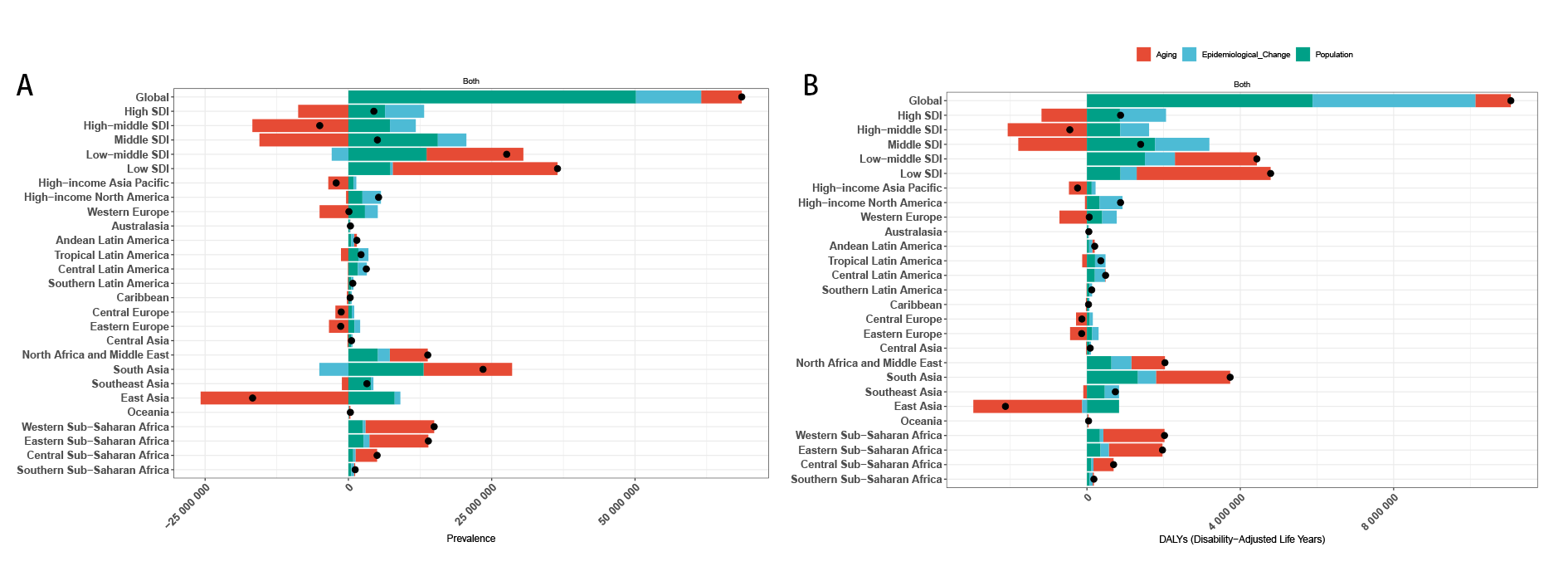


**Supplementary Figure 13**. Results of decomposition analysis of mental disorders. (A) Age-standardized prevalence rates; (B) Age-standardized DALY rates.

**

**

**Supplementary Figure 14.** The correlation between the ASDR of mental disorders and SDI in (A) 21 GBD regions and (B) 204 countries and territories. Abbreviation: ASDR, the age-standardized DALY rates; DALYs, the disability-adjusted life years; SDI, Socio-Demographic Index.
