# Supplementary material for: Global burden and trends of major mental disorders in individuals under 24 years of age from 1990 to 2021, with projections to 2050: insights from the Global Burden of Disease Study 2021
Source: Front Public Health. 2025 Sep 16;13:1635801. doi: 10.3389/fpubh.2025.1635801 (PMC12481897; doi:10.3389/fpubh.2025.1635801)
Supplement: Supplementary file 1 [file Presentation_1.zip › Supplementary Figure 15-20.DOCX]

Supplementary Figures 3


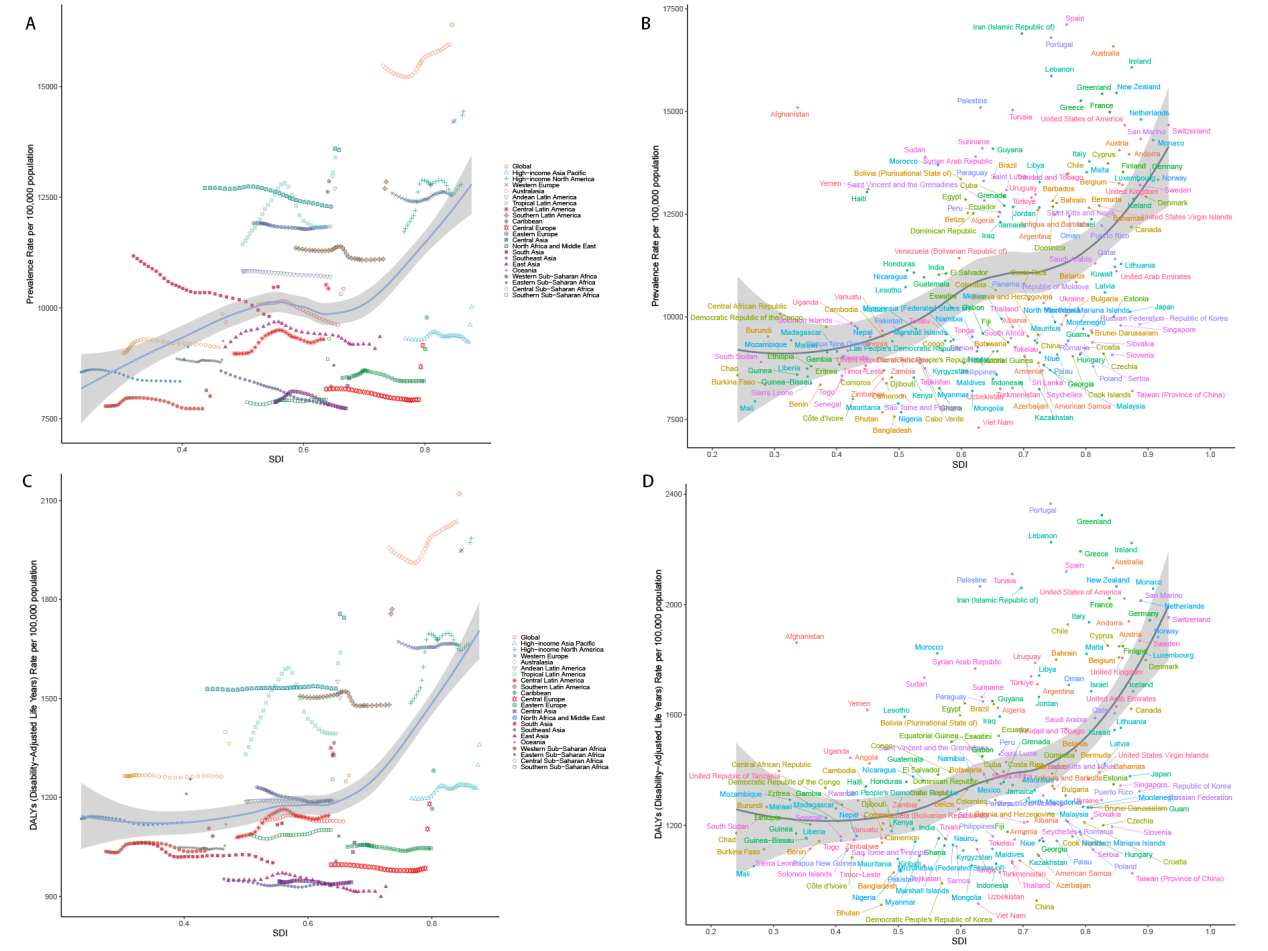


**Supplementary Figure 15**. Change in prevalence of 9 mental disorders decomposed by three population-level determinants: aging, population and epidemiological change at the global level and various regions. (A) Anxiety disorders; (B) Attention-deficit/hyperactivity disorder; (C) Autism spectrum disorders; (D) Bipolar disorder; (E) Conduct disorder; (F) Depressive disorders; (G) Eating disorders; (H) Idiopathic developmental intellectual disability; (I) Schizophrenia. The black dots indicate the total value of change attributable to all three components. Abbreviation: SDI, Socio-demographic Index.

**
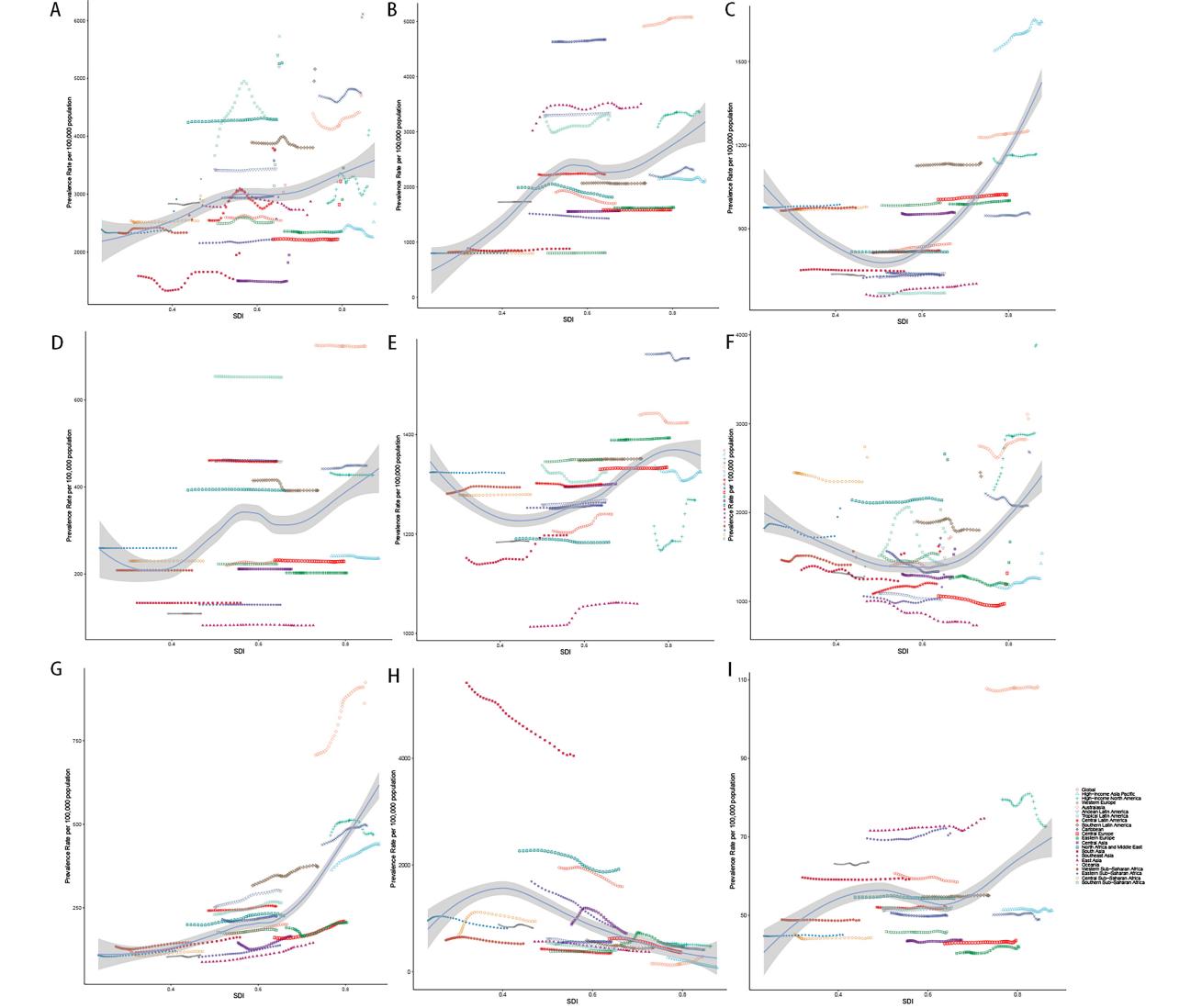
Supplementary Figure 16**. The correlation between age-standardized prevalence rates of mental disorder subtypes and SDI in 21 GBD regions. (A) Anxiety disorders; (B) Attention-deficit/hyperactivity disorder; (C) Autism spectrum disorders; (D) Bipolar disorder; (E) Conduct disorder; (F) Depressive disorders; (G) Eating disorders; (H) Idiopathic developmental intellectual disability; (I) Schizophrenia. Abbreviation: SDI, Socio-Demographic Index.

**
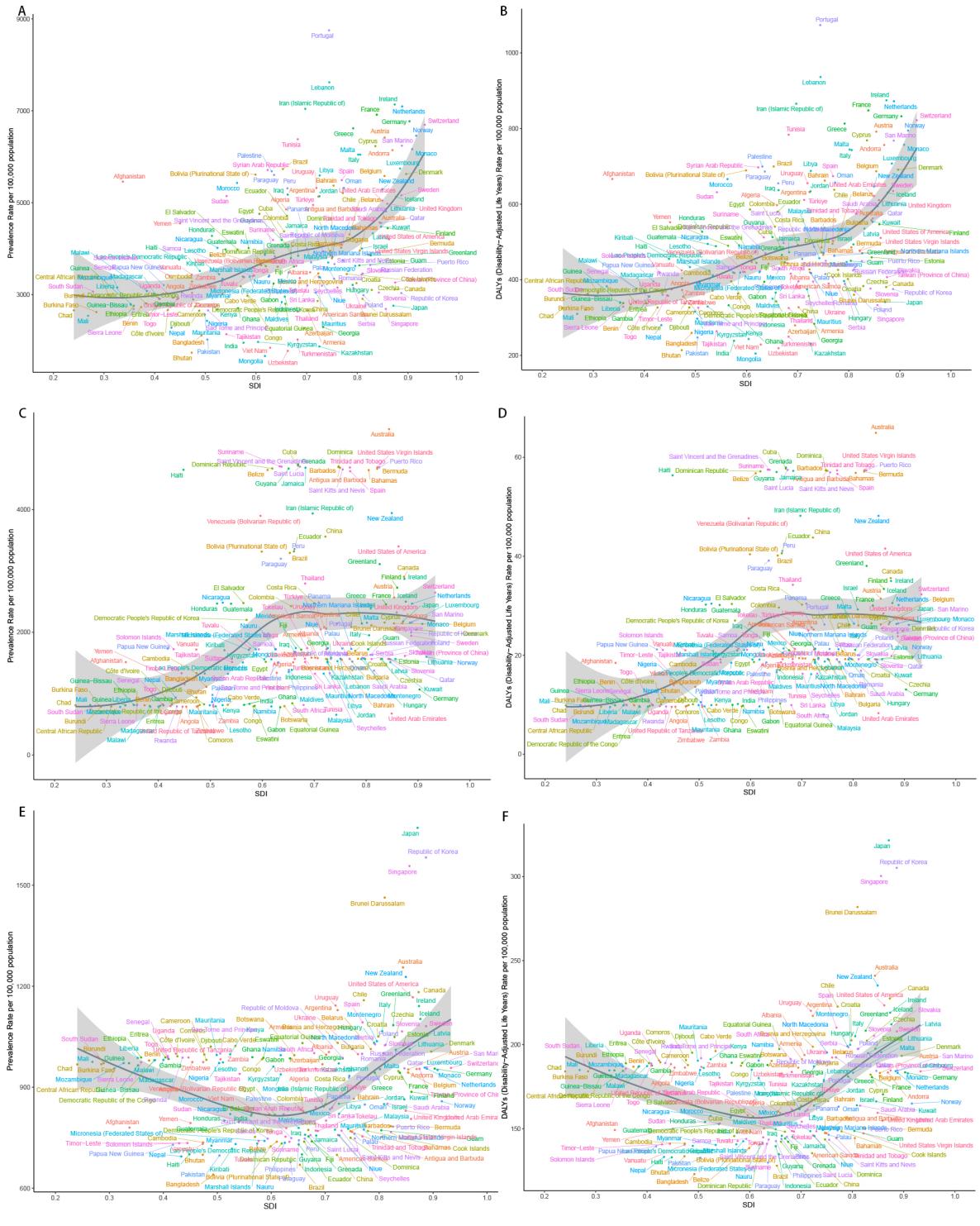
**

**Supplementary Figure 17**. The correlation between burden of anxiety disorders, ADHD, and ASD and SDI in 204 countries and territories. (A) age-standardized prevalence rates for anxiety disorders; (B) age-standardized DALY rates for anxiety disorders; (C) age-standardized prevalence rate for ADHD; (D) age-standardized DALY rate for ADHD; (E) age-standardized prevalence for ASD; (F) age-standardized DALY rate for ASD in 2021. Abbreviation: ADHD, attention-deficit hyperactivity disorder; ASD, autism spectrum disorders; IDII, idiopathic developmental intellectual disability.

**
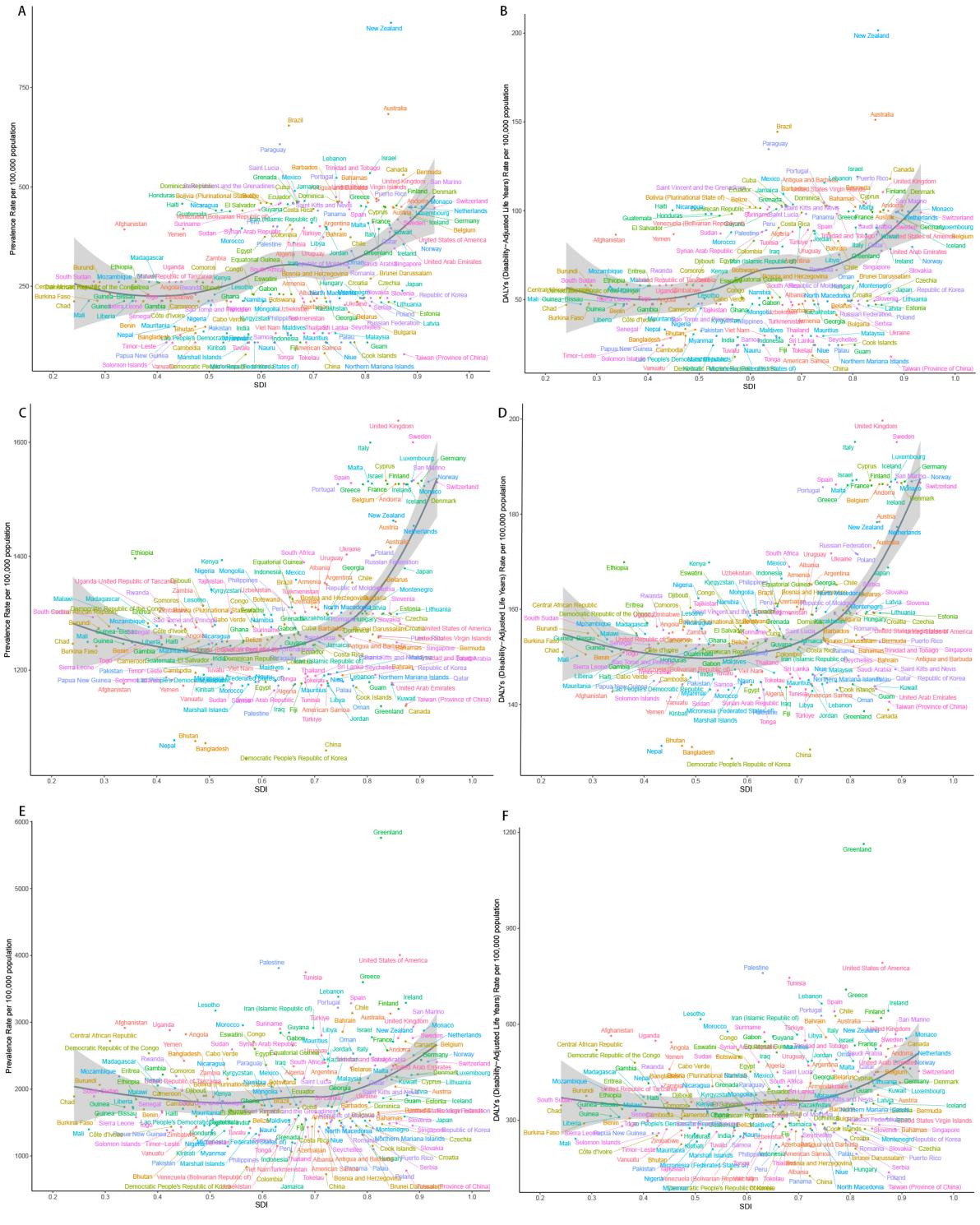
**

**Supplementary Figure 18**. The correlation between burden of bipolar disorders, conduct disorder, and depressive disorders and SDI in 204 countries and territories. (A) age-standardized prevalence rates for bipolar disorder; (B) age-standardized DALY rates for bipolar disorder; (C) age-standardized prevalence rate for conduct disorder; (D) age-standardized DALY rate for conduct disorder; (E) age-standardized prevalence for depressive disorders; (F) age-standardized DALY rate for depressive disorders in 2021.

**
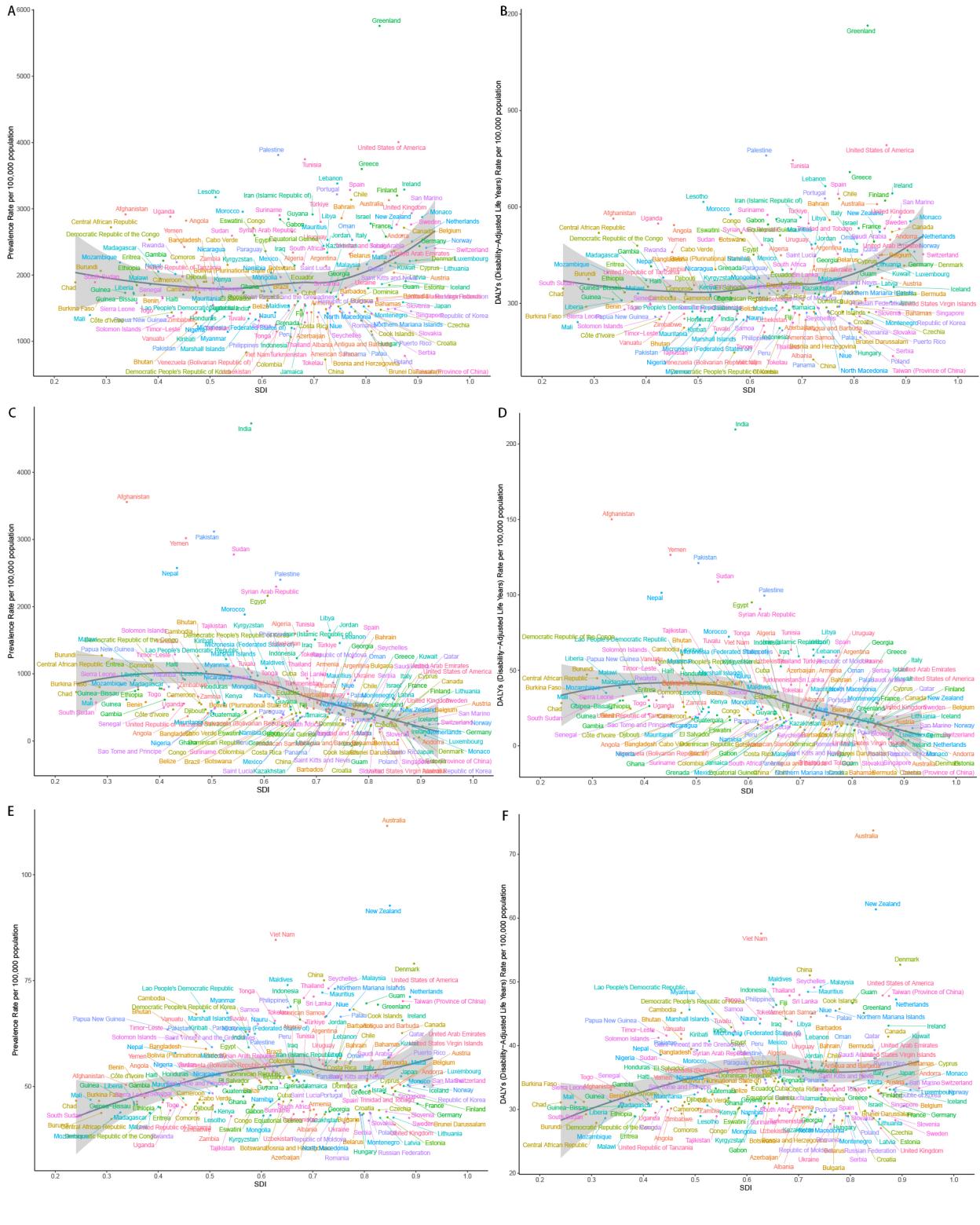
**

**Supplementary Figure 19**. The correlation between burden of eating disorders, IDII, and schizophrenia and SDI in 204 countries and territories. (A) age-standardized prevalence rates for eating disorders; (B) age-standardized DALY rates for eating disorders; (C) age-standardized prevalence rate for IDII; (D) age-standardized DALY rate for IDII; (E) age-standardized prevalence for schizophrenia; (F) age-standardized DALY rate for schizophrenia in 2021. Abbreviation: IDII, idiopathic developmental intellectual disability.


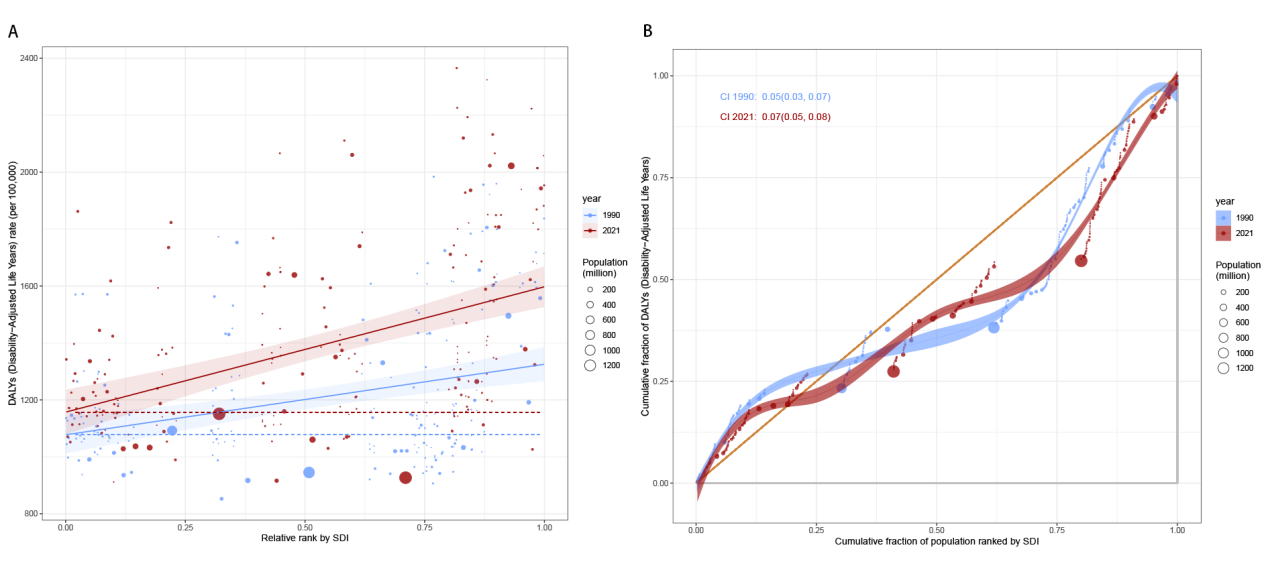


**Supplementary Figure 20.** Results of inequalities analysis for the ASDR of mental disorders. (A) Health inequality regression curves of mental disorders. (B) Health inequality concentration curves of mental disorders. Abbreviation: ASDR, the age-standardized DALY rates; DALYs, the disability-adjusted life years; SDI, Socio-Demographic Index.
