# Supplementary material for: Global burden and trends of major mental disorders in individuals under 24 years of age from 1990 to 2021, with projections to 2050: insights from the Global Burden of Disease Study 2021
Source: Front Public Health. 2025 Sep 16;13:1635801. doi: 10.3389/fpubh.2025.1635801 (PMC12481897; doi:10.3389/fpubh.2025.1635801)
Supplement: Supplementary file 1 [file Presentation_1.zip › Supplementary Figure 23.DOCX]

Supplementary Figures 6


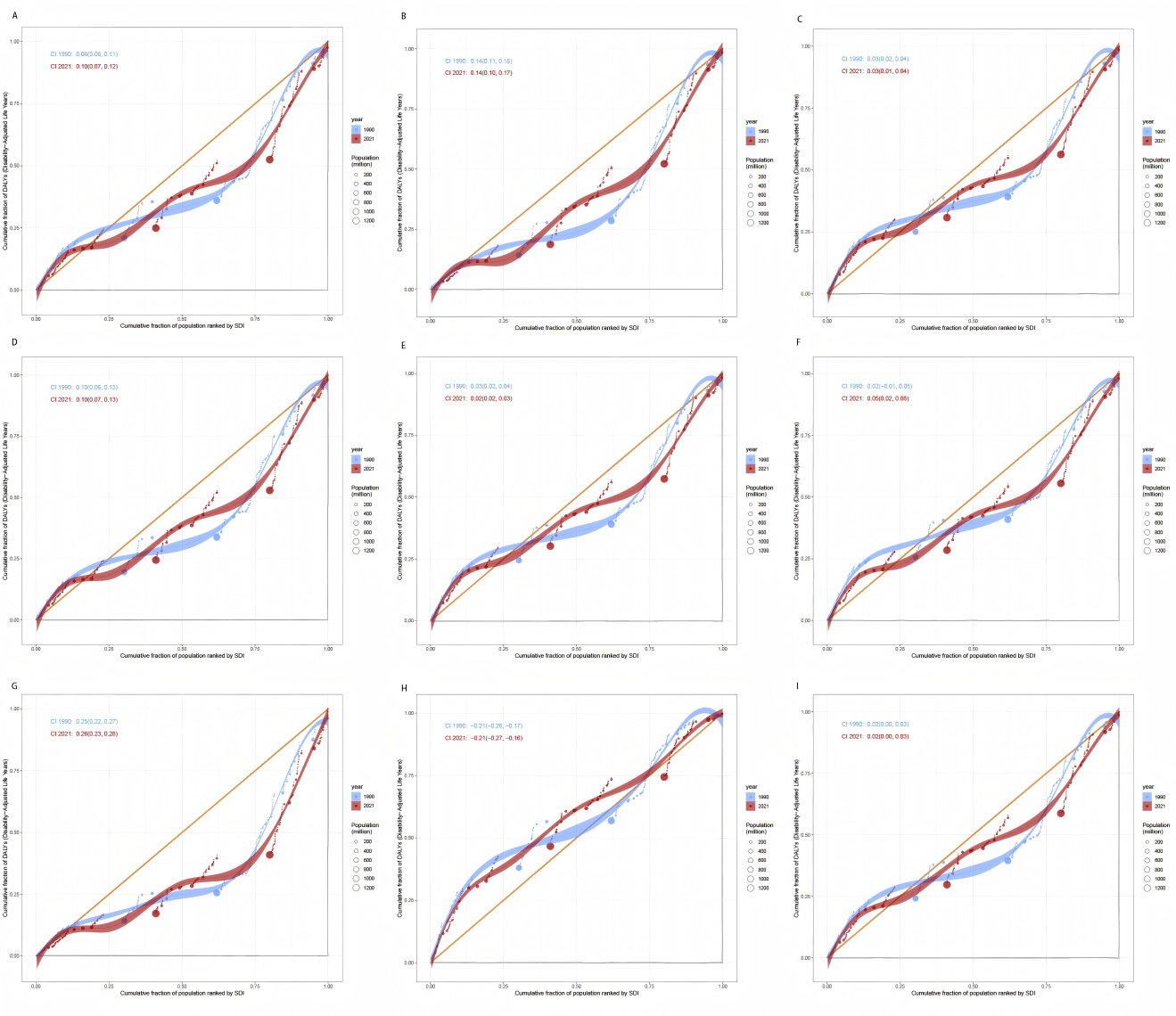


**Supplementary Figure 23**. Health inequality concentration curves for the DALY of 9 mental disorders. (A) Anxiety disorders; (B) Attention-deficit/hyperactivity disorder; (C) Autism spectrum disorders; (D) Bipolar disorder; (E) Conduct disorder; (F) Depressive disorders; (G) Eating disorders; (H) Idiopathic developmental intellectual disability; (I) Schizophrenia.
