# Supplementary material for: Global burden and trends of major mental disorders in individuals under 24 years of age from 1990 to 2021, with projections to 2050: insights from the Global Burden of Disease Study 2021
Source: Front Public Health. 2025 Sep 16;13:1635801. doi: 10.3389/fpubh.2025.1635801 (PMC12481897; doi:10.3389/fpubh.2025.1635801)
Supplement: Supplementary file 1 [file Presentation_1.zip › Supplementary Figure 24-29.DOCX]

Supplementary Figures 6


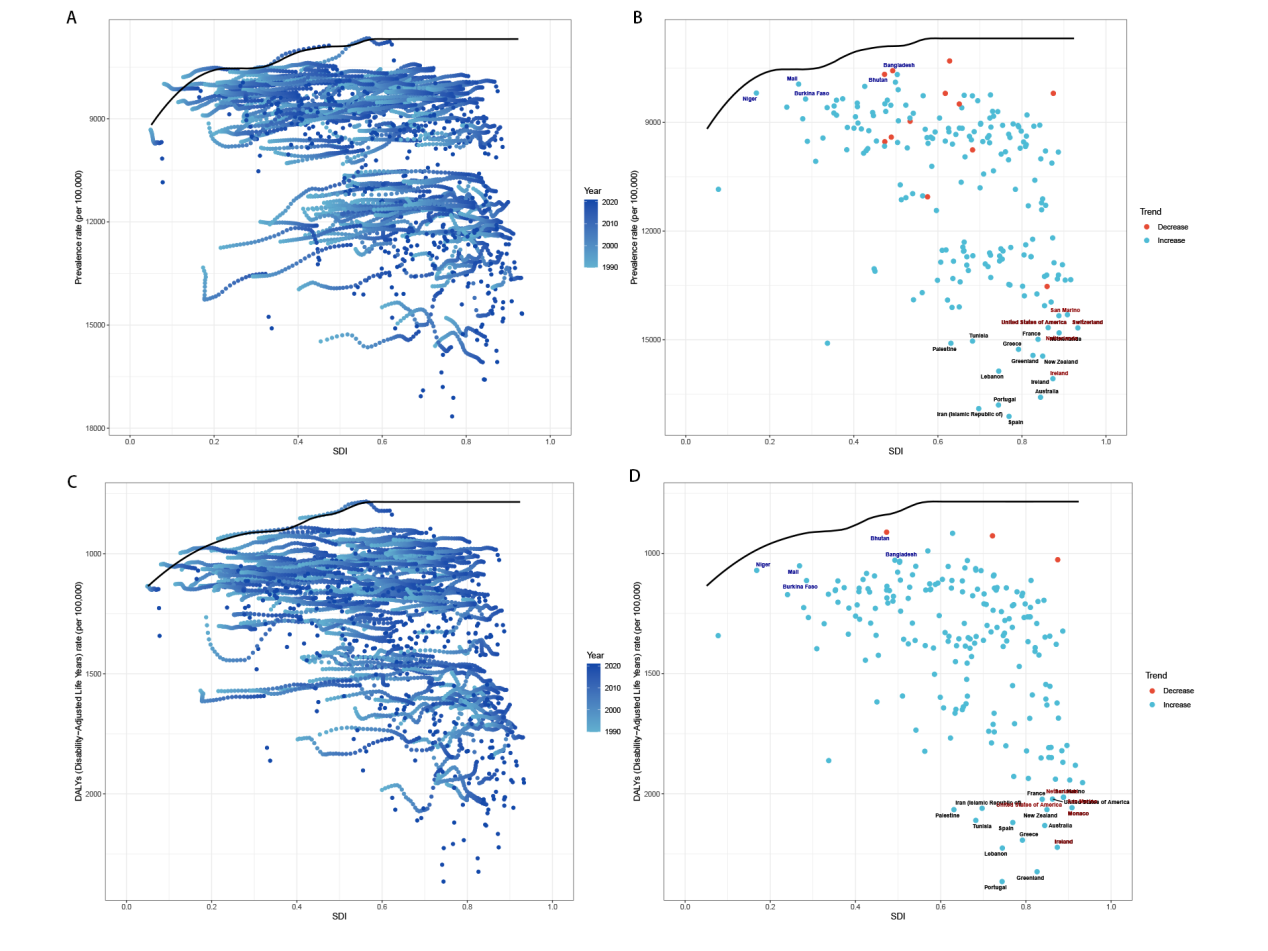


**Supplementary Figure 24.** The relationship between SDI and burden for mental disorders. (A) The color change from light blue (1990) to dark bule (2021) represents the change in years. (B) Each point represents a specific country or territory in 2021, the frontier line is shown in black. Abbreviation: ASDR, the age-standardized DALY rates; DALYs, the disability-adjusted life years; SDI, Socio-Demographic Index.

**
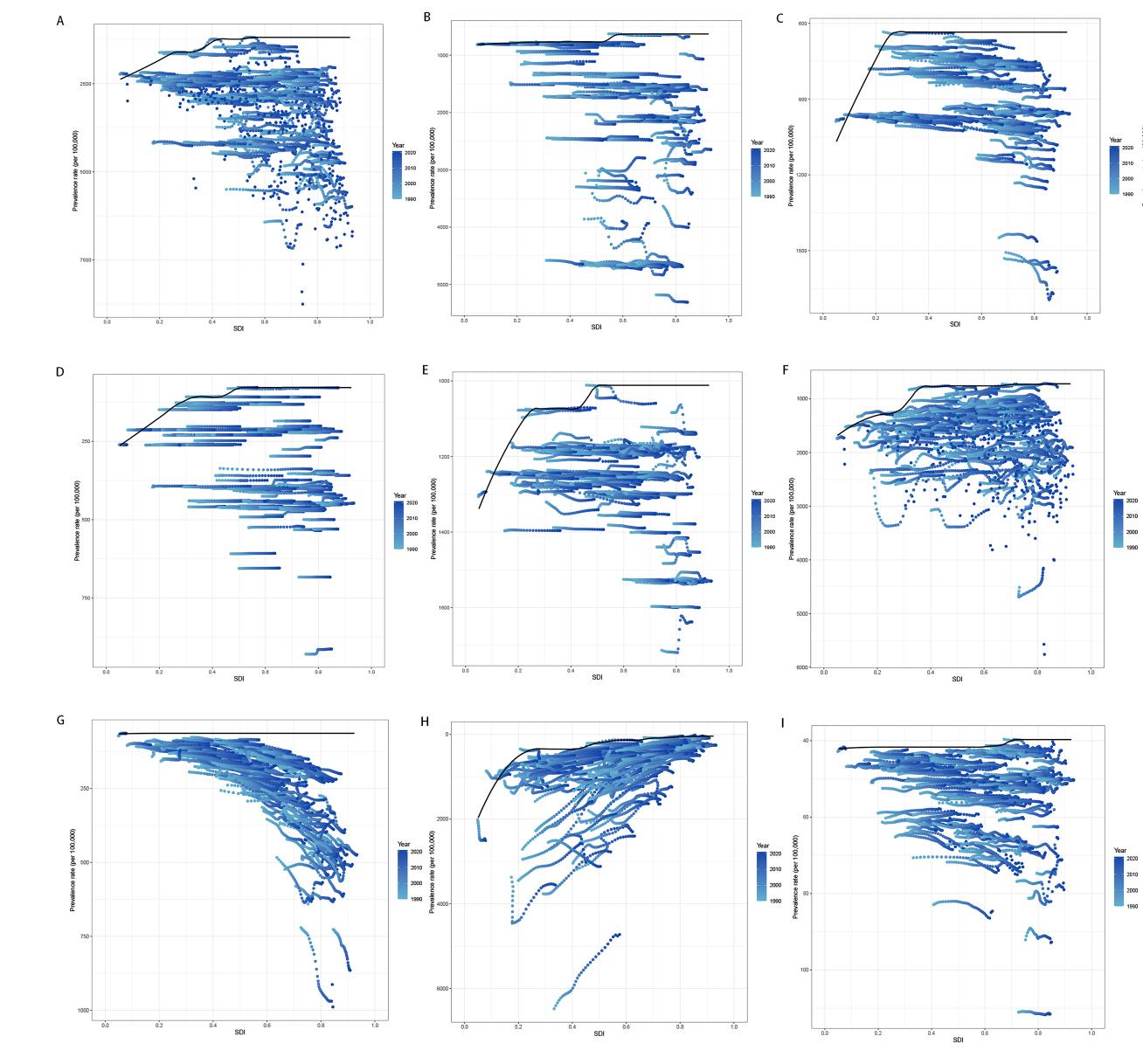
Supplementary Figure 25**. Frontier analysis for the age-standardized prevalence rates change in years of 9 mental disorders from 1990 to 2021. (A) Anxiety disorders; (B) Attention-deficit/hyperactivity disorder; (C) Autism spectrum disorders; (D) Bipolar disorder; (E) Conduct disorder; (F) Depressive disorders; (G) Eating disorders; (H) Idiopathic developmental intellectual disability; (I) Schizophrenia.

**
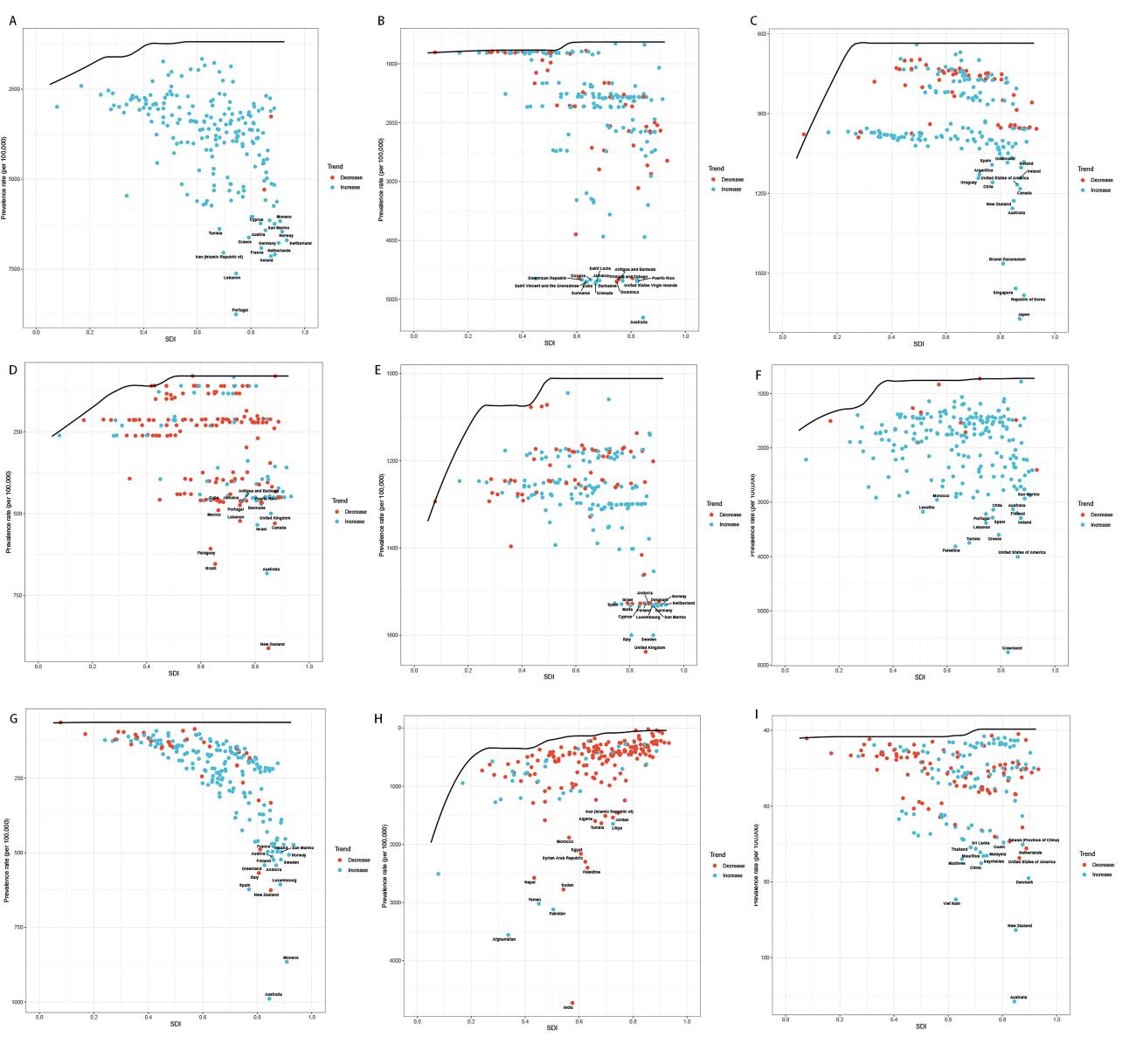
Supplementary Figure 26.** The relationship between SDI and age-standardized prevalence rates for 9 mental disorders, each point represents a specific country or territory in 2021. (A) Anxiety disorders; (B) Attention-deficit/hyperactivity disorder; (C) Autism spectrum disorders; (D) Bipolar disorder; (E) Conduct disorder; (F) Depressive disorders; (G) Eating disorders; (H) Idiopathic developmental intellectual disability; (I) Schizophrenia.

**
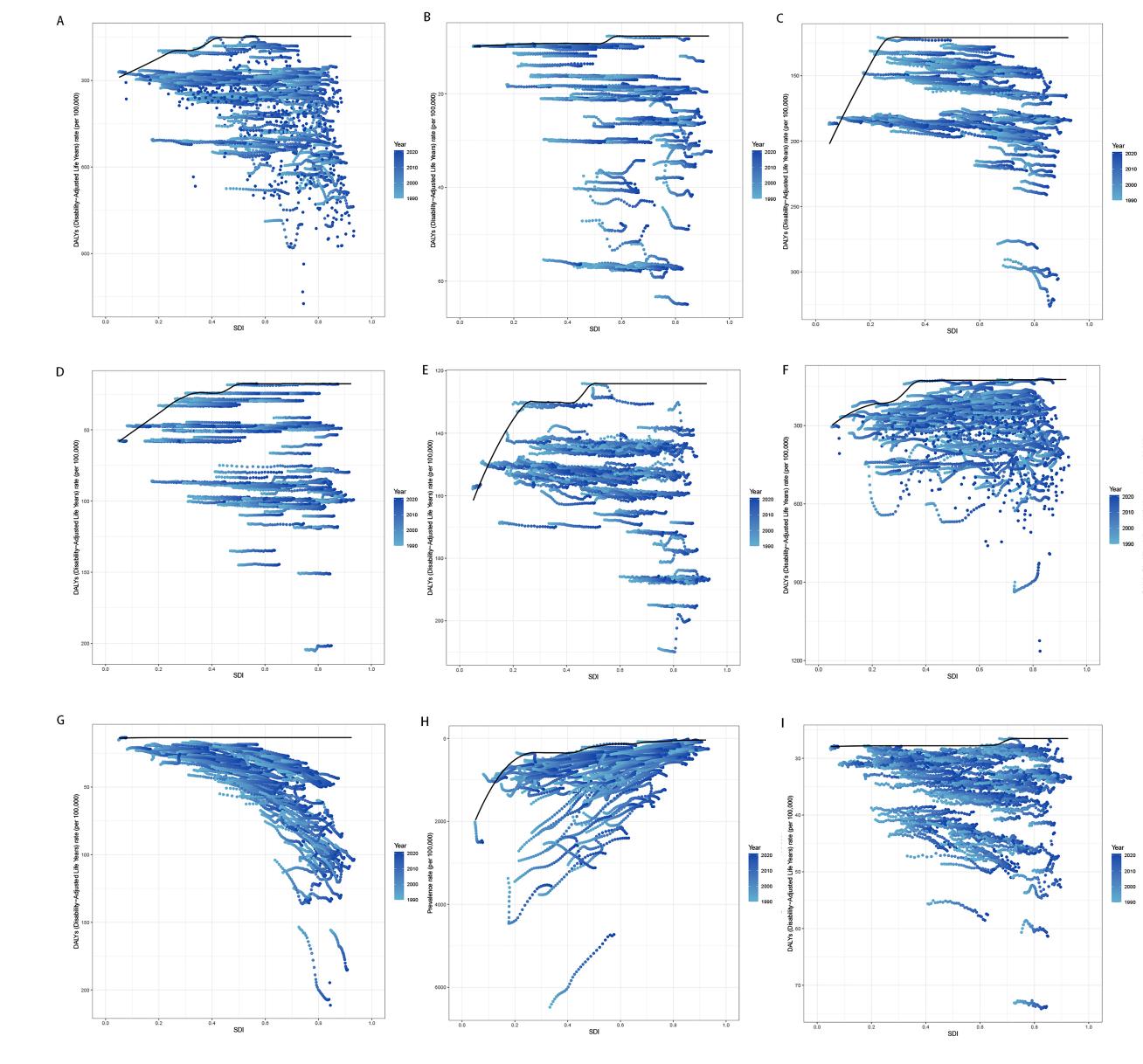
Supplementary Figure 27**. Frontier analysis for the age-standardized DALY rates change in years of 9 mental disorders from 1990 to 2021. (A) Anxiety disorders; (B) Attention-deficit/hyperactivity disorder; (C) Autism spectrum disorders; (D) Bipolar disorder; (E) Conduct disorder; (F) Depressive disorders; (G) Eating disorders; (H) Idiopathic developmental intellectual disability; (I) Schizophrenia.

**
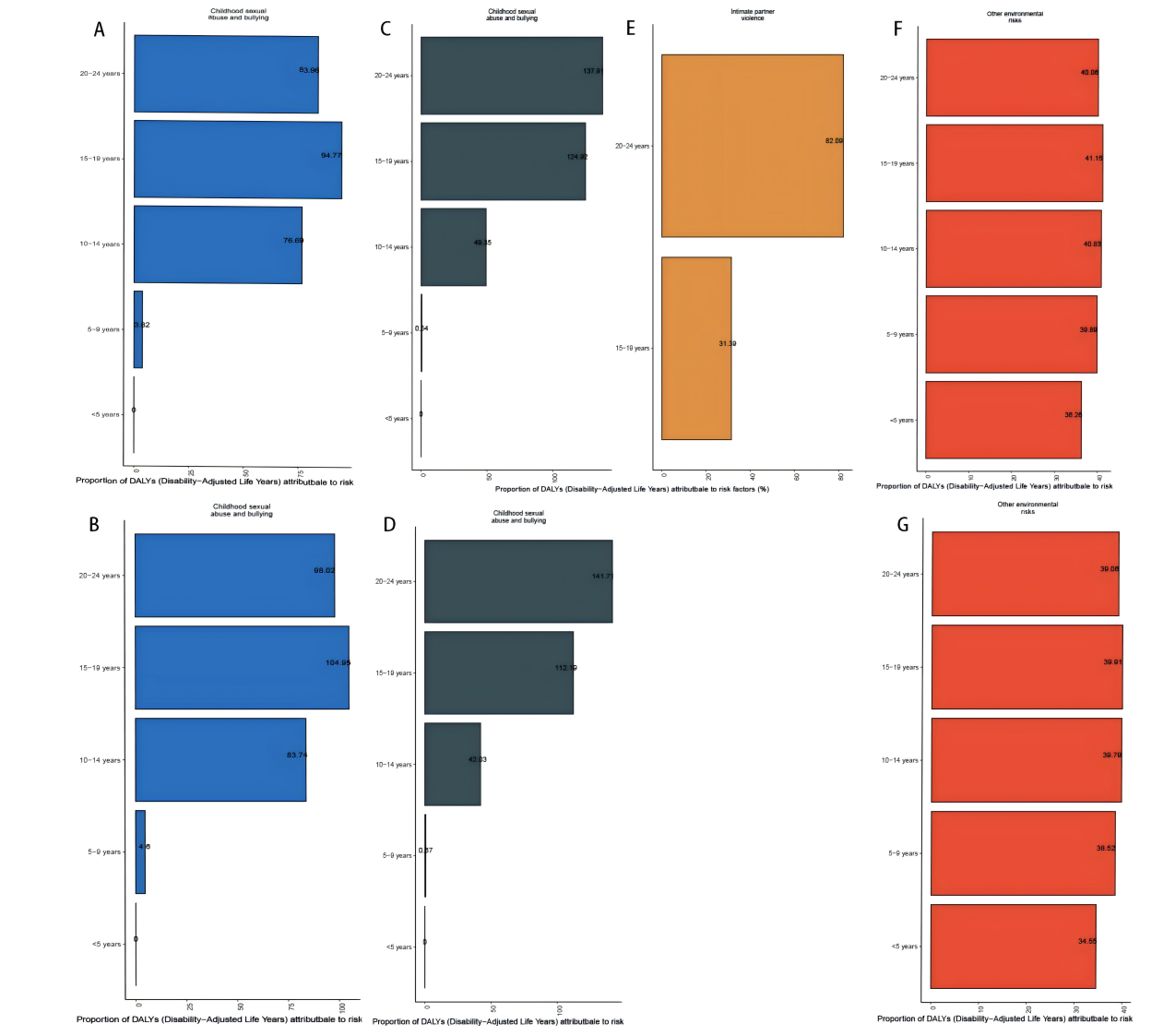
**

**Supplementary Figure 28**. The proportion of age-standardized DALY rates for mental disorders attributed to risk factors across age groups, female and male. (A) Anxiety disorders; (B and C) Depressive disorders; (D) Idiopathic developmental intellectual disability.


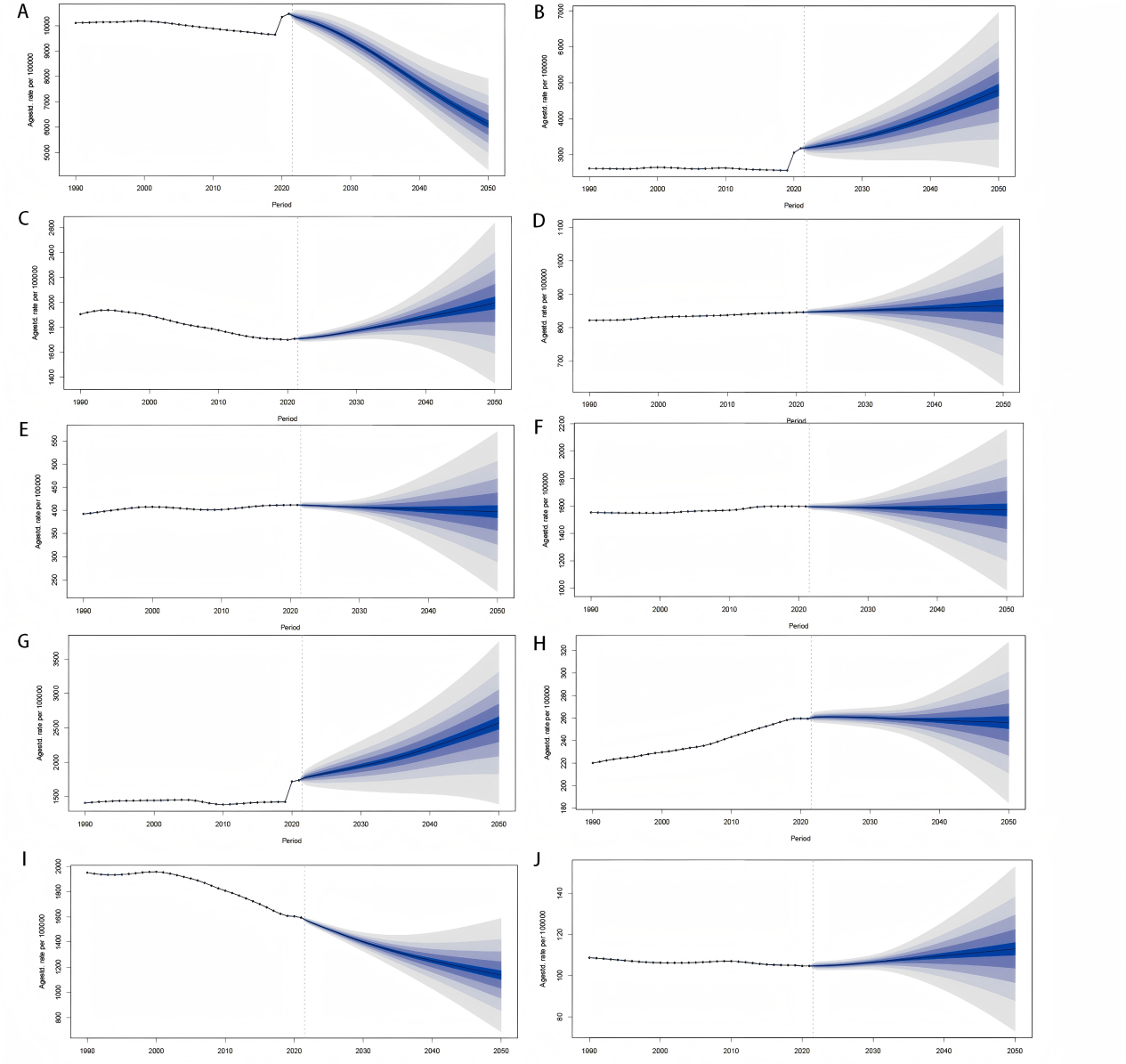


**Supplementary Figure 29.** Predictions age-standardized prevalence rates in both sexes combined for mental disorders in globally from 2022 to 2050. (A) Mental disorders; (B) Anxiety disorders; (C) ADHD; (D) ASD; (E) Bipolar disorder; (F) Conduct disorder; (G) Depressive disorders; (H) Eating disorders; (I) IDII; (J) Schizophrenia. Abbreviation: ADHD, attention-deficit/hyperactivity disorder; ASD, autism spectrum disorders; IDII, idiopathic developmental intellectual disability.
