# Supplementary material for: Global burden and trends of major mental disorders in individuals under 24 years of age from 1990 to 2021, with projections to 2050: insights from the Global Burden of Disease Study 2021
Source: Front Public Health. 2025 Sep 16;13:1635801. doi: 10.3389/fpubh.2025.1635801 (PMC12481897; doi:10.3389/fpubh.2025.1635801)
Supplement: Supplementary file 2 [file Table_1.docx]

Supplementary Material

# Supplementary Tables

**Supplementary Table 1.** Categories of causes and their operational definitions

| Mental Disorders | ICD-10 codes |
| --- | --- |
| Anxiety disorders | F40-42, F43.0, F43.1, F93.0-93.2, F93.8 |
| Attention-deficit/hyperactivity disorder | F90 |
| Autism spectrum disorders | 84.0, F84.1, F84.2, F84.3, F84.4, F84.5, F84.8, F84.9 |
| Bipolar disorder | F30.0-F30.9, F31.0–F31.6, F31.8–F31.9, F34.0 |
| Conduct disorder | F91 |
| Depressive disorders | F32.0–F33.3 |
| Eating disorders | F50.9 |
| Idiopathic developmental intellectual disability |  |
| Schizophrenia | F20 |

**Supplementary Table 2.** Temporal change of ASPR and ASDR for mental disorders at the global level and different regions.

| Location | ASPR | | | | | ASDR | | | | |
| --- | --- | --- | --- | --- | --- | --- | --- | --- | --- | --- |
|  | Time | APC | Lower CI | Upper CI | P | Time | APC | Lower CI | Upper CI | P |
| Global | 1990-2000 | 0.0762 | 0.0619 | 0.0905 | <0.001 | 1990-2019 | -0.0168 | -0.0422 | 0.0086 | 0.1870 |
|  | 2000-2019 | -0.2831 | -0.2889 | -0.2773 | <0.001 | 2019-2021 | 6.6356 | 4.8786 | 8.4220 | <0.001 |
|  | 2019-2021 | 4.7397 | 4.5492 | 4.9305 | <0.001 |  |  |  |  |  |
| High-income Asia Pacific | 1990-1995 | -0.0723 | -0.1186 | -0.0260 | 0.0046 | 1990-1995 | 0.0168 | -0.0333 | 0.0669 | 0.4858 |
|  | 1995-2000 | 0.4445 | 0.3812 | 0.5078 | <0.001 | 1995-2000 | 0.6468 | 0.5757 | 0.7178 | <0.001 |
|  | 2000-2004 | -0.3076 | -0.4028 | -0.2124 | <0.001 | 2000-2005 | -0.1774 | -0.2477 | -0.1071 | <0.001 |
|  | 2004-2014 | 0.0147 | -0.0024 | 0.0319 | 0.0870 | 2005-2011 | 0.2021 | 0.1521 | 0.2521 | <0.001 |
|  | 2014-2019 | -0.3192 | -0.3807 | -0.2576 | <0.001 | 2011-2019 | -0.1044 | -0.1343 | -0.0746 | <0.001 |
|  | 2019-2021 | 4.2351 | 4.0204 | 4.4502 | <0.001 | 2019-2021 | 5.2331 | 4.9919 | 5.4750 | <0.001 |
| High-income North America | 1990-2001 | 0.9466 | 0.7269 | 1.1668 | <0.001 | 1990-2001 | 1.3579 | 1.1442 | 1.5721 | <0.001 |
|  | 2001-2018 | -0.1353 | -0.2606 | -0.0098 | 0.0358 | 2001-2019 | -0.0960 | -0.2060 | 0.0141 | 0.0845 |
|  | 2018-2021 | 4.9621 | 3.2819 | 6.6697 | <0.001 | 2019-2021 | 11.0342 | 7.5709 | 14.6091 | <0.001 |
| Central Asia | 1990-2000 | 0.3584 | 0.2953 | 0.4216 | <0.001 | 1990-2019 | -0.0166 | -0.0296 | -0.0036 | 0.0143 |
|  | 2000-2019 | -0.2835 | -0.3092 | -0.2578 | <0.001 | 2019-2021 | 6.9793 | 6.0744 | 7.8919 | <0.001 |
|  | 2019-2021 | 5.0334 | 4.1635 | 5.9107 | <0.001 |  |  |  |  |  |
| East Asia | 1990-1998 | 0.6785 | 0.5577 | 0.7995 | <0.001 | 1990-2001 | 0.2521 | 0.1422 | 0.3622 | <0.001 |
|  | 1998-2019 | -0.2908 | -0.3207 | -0.2608 | <0.001 | 2001-2014 | -0.4577 | -0.5531 | -0.3622 | <0.001 |
|  | 2019-2021 | 0.9544 | -0.1638 | 2.0850 | 0.0911 | 2014-2021 | 0.0367 | -0.1809 | 0.2548 | 0.7309 |
| South Asia | 1990-2002 | -0.5645 | -0.5950 | -0.5340 | <0.001 | 1990-2001 | -0.4214 | -0.5338 | -0.309 | <0.001 |
|  | 2002-2013 | -0.1809 | -0.2197 | -0.1421 | <0.001 | 201-2019 | -0.0195 | -0.0778 | 0.0389 | 0.4975 |
|  | 2013-2019 | -0.6251 | -0.7330 | -0.5171 | <0.001 | 2019-2021 | 5.8317 | 4.0641 | 7.6294 | <0.001 |
|  | 2019-2021 | 3.6771 | 3.1824 | 4.1742 | <0.001 |  |  |  |  |  |
| Southeast Asia | 1990-1994 | -0.4909 | -0.5048 | -0.4770 | <0.001 | 1990-1995 | -0.1923 | -0.2073 | -0.1773 | <0.001 |
|  | 1994-2000 | -0.3361 | -0.3458 | -0.3263 | <0.001 | 1995-2000 | -0.2547 | -0.2757 | -0.2337 | <0.001 |
|  | 2000-2005 | -0.1909 | -0.2046 | -0.1772 | <0.001 | 200-2005 | 0.0785 | 0.0574 | 0.0996 | <0.001 |
|  | 2005-2011 | -0.4282 | -0.4378 | -0.4186 | <0.001 | 2005-2010 | -0.1784 | -0.1994 | -0.1574 | <0.001 |
|  | 2011-2019 | -0.2330 | -0.2388 | -0.2272 | <0.001 | 2010-2015 | 0.1996 | 0.1784 | 0.2207 | <0.001 |
|  | 2019-2021 | 5.5555 | 5.5101 | 5.601 | <0.001 | 2015-2019 | 0.0278 | -0.0057 | 0.0614 | 0.0955 |
|  |  |  |  |  |  | 2019-2021 | 7.4581 | 7.3843 | 7.5320 | <0.001 |
| Australasia | 1990-1999 | -0.1916 | -0.2176 | -0.1657 | <0.001 | 1990-1999 | -0.281 | -0.309 | -0.2530 | <0.001 |
|  | 1999-2006 | 0.3530 | 0.3106 | 0.3954 | <0.001 | 1999-2008 | 0.4614 | 0.4285 | 0.4943 | <0.001 |
|  | 2006-2019 | 0.2046 | 0.1902 | 0.2191 | <0.001 | 2008-2019 | 0.2513 | 0.2276 | 0.2750 | <0.001 |
|  | 2019-2021 | 1.5615 | 1.2606 | 1.8633 | <0.001 | 2019-2021 | 2.2791 | 1.9572 | 2.6021 | <0.001 |
| Caribbean | 1990-2019 | -0.0278 | -0.0383 | -0.0173 | <0.001 | 1990-2011 | -0.1913 | -0.1990 | -0.1835 | <0.001 |
|  | 2019-2021 | 4.9435 | 4.2176 | 5.6746 | <0.001 | 2011-2019 | 0.1862 | 0.1454 | 0.2271 | <0.001 |
|  |  |  |  |  |  | 2019-2021 | 7.5422 | 7.2037 | 7.8819 | <0.001 |
| Central Europe | 1990-2015 | -0.1539 | -0.1658 | -0.1420 | <0.001 | 1990-2014 | -0.1001 | -0.1106 | -0.0895 | <0.001 |
|  | 2015-2019 | 0.1788 | -0.1210 | 0.4795 | 0.2306 | 2014-2019 | 0.3069 | 0.1467 | 0.4674 | 0.0006 |
|  | 2019-2021 | 7.6066 | 6.9436 | 8.2737 | <0.001 | 2019-2021 | 9.6973 | 9.1233 | 10.2744 | <0.001 |
| Eastern Europe | 1990-2000 | 0.2140 | 0.2030 | 0.2251 | <0.001 | 1990-1997 | 0.0810 | 0.0422 | 0.1197 | 0.0003 |
|  | 2000-2005 | -0.1941 | -0.2388 | -0.1494 | 0.2306 | 1997-2010 | -0.1389 | -0.1560 | -0.1218 | <0.001 |
|  | 2005-2010 | -0.3937 | -0.4381 | -0.3493 | <0.001 | 2010-2019 | 0.1081 | 0.0766 | 0.1397 | <0.001 |
|  | 2010-2019 | 0.0065 | -0.0089 | 0.0218 | 0.3877 | 2019-2021 | 10.6358 | 10.3096 | 10.9629 | <0.001 |
|  | 2019-2021 | 8.3143 | 8.1598 | 8.4691 | <0.001 |  |  |  |  |  |
| Western Europe | 1990-2019 | 0.0566 | 0.0329 | 0.0804 | <0.001 | 1990-2019 | 0.0333 | 0.0189 | 0.0477 | <0.001 |
|  | 2019-2021 | 7.4934 | 5.7128 | 9.3041 | <0.001 | 2019-2021 | 9.7199 | 8.6884 | 10.7611 | <0.001 |
| Andean Latin America | 1990-2019 | -0.0228 | -0.026 | -0.0196 | <0.001 | 1990-2019 | 0.0304 | 0.0267 | 0.0342 | <0.001 |
|  | 2019-2021 | 10.9398 | 10.6975 | 11.1826 | <0.001 | 2019-2021 | 15.4013 | 15.1096 | 15.6938 | <0.001 |
| Central Latin Americ | 1990-2004 | 0.5002 | 0.3338 | 0.6668 | <0.001 | 1990-2004 | 0.6143 | 0.4019 | 0.8271 | <0.001 |
|  | 2004-2018 | -0.2556 | -0.4374 | -0.0736 | 0.0079 | 2004-2018 | -0.2249 | -0.4618 | 0.0127 | 0.0624 |
|  | 2018-2021 | 4.9440 | 3.0639 | 6.8584 | <0.001 | 2018-2021 | 6.4295 | 4.0006 | 8.9151 | <0.001 |
| Southern Latin America | 1990-2019 | -0.0816 | -0.1075 | -0.0558 | <0.001 | 1990-2019 | -0.0639 | -0.0985 | -0.0293 | 0.0008 |
|  | 2019-2021 | 9.1860 | 7.0984 | 11.3143 | <0.001 | 2019-2021 | 11.0577 | 8.4751 | 13.7018 | <0.001 |
| Tropical Latin America | 1990-2005 | 0.8112 | 0.7763 | 0.8462 | <0.001 | 1990-2005 | 1.2238 | 1.1794 | 1.2683 | <0.001 |
|  | 2005-2011 | -1.3782 | -1.5633 | -1.1928 | <0.001 | 2005-2011 | -2.2100 | -2.4383 | -1.9811 | <0.001 |
|  | 2011-2019 | 0.0602 | -0.0537 | 0.1743 | 0.2839 | 2011-2019 | -0.0750 | -0.2143 | 0.0645 | 0.2759 |
|  | 2019-2021 | 6.9753 | 6.0502 | 7.9085 | <0.001 | 2019-2021 | 9.4863 | 8.3228 | 10.6623 | <0.001 |
| North Africa and Middle East | 1990-2019 | -0.1208 | -0.1434 | -0.0981 | <0.001 | 1990-2019 | 0.0335 | 0.0286 | 0.0384 | <0.001 |
|  | 2019-2021 | 5.8151 | 4.2048 | 7.4503 | <0.001 | 2019-2021 | 7.9359 | 7.5874 | 8.2855 | <0.001 |
| Oceania | 1990-2019 | -0.0151 | -0.0235 | -0.0068 | 0.0009 | 1990-2019 | -0.0227 | -0.0279 | -0.0175 | <0.001 |
|  | 2019-2021 | 2.2016 | 1.6172 | 2.7893 | <0.001 | 2019-2021 | 3.1175 | 2.7562 | 3.4801 | <0.001 |
| Central Sub-Saharan Africa | 1990-2004 | 0.2697 | 0.1614 | 0.3781 | <0.001 | 1990-2018 | -0.0127 | -0.0595 | 0.0342 | 0.5828 |
|  | 2004-2018 | -0.1501 | -0.2698 | -0.0302 | 0.0163 | 2018-2021 | 3.1320 | 1.6456 | 4.6401 | 0.0002 |
|  | 2018-2021 | 2.4533 | 1.2470 | 3.6741 | 0.0003 |  |  |  |  |  |
| Eastern Sub-Saharan Africa | 1990-2001 | 0.0650 | 0.0397 | 0.0904 | <0.001 | 1990-2001 | 0.0524 | 0.0267 | 0.0782 | 0.0004 |
|  | 2001-2012 | -0.2918 | -0.3206 | -0.2629 | <0.001 | 2001-2012 | -0.2284 | -0.2580 | -0.1988 | <0.001 |
|  | 2012-2019 | 0.0161 | -0.0473 | 0.0795 | 0.6038 | 2012-2019 | 0.1569 | 0.0917 | 0.2221 | <0.001 |
|  | 2019-2021 | 4.7310 | 4.3271 | 5.1366 | <0.001 | 2019-2021 | 5.9109 | 5.5003 | 6.3230 | <0.001 |
| Southern Sub-Saharan Africa | 1990-2019 | 0.0492 | 0.0367 | 0.0617 | <0.001 | 1990-2019 | 0.0874 | 0.0705 | 0.1043 | <0.001 |
|  | 2019-2021 | 8.7617 | 7.8641 | 9.6668 | <0.001 | 2019-2021 | 10.5166 | 9.2947 | 11.7521 | <0.001 |
| Western Sub-Saharan Africa | 1990-2004 | 0.2362 | 0.1482 | 0.3243 | <0.001 | 1990-2005 | 0.2486 | 0.1482 | 0.3490 | <0.001 |
|  | 2004-2018 | -0.2852 | -0.3823 | -0.1881 | <0.001 | 2005-2018 | -0.2546 | -0.3938 | -0.1152 | 0.0009 |
|  | 2018-2021 | 1.7140 | 0.7652 | 2.6718 | 0.0010 | 2018-2021 | 2.0613 | 0.8547 | 3.2823 | 0.0017 |
| SDI regions |  |  |  |  |  |  |  |  |  |  |
| High SDI | 1990-2002 | 0.2060 | 0.1745 | 0.2375 | <0.001 | 1990-2019 | 0.2781 | 0.2304 | 0.3258 | <0.001 |
|  | 2002-2021 | 6.2394 | 4.0711 | 8.4529 | <0.001 | 2019-2021 | 8.4362 | 5.1374 | 11.8384 | <0.001 |
| High-middle SDI | 1990-1999 | 0.3389 | 0.3042 | 0.3736 | <0.001 | 1990-2001 | 0.1370 | 0.0985 | 0.1756 | <0.001 |
|  | 1999-2009 | -0.2275 | -0.2611 | -0.1939 | <0.001 | 2001-2010 | -0.1372 | -0.1993 | -0.0751 | 0.0002 |
|  | 2009-2019 | -0.0067 | -0.0403 | 0.0269 | 0.6822 | 2010-2019 | 0.2882 | 0.2258 | 0.3506 | <0.001 |
|  | 2019-2021 | 4.8200 | 4.4111 | 5.2305 | <0.001 | 2019-2021 | 6.2035 | 5.5820 | 6.8286 | <0.001 |
| Middle SDI | 1990-2000 | 0.1904 | 0.1604 | 0.2205 | <0.001 | 1990-2000 | 0.2941 | 0.2197 | 0.3685 | <0.001 |
|  | 2000-2019 | -0.3039 | -0.3159 | -0.2919 | <0.001 | 2000-2019 | -0.1214 | -0.1520 | -0.0907 | <0.001 |
|  | 2019-2021 | 4.9438 | 4.5498 | 5.3393 | <0.001 | 2019-2021 | 7.1142 | 6.0641 | 8.1748 | <0.001 |
| Low-middle SDI | 1990-2019 | -0.3562 | -0.3666 | -0.3457 | <0.001 | 1990-2019 | -0.1167 | -0.1289 | -0.1044 | <0.001 |
|  | 2019-2021 | 4.2215 | 3.5531 | 4.8942 | <0.001 | 2019-2021 | 6.8846 | 6.0387 | 7.7372 | <0.001 |
| Low SDI | 1990-2002 | 0.0575 | 0.0404 | 0.0747 | <0.001 | 1990-2019 | -0.0385 | -0.0570 | -0.0200 | 0.0002 |
|  | 2002-2019 | -0.3064 | -0.3174 | -0.2955 | <0.001 | 2019-2021 | 4.8886 | 3.6400 | 6.1522 | <0.001 |
|  | 2019-2021 | 3.4726 | 3.1783 | 3.7677 | <0.001 |  |  |  |  |  |

ASPR, age-standardized prevalence rate; ASDR, age-standardized death rate; APC, annual percentage change; CI, confidence interval; SDI, Socio-Demographic Index

**Supplementary Table 3.** Global prevalence and DALY for 9 mental disorders in 1990 and 2021.

| **Cause** | **Prevalence** | | | | | **DALYs** | | | | |
| --- | --- | --- | --- | --- | --- | --- | --- | --- | --- | --- |
|  | Number (95% UI) | | ASPR (95% UI) | | AAPC (95% CI) | Number (95% UI) | | ASDR (95% UI) | | AAPC (95% CI) |
|  | 1990 | 2021 | 1990 | 2021 |  | 1990 | 2021 | 1990 | 2021 |  |
| **Mental disorders** | |  |  |  |  |  |  |  |  |  |
| Total | 277736208.65(236539575.95,322310108.38) | 346358694.87(296686630.84,401435795.20) | 10075.76(8578.00,11697.92) | 10433.45(8925.95,12104.94) | 0.15(0.14,0.16) | 31207306.29(22591423.08,41316442.56) | 42264639.03(30223443.26,56412619.83) | 1128.12(816.40,1493.91) | 1266.19(905.51,1690.17) | 0.40(0.29,0.51) |
| Female | 130424137.12(110486446.26,152099827.04) | 166883701.28(141730054.33,194663687.35) | 9621.63(8145.16,11226.03) | 10292.05(8728.78,12017.74) |  | 15897249.67(11346064.78,21289006.92) | 21819618.31(15408423.05,29369989.09) | 1166.88(832.51,1563.25) | 1336.77(943.97,1799.68) |  |
| Male | 147312071.53(124969883.80,171363828.03) | 179474993.59(153779955.22,207267482.61) | 10501.73(8908.27,12218.14) | 10558.42(9035.42,12206.62) |  | 15310056.62(11148209.23,20153200.20) | 20445020.73(14732414.90,26982770.40) | 1089.17(792.85,1433.91) | 1198.02(863.28,1581.17) |  |
| **Anxiety disorders** | |  |  |  |  |  |  |  |  |  |
| Total | 71843023.90(52837765.78,95657347.94) | 105816766.92(76873291.96,142133743.44) | 2598.55(1909.46,3462.15) | 3157.18(2291.84,4243.89) | 0.69(0.56,0.84) | 8831519.60(5477308.66,13163463.63) | 13009156.15(8003143.66,19465065.40) | 319.49(198.04,476.21) | 388.23(238.69,581.05) | 0.70(0.56,0.63) |
| Female | 43880851.38(32376169.99,58306956.04) | 64413211.20(46834036.97,86205696.86) | 3227.53(2378.96,4292.11) | 3941.73(2863.41,5280.02) |  | 5374332.71(3334857.89,7935644.69) | 7882198.92(4854766.00,11721113.94) | 395.42(245.18,583.94) | 482.51(296.98,717.72) |  |
| Male | 27962172.53(20572310.54,37463132.29) | 41403555.71(29858440.00,55947593.75) | 1990.00(1463.02,2667.34) | 2410.80(1737.48,3259.46) |  | 3457186.89(2138466.06,5175931.15) | 5126957.24(3126344.67,7724044.55) | 246.06(152.17,368.39) | 298.56(181.97,449.88) |  |
| **ADHD** | |  |  |  |  |  |  |  |  |  |
| Total | 52128916.81(35744377.47,74609035.72) | 56371474.11(38524577.03,80455487.99) | 1901.94(1304.14,2723.24) | 1705.75(1165.44,2435.75) | -0.36(-0.40,-0.33) | 637624.96(330716.08,1087853.99) | 690263.81(357187.83,1176154.82) | 23.27(12.06,39.72) | 20.89(10.80,35.62) | -0.36(-0.40,-0.32) |
| Female | 14433948.86(9839679.62,20671759.74) | 15395318.38(10424509.48,22088117.70) | 9540.08(8177.55,11038.35) | 957.81(648.44,1375.04) |  | 176013.37(91052.15,300449.67) | 187863.71(97908.49,321588.66) | 13.10(6.78,22.38) | 11.69(6.09,20.03) |  |
| Male | 37694967.94(25851614.14,53877437.18) | 40976155.73(28101631.26,58421906.45) | 2696.79(1849.56,3855.48) | 2412.93(1654.43,3441.56) |  | 461611.59(239998.13,785779.20) | 502400.09(258955.95,852580.26) | 33.03(17.17,56.24) | 29.59(15.24,50.24) |  |
| **ASD** | |  |  |  |  |  |  |  |  |  |
| Total | 22611068.69(19015422.62,26723992.63) | 27329738.94(23041655.33,32118309.45) | 822.19(691.45,971.74) | 846.43(713.61,994.84) | 0.09(0.08,0.10) | 4318654.75(2944745.26,6076430.03) | 5234428.09(3544010.08,7365061.61) | 157.05(107.09,220.98) | 162.16(109.81,228.15) | 0.10(0.09,0.11) |
| Female | 7199122.76(6022717.81,8550228.51) | 8764174.15(7344345.91,10417720.03) | 535.47(447.98,635.94) | 558.79(468.28,664.25) |  | 1370753.11(941994.85,1932625.46) | 1671103.34(1143881.88,2353807.40) | 101.98(70.08,143.78) | 106.61(72.98,150.16) |  |
| Male | 15411945.93(12983792.78,18144309.48) | 18565564.79(15674473.17,21714693.70) | 1096.65(923.88,1291.06) | 1118.20(944.05,1307.96) |  | 2947901.64(2010249.81,4147784.68) | 3563324.75(2422330.68,5024094.37) | 209.77(143.04,295.16) | 214.66(145.94,302.64) |  |
| **Bipolar disorder** | |  |  |  |  |  |  |  |  |  |
| Total | 6148853.53(4496267.79,8378233.52) | 7813742.28(5658134.73,10785407.89) | 219.37(160.36,298.96) | 230.10(166.59,317.65) | 0.15(0.14,0.17) | 1366382.33(837981.97,2120215.70) | 1736701.87(1062784.83,2688554.92) | 48.75(29.88,75.66) | 51.15(31.29,79.19) | 0.16(0.13,0.18) |
| Female | 3169123.60(2304980.33,4331929.72) | 3973133.53(2865865.84,5504728.97) | 228.98(166.48,313.10) | 239.18(172.48,331.46) |  | 699057.96(428863.95,1084436.68) | 875646.08(531823.40,1359846.23) | 50.52(30.97,78.38) | 52.72(32.00,81.90) |  |
| Male | 2979729.93(2190631.82,4038818.18) | 3840608.75(2795944.17,5279549.02) | 209.98(154.34,284.64) | 221.40(161.17,304.37) |  | 667324.37(410412.59,1030627.72) | 861055.79(526953.23,1335626.03) | 47.03(28.91,72.64) | 49.64(30.37,77.00) |  |
| **Conduct disorder** | |  |  |  |  |  |  |  |  |  |
| Total | 32734708.97(22281353.44,45382485.46) | 41043975.21(27745686.29,56975579.11) | 1205.98(820.84,1671.00) | 1239.85(837.97,1721.16) | 0.09(0.08,0.10) | 3981146.09(2092549.62,6515686.67) | 5002614.36(2607480.60,8142030.29) | 146.68(77.11,240.13) | 151.13(78.77,246.03) | 0.10(0.08,0.12) |
| Female | 11353864.88(7350398.38,16454744.80) | 14391633.92(9260447.97,20769003.86) | 856.60(554.39,1240.38) | 896.37(576.71,1293.28) |  | 1375158.66(707841.81,2328649.54) | 1744782.93(886774.26,2936201.18) | 103.77(53.44,175.74) | 108.69(55.24,182.91) |  |
| Male | 21380844.09(14786631.85,29196137.79) | 26652341.29(18283414.56,36526600.93) | 1540.49(1065.43,2102.96) | 1563.65(1072.41,2143.49) |  | 2605987.43(1387721.56,4235826.16) | 3257831.43(1719205.67,5275949.27) | 187.77(100.01,305.26) | 191.14(100.88,309.63) |  |
| **Depressive disorders** | |  |  |  |  |  |  |  |  |  |
| Total | 39047669.14(29184241.48,52036362.64) | 58390936.75(42770269.39,79478701.64) | 1396.73(1043.13,1861.90) | 1723.77(1261.86,2346.51) | 0.76(0.69,0.84) | 7130860.32(4492222.04,10667259.31) | 10891632.36(6750115.42,16505430.37) | 255.13(160.64,381.73) | 321.62(199.23,487.53) | 0.85(0.78,0.92) |
| Female | 24167764.20(18062817.77,32206059.35) | 35275603.54(25879246.71,47982690.74) | 1751.91(1308.10,2335.36) | 2131.04(1562.12,2898.74) |  | 4428838.38(2794595.69,6616441.62) | 6570469.98(4085035.68,9921042.32) | 321.16(202.51,479.90) | 397.11(246.74,599.81) |  |
| Male | 14879904.94(11091468.83,19789224.89) | 23115333.21(16822275.10,31382847.49) | 1334.78(971.03,1812.47) | 1050.82(782.86,1397.89)3) |  | 2702021.94(1689434.36,4066643.08) | 4321162.38(2659682.53,6572852.90) | 190.84(119.27,287.26) | 249.55(153.55,379.67) |  |
| **Eating disorders** | |  |  |  |  |  |  |  |  |  |
| Total | 4752226.85(3004437.10,7266945.30) | 6790278.12(4261905.60,10531665.95) | 169.85(107.41,259.79) | 200.19(125.65,310.61) | 0.53(0.51,0.55) | 1019412.40(551637.01,1712225.74) | 1455225.51(781825.15,2477292.35) | 36.44(19.72,61.22) | 42.91(23.06,73.07) | 0.53(0.50,0.54) |
| Female | 3100756.69(2010719.81,4630953.53) | 4335551.58(2784300.76,6532654.48) | 224.47(145.59,335.41) | 261.28(167.80,394.00) |  | 663062.84(368392.54,1090355.86) | 925136.14(509636.18,1537014.04) | 48.01(26.68,78.98) | 55.76(30.73,92.69) |  |
| Male | 1651470.16(985862.77,2724036.88) | 2454726.53(1449819.34,4092314.57) | 116.59(69.62,192.29) | 141.66(83.68,236.19) |  | 356349.56(181995.50,636233.26) | 530089.37(266148.14,959489.46) | 25.16(12.85,44.91) | 30.59(15.36,55.38) |  |
| **IDII** | |  |  |  |  |  |  |  |  |  |
| Total | 53632754.78(30248683.71,75894270.58) | 51526991.60(28880678.24,73549629.85) | 1953.34(1102.02,2763.59) | 1594.52(893.22,2276.78) | -0.64(-0.67,-0.62) | 2208458.68(1052759.87,3753587.60) | 2217223.89(1050719.85,3760228.68) | 80.45(38.36,136.70) | 68.64(32.54,116.46) | -0.51(-0.58,-0.43) |
| Female | 26046882.86(15467686.78,36139133.98) | 25613751.18(15243704.62,35700190.35) | 1941.69(1153.64,2693.39) | 1631.05(970.21,2274.13) |  | 1068607.66(532933.24,1779891.60) | 1098753.99(555984.53,1813773.94) | 79.70(39.77,132.71) | 70.03(35.45,115.63) |  |
| Male | 27585871.92(14814282.00,39769872.30) | 25913240.41(13679405.28,37841627.07) | 1964.68(1055.22,2832.00) | 1559.94(823.26,2278.82) |  | 1139851.02(516120.15,1974284.57) | 1118469.90(492987.95,1946117.72) | 81.18(36.76,140.58) | 67.33(29.68,117.22) |  |
| **Schizophrenia** | |  |  |  |  |  |  |  |  |  |
| Total | 1709490.00(1145454.71,2452935.83) | 1995272.29(1279779.36,2946251.96) | 60.70(40.66,87.13) | 58.48(37.50,86.36) | -0.12(-0.13,-0.11) | 1143102.77(711043.67,1734718.57) | 1332728.36(831870.95,2062277.77) | 40.59(25.24,61.62) | 39.06(24.38,60.45) | -0.13(-0.14,-0.11) |
| Female | 794719.12(530957.19,1143845.58) | 914572.34(585059.48,1358629.03) | 57.01(38.07,82.09) | 54.64(34.94,81.19) |  | 526119.81(326317.14,799496.08) | 603965.66(373453.00,937396.50) | 37.75(23.40,57.38) | 36.09(22.31,56.02) |  |
| Male | 914770.88(613510.96,1310604.47) | 1080699.95(693956.94,1587250.18) | 64.32(43.13,92.17) | 62.17(39.92,91.32) |  | 616982.96(383536.87,935625.59) | 728762.70(453063.81,1132942.01) | 43.38(26.96,65.80) | 41.92(26.06,65.18) |  |

ASPR, age-standardized prevalence rate; ASDR, age-standardized death rate; AAPC, average annual percentage change; UI, uncertainty interval; CI, confidence interval; ADHD, attention-deficit hyperactivity disorder; ASD, autism spectrum disorders; IDII, idiopathic developmental intellectual disability.

**Supplementary Table 4.** Changes in prevalence and DALYs number according to population-level determinants and causes from 1990 to 2021.

| **Location** | **Prevalence** (% contribute to the total changes) | | | | **DALYs** (% contribute to the total changes) | | | |
| --- | --- | --- | --- | --- | --- | --- | --- | --- |
|  | Overall difference | Aging | Population | Epidemiological change | Overall difference | Aging | Population | Epidemiological change |
| Global | 68622486.21 | 7090091.64 (10.32%) | 50164595.63 (73.12%) | 11367798.94 (16.57%) | 11057332.74 | 927501.67  (8.39%) | 5884886.8  (53.22%) | 4244944.27  (38.39%) |
| **Sex** |  |  |  |  |  |  |  |  |
| Female | 36459564.16 | 3263827.38  (8.95%) | 22920362.23  (62.87) | 10275374.55  (28.18) | 5922368.64 | 467318.22  (7.89%) | 2899474.72  (48.96%) | 2555575.69  (43.15%) |
| Male | 32162922.05 | 3801527.30  (11.82%) | 27292587.99  (84.86) | 1068806.76  (3.32) | 5134964.11 | 451877.53  (8.80%) | 2973946.93  (57.92%) | 1709139.64  (33.28%) |
| **SDI regions** |  |  |  |  |  |  |  |  |
| High SDI | 4440964.14 | -8762814.31 (-197.32%) | 6397189.73 (144.05%) | 6806588.72 (153.27%) | 875957.72 | -1185643.77  (-135.35%) | 860194.62  (98.20%) | 1201406.87  (137.15%) |
| High-middle SDI | -5001686.53 | -16752593.35 (334.94%) | 7258927.39 (-145.13%) | 4491979.43 (-89.81%) | -446857.46 | -2066787.80  (462.52%) | 868521.59  (-194.36%) | 751408.74  (-168.15%) |
| Middle SDI | 5055877.55 | -15526606.16 (-307.10%) | 15606233.89 (308.68%) | 4976249.81 (98.43%) | 1400250.33 | -1792179.32  (-127.99%) | 1776749.84  (126.89%) | 1415679.81  (101.10%) |
| Low-middle SDI | 27612574.24 | 16886903.11 (61.16%) | 13634736.30 (49.38%) | -2909065.18 (-10.54%) | 4431556.83 | 2138605.59  (48.26%) | 1512689.53  (34.13%) | 780261.72  (17.61%) |
| Low SDI | 36478915.66 | 28751053.12 (78.82%) | 7339161.88 (20.12%) | 388700.66 (1.07%) | 4790501.73 | 3494299.34  (72.94%) | 874281.14  (18.25%) | 421921.25  (8.81%) |
| **Regions** |  |  |  |  |  |  |  |  |
| High-income Asia Pacific | -2134835.74 | -3506329.87 (164.24%) | 917320.9 (-42.97%) | 454173.24 (-21.27%) | -245336.80 | -469727.27  (191.46%) | 122904.39  (-50.10%) | 101486.08  (-41.37%) |
| High-income North America | 5262637.61 | -410598.59 (-7.80%) | 2445410.99 (46.47%) | 3227825.22 (61.33%) | 875545.87 | -52050.35  (-5.94%) | 330595.69  (37.76%) | 597000.53  (68.19%) |
| Central Asia | 541055.69 | -163258.15 (-30.17%) | 503246.18 (93.01%) | 201067.66 (37.16%) | 85201.11 | -20448.96  (-24.00%) | 61741.18  (72.47%) | 43908.89  (51.54%) |
| East Asia | -16719007.96 | -25780074.28 (154.20%) | 8066744.19 (-48.25%) | 994322.13 (-5.95%) | -2126830.55 | -2838069.24  (133.44%) | 836933.61  (-39.35%) | -125694.91  (5.91%) |
| South Asia | 23482165.45 | 15454355.10 (65.81%) | 13103548.60 (55.80%) | -5075738.25 (-21.62%) | 269091.32 | 1933699.58  (51.78%) | 1329004.33  (35.59%) | 472013.56  (12.64%) |
| Southeast Asia | 3220281.82 | -1139524.99 (-35.39%) | 3925097.15 (121.89%) | 434709.66 (13.50%) | 740572.50 | -95929.40  (-12.95%) | 463974.24  (62.65%) | 372527.66  (50.30%) |
| Australasia | 351251.05 | 28152.85 (8.02%) | 236713.45 (67.39%) | 86384.75 (24.59%) | 48751.11 | 2111.30  (4.33%) | 30894.16  (63.37%) | 15745.66  (32.30%) |
| Caribbean | 307361.04 | -263430.18 (-85.71%) | 386809.16 (125.85%) | 183982.06 (59.86%) | 40374.49 | -27076.23  (-67.06%) | 41105.47  (101.81%) | 26345.25  (65.25%) |
| Central Europe | -1259777.30 | -2268478.68 (180.07%) | 583633.09 (-46.33%) | 425068.29 (-33.74%) | -132399.49 | -284281.01  (214.71%) | 73420.57  (-55.45%) | 78460.94  (-59.26%) |
| Eastern Europe | -1345450.19 | -3392099.16 (252.12%) | 1058069.13 (-78.64%) | 988579.84 (-73.48%) | -138073.03 | -440012.81  (318.68%) | 135209.69  (-97.93%) | 166730.10  (-120.76%) |
| Western Europe | 88963.73 | -5027907.48 (-5651.64%) | 2888432.13 (3246.75%) | 2228439.08 (2504.88%) | 58933.81 | -717483.02  (-1217.44%) | 397291.19  (674.13%) | 379125.64  (643.31%) |
| Andean Latin America | 1471345.83 | 485509.40 (33.00%) | 496512.05 (33.75%) | 489324.38 (33.26%) | 201615.95 | 62258.31  (30.88%) | 56946.53  (28.25%) | 82411.10  (40.88%) |
| Central Latin Americ | 3123376.77 | -83734.40 (-2.68%) | 1646001.27 (52.70%) | 1561109.90 (49.98%) | 485651.37 | 11288.34  (2.32%) | 205271.72  (42.27%) | 269091.32  (55.41%) |
| Southern Latin America | 762772.96 | -107973.28 (-14.16%) | 490736.90 (64.34%) | 380009.34 (49.82%) | 124341.85 | -9614.74  (-7.73%) | 66827.18  (53.74%) | 67129.41  (53.99%) |
| Tropical Latin America | 2198888.80 | -1290084.68 (-58.67%) | 1747899.32 (79.49%) | 1741074.15 (79.18%) | 360662.78 | -123199.54  (-34.16%) | 210455.28  (58.35%) | 273407.05  (75.81%) |
| North Africa and Middle East | 13840730.65 | 6626305.42 (47.88%) | 5142784.79 (37.16%) | 2071640.45 (14.97%) | 2034324.09 | 875701.40  (43.05%) | 637336.13  (31.33%) | 521286.56  (25.62%) |
| Oceania | 340585.88 | 239064.67 (70.19%) | 80079.11 (23.51%) | 21442.1 (6.3%) | 41980.56 | 28661.56  (68.27%) | 9528.39  (22.70%) | 3790.61  (9.03%) |
| Central Sub-Saharan Africa | 5005487.32 | 3771428.84 (75.35%) | 834213.67 (16.67%) | 399844.81 (7.99%) | 694741.04 | 525175.77  (75.59%) | 115709.19  (16.66%) | 53856.08  (7.75%) |
| Eastern Sub-Saharan Africa | 13947800.49 | 10295769.44 (73.82%) | 2627431.95 (18.84%) | 1024599.09 (7.35%) | 1969784.42 | 1399906.22  (71.07%) | 352341.05  (17.89%) | 217537.15  (11.04%) |
| Southern Sub-Saharan Africa | 1179352.76 | 204230.09 (17.32%) | 479348.61 (40.65%) | 495774.06 (42.04%) | 180387.70 | 30366.60  (16.83%) | 67303.41  (37.31%) | 82717.69  (45.86%) |
| Western Sub-Saharan Africa | 14957499.54 | 11988202.94 (80.15%) | 2527666.55 (16.90%) | 441630.05 (2.95%) | 2022386.48 | 1594948.01  (78.86%) | 335739.48  (16.60%) | 91699.00  (4.53%) |

SDI, sociodemographic index.

**Supplementary Table 5.** Changes in prevalence and DALYs number according to population-level determinants and causes from 1990 to 2021 for 9 mental disorders.

| **Causes** | **Prevalence** (% contribute to the total changes) | | | | **DALYs** (% contribute to the total changes) | | | |
| --- | --- | --- | --- | --- | --- | --- | --- | --- |
|  | Overall difference | Aging | Population | Epidemiological change | Overall difference | Aging | Population | Epidemiological change |
| **Anxiety disorders** | 33973743.01 | 2730571.65  (8.04) | 14178616.27  (41.73) | 17064555.09  (50.23) | 4177636.55 | 335584.09  (8.03) | 1743048.62  (41.72) | 2099003.84  (50.24) |
| Female | 20532359.82 | 1550246.97  (7.55) | 8302168.92  (40.43) | 10679943.93  (52.02) | 2507866.20 | 189798.82  (7.57) | 1016326.46  (40.53) | 1301740.92  (51.91) |
| Male | 13441383.19 | 1136891.48  (8.46) | 5742713.47  (42.72) | 6561778.24  (48.82) | 1669770.35 | 140622.23  (8.42) | 710622.74  (42.56) | 818525.37  (49.02) |
| **ADHD** | 4242557.31 | 1459757.81  (34.41) | 8780502.78  (206.96) | -5997703.28  (-141.37) | 52638.85 | 17848.43  (33.91) | 107455.74  (204.14) | -72665.32  (-138.05) |
| Female | 961369.52 | 384317.29  (39.98) | 2318516.08  (241.17) | -1741463.86  (-181.14) | 11850.34 | 4681.95  (39.51) | 28282.07  (238.66) | -21113.69  (-178.17) |
| Male | 3281187.79 | 1104784.13  (33.67) | 6608126.67  (201.39) | -4431723.01  (-135.06) | 40788.51 | 13526.51  (33.16) | 80969.51  (198.51) | -53707.52  (-131.67) |
| **ASD** | 4718670.25 | -27921.49  (-0.59) | 4021182.76  (85.22) | 725408.98  (15.37) | 915773.34 | -6076.83  (-0.66) | 769135.85  (83.99) | 152714.33  (16.68) |
| Female | 1565051.39 | -11154.67  (-0.71) | 1233740.90  (78.83) | 342465.16  (21.88) | 300350.23 | -2563.77  (-0.85) | 235085.54  (78.27) | 67828.46  (22.58) |
| Male | 3153618.86 | -15092.22  (-0.48) | 2840100.55  (90.06) | 328610.53  (10.42) | 615423.11 | -3178.69  (-0.52) | 544192.82  (88.43) | 74408.98  （12.09） |
| **Bipolar disorder** | 1664888.75 | 209203.96  (12.57) | 1121722.41  (67.38) | 333962.39  (20.06) | 370319.54 | 46522.71  (12.56) | 249292.40  (67.32) | 74504.43  (20.12) |
| Female | 804009.93 | 96597.88  (12.01) | 551271.71  (68.57) | 156140.35  (19.42) | 176588.12 | 21318.30  (12.07) | 121547.48  (68.83) | 33722.34  (19.10) |
| Male | 860878.82 | 112123.99  (13.02) | 568311.62  (66.02) | 180443.21  (20.96) | 193731.42 | 25133.06  (12.97) | 127347.31  (65.73) | 41251.05  (21.29) |
| **Conduct disorder** | 8309266.24 | 1355471.43  (16.31) | 5930750.41  (71.38) | 1023044.40  (12.31) | 1021468.27 | 165107.74  (16.16) | 722093.33  (70.69) | 134267.20  (13.14) |
| Female | 3037769.04 | 467676.07  (15.40) | 1986134.54  (65.38) | 583958.43  (19.22) | 369624.27 | 56730.79  (15.35) | 240677.51  (65.11) | 72215.97  (19.54) |
| Male | 5271497.20 | 903330.29  (17.14) | 4008743.04  (76.05) | 359423.87  (6.82) | 651844.00 | 110295.39  (16.92) | 489314.22  (75.07) | 52234.40  (8.01) |
| **Depressive disorders** | 19343267.61 | -99726.34  (-0.52) | 7969606.35  (41.20) | 11473387.60  (59.31) | 3760772.04 | 279867.88  (7.44) | 1435900.49  (38.18) | 1435900.49  (54.38) |
| Female | 11107839.34 | -36775.19  (-0.33) | 4660059.46  (41.95) | 6484555.07  (58.38) | 2141631.60 | 156324.97  (7.30) | 842804.61  (39.35) | 1142502.02  (53.35) |
| Male | 8235428.27 | -83918.43  (-1.02) | 3229550.32  (39.22) | 5089796.37  (61.80) | 1619140.45 | 118025.02  (7.29) | 579330.25  (35.78) | 921785.17  (56.93) |
| **Eating disorders** | 2038051.27 | 175993.57  (8.64) | 922417.29  (45.26) | 939640.40  (46.10) | 435813.11 | 37737.36  (8.66) | 197768.30  (45.38) | 200307.45  (45.96) |
| Female | 1234794.90 | 102799.84  (8.33) | 571280.64  (46.27) | 560714.43  (45.41) | 262073.30 | 21963.94  (8.38) | 122021.39  (46.56) | 118087.97  (45.06) |
| Male | 803256.37 | 68508.19  (8.53) | 339874.88  (42.31) | 394873.31  (49.16) | 173739.81 | 14784.99  (8.51) | 73368.83  (42.23) | 85586.00  (49.26) |
| **IDII** | -2105763.19 | -93883.09  (4.46) | 8540305.24  (-405.57) | -10552185.33  (501.11) | 8765.21 | -5852.61  (-66.77) | 358709.88  (4092.43) | -344092.07  (-3925.66) |
| Female | -433131.68 | -45645.25  (10.54) | 4023000.80  (-928.82) | -4410487.23  (1018.28) | 30146.33 | -3226.67  (-10.70) | 168448.49  (558.77) | -135075.49  (-448.07) |
| Male | -1672631.51 | -48052.55  (2.87) | 4515662.16  (-269.97) | -6140241.12  (367.10) | -21381.13 | -2559.22  (11.97) | 190236.95  (-889.74) | -209058.86  (977.77) |
| **Schizophrenia** | 165929.07 | 32947.02  (19.86) | 166957.91  (100.62) | -33975.85  (-20.48) | 189625.59 | 37680.54  (19.87) | 199677.62  (105.30) | -47732.56  (-25.17) |
| Female | 119853.22 | 23809.44  (19.87) | 132419.19  (110.48) | -36375.41  (-30.35) | 77845.85 | 15758.25  (20.24) | 87557.32  (112.48) | -25469.72  (-32.72) |
| Male | 165929.07 | 32947.02  (19.86) | 166957.91  (100.62) | -33975.85  (-20.48) | 111779.74 | 22237.83  (19.89) | 112597.48  (100.73) | -23055.57  (-20.63) |

ADHD, attention-deficit hyperactivity disorder; ASD, autism spectrum disorders; IDII, idiopathic developmental intellectual disability.

**Supplementary Table 6.** The correlation between prevalence and DALY of mental disorders and SDI in 21 GBD regions and 204 countries and territories.

| **Cause** | **21 region** | | | | **204 countries and territories** | | | |
| --- | --- | --- | --- | --- | --- | --- | --- | --- |
|  | Prevalence | | DALYs | | Prevalence | | DALYs | |
|  | *r* | *p* | *r* | *p* | *r* | *p* | *r* | *p* |
| Mental disorders | 0.3409 | <0.0001 | 0.3428 | <0.0001 | 0.4986 | <0.001 | 0.4871 | <0.0001 |
| Anxiety disorders | 0.2908 | <0.0001 | 0.3066 | <0.0001 | 0.5395 | <0.001 | 0.5480 | <0.0001 |
| Attention-deficit/hyperactivity disorder | 0.5349 | <0.0001 | 0.5356 | <0.0001 | 0.5534 | <0.001 | 0.5581 | <0.0001 |
| Autism spectrum disorders | 0.4804 | <0.0001 | 0.5009 | <0.0001 | 0.2275 | 0.0011 | 0.2443 | 0.0004 |
| Bipolar disorder | 0.2935 | <0.0001 | 0.3161 | <0.0001 | 0.3185 | <0.001 | 0.3310 | <0.0001 |
| Conduct disorder | 0.4296 | <0.0001 | 0.4673 | <0.0001 | 0.3504 | <0.001 | 0.3785 | <0.0001 |
| Depressive disorders | 0.0485 | 0.1985 | 0.0663 | 0.0786 | 0.1701 | 0.0151 | 0.2052 | 0.0033 |
| Eating disorders | 0.7343 | <0.0001 | 0.7415 | <0.0001 | 0.8167 | <0.001 | 0.8222 | <0.0001 |
| Idiopathic developmental intellectual disability | -0.6047 | <0.0001 | -0.5445 | <0.0001 | -0.6422 | <0.001 | -0.6094 | <0.0001 |
| Schizophrenia | 0.1104 | 0.0034 | 0.1344 | 0.0004 | 0.1430 | 0.0414 | 0.1507 | 0.0316 |

**Supplementary Table 7.** The results of regression and concentration curves analysis for mental disorders and 9 subtypes.

| **Causes** | **Slope index of inequality** (95% CI) | | | | **Concentration index** (95% CI) | | | |
| --- | --- | --- | --- | --- | --- | --- | --- | --- |
|  | **ASPR** | | **ASDR** | | **ASPR** | | **ASDR** | |
|  | 1990 | 2021 | 1990 | 2021 | 1990 | 2021 | 1990 | 2021 |
| **Mental disorders** | 2165.62(1401.89, 2929.35) | 3268.15(2407.31, 4128.99) | 247.69(146.31, 349.06) | 440.23(322.63, 557.82) | 0.04(0.03, 0.06) | 0.06(0.04, 0.07) | 0.05(0.03, 0.07) | 0.07(0.05, 0.08) |
| Female | 2132.68(1297.16, 2968.21) | 3619.34(2595.29, 4643.40) | 333.29(203.28, 463.31) | 580.79(427.94, 733.63) | 0.05(0.03, 0.06) | 0.07(0.05, 0.08) | 0.06(0.04, 0.08) | 0.08(0.06, 0.10) |
| Male | 2219.71(1452.60, 2986.82) | 2982.65(2158.25, 3807.06) | 169.73(93.97, 245.48) | 306.19(222.21, 390.17) | 0.04(0.03, 0.05) | 0.05(0.04, 0.06) | 0.04(0.02, 0.05) | 0.05(0.04, 0.06) |
| **Anxiety disorders** | 1002.16(633.54, 1370.77) | 1770.75(1319.31, 2222.19) | 128.08(82.58, 173.59) | 220.29(165.02, 275.57) | 0.08(0.05, 0.10) | 0.10(0.07, 0.12) | 0.08(0.06, 0.11) | 0.10(0.07, 0.12) |
| Female | 1510.60(1040.22, 1980.97) | 2499.32(1923.82, 3074.82) | 190.36(133.44, 247.29) | 308.18(238.81, 377.55) | 0.10(0.07, 0.12) | 0.11(0.09, 0.13) | 0.10(0.07, 0.12) | 0.11(0.09, 0.13) |
| Male | 509.87(237.38, 782.37) | 1098.40(757.82, 1438.98) | 67.06(33.35, 100.78) | 137.77(95.76, 179.78) | 0.06(0.04, 0.08) | 0.08(0.06, 0.10) | 0.06(0.04, 0.08) | 0.08(0.06, 0.10) |
| **ADHD** | 1369.66(1120.41, 1618.91) | 1251.37(988.28, 1514.45) | 16.90(13.86, 19.94) | 15.38(12.17, 18.58) | 0.14(0.11, 0.18) | 0.14(0.10, 0.17) | 0.14(0.11, 0.18) | 0.14(0.10, 0.17) |
| Female | 741.90(612.04, 871.75) | 667.32(525.80, 808.83) | 9.11(7.55, 10.68) | 8.17(6.44, 9.89) | 0.13(0.09, 0.17) | 0.12(0.08, 0.16) | 0.13(0.09, 0.17) | 0.12(0.08, 0.16) |
| Male | 1925.63(1562.62, 2288.63) | 1781.47(1393.62, 2169.33) | 23.77(19.34, 28.19) | 21.95(17.19, 26.72) | 0.15(0.11, 0.18) | 0.14(0.10, 0.18) | 0.15(0.11, 0.18) | 0.14(0.10, 0.18) |
| **ASP** | 59.12(3.17, 115.07) | 80.78(25.59, 135.97) | 14.34(3.72, 24.95) | 16.98(6.36, 27.59) | 0.03(0.01, 0.04) | 0.03(0.01, 0.04) | 0.03(0.02, 0.04) | 0.03(0.01, 0.04) |
| Female | -43.69(-85.32, -2.05) | -35.20(-77.09, 6.69) | -6.30(-14.23, 1.64) | -5.75(-13.75, 2.24) | -0.01(-0.02, 0.01) | -0.01(-0.02, 0.01) | -0.00(-0.02, 0.01) | -0.00(-0.02, 0.01) |
| Male | 136.97(61.62, 212.31) | 170.20(94.50, 245.89) | 30.31(15.95, 44.67) | 34.56(20.07, 49.06) | 0.04(0.03, 0.05) | 0.04(0.03, 0.06) | 0.04(0.03, 0.06) | 0.04(0.03, 0.06) |
| **Bipolar disorder** | 139.21(83.75, 194.67) | 147.19(93.23, 201.16) | 31.66(19.39, 43.93) | 32.79(20.88, 44.71) | 0.10(0.06, 0.13) | 0.10(0.07, 0.13) | 0.10(0.06, 0.13) | 0.10(0.07, 0.13) |
| Female | 161.59(98.93, 224.25) | 169.85(107.78, 231.92) | 36.42(22.66, 50.18) | 37.52(23.93, 51.11) | 0.10(0.07, 0.13) | 0.11(0.07, 0.14) | 0.10(0.07, 0.13) | 0.11(0.07, 0.14) |
| Male | 118.22(69.05, 167.38) | 124.96(77.36, 172.57) | 27.10(16.17, 38.04) | 28.16(17.58, 38.74) | 0.09(0.06, 0.12) | 0.10(0.06, 0.12) | 0.09(0.06, 0.13) | 0.10(0.07, 0.12) |
| **Conduct disorder** | 95.09(56.83, 133.35) | 91.70(52.43, 130.97) | 13.84(9.23, 18.46) | 12.59(7.79, 17.40) | 0.03(0.02, 0.03) | 0.02(0.02, 0.03) | 0.03(0.02, 0.04) | 0.02(0.02, 0.03) |
| Female | 102.85(53.22, 152.48) | 94.65(46.51, 142.79) | 14.14(8.20, 20.09) | 12.52(6.62, 18.41) | 0.03(0.02, 0.04) | 0.02(0.01, 0.03) | 0.03(0.02, 0.04) | 0.02(0.01, 0.03) |
| Male | 75.23(47.03, 103.42) | 84.30(53.76, 114.85) | 12.23(8.84, 15.63) | 11.50(8.01, 14.98) | 0.02(0.02, 0.03) | 0.02(0.02, 0.03) | 0.03(0.02, 0.03) | 0.02(0.02, 0.03) |
| **Depressive disorders** | -54.49(-263.51, 154.54) | 314.25(64.92, 563.58) | 3.11(-38.10, 44.32) | 75.11(25.42, 124.80) | 0.01(-0.02, 0.04) | 0.04(0.02, 0.07) | 0.02(-0.01, 0.05) | 0.05(0.02, 0.08) |
| Female | 38.88(-242.61, 320.38) | 516.78(179.46, 854.09) | 27.49(-27.83, 82.82) | 122.60(55.37, 189.83) | 0.03(-0.00, 0.06) | 0.06(0.03, 0.09) | 0.04(0.01, 0.07) | 0.07(0.04, 0.10) |
| Male | -88.87(-237.84, 60.09) | 160.74(-8.50, 329.98) | -11.46(-40.74, 17.81) | 37.31(1.94, 72.69) | -0.00(-0.03, 0.02) | 0.02(-0.00, 0.05) | -0.00(-0.03, 0.03) | 0.03(-0.00, 0.05) |
| **Eating disorders** | 199.15(162.89, 235.40) | 257.95(218.35, 297.55) | 42.84(35.23, 50.44) | 55.47(47.12, 63.82) | 0.25(0.21, 0.27) | 0.26(0.22, 0.28) | 0.25(0.22, 0.27) | 0.26(0.23, 0.28) |
| Female | 300.64(246.48, 354.81) | 383.00(322.29, 443.71) | 65.00(53.40, 76.61) | 82.68(69.67, 95.69) | 0.29(0.25, 0.32) | 0.29(0.26, 0.32) | 0.29(0.25, 0.32) | 0.29(0.26, 0.32) |
| Male | 97.38(81.40, 113.36) | 132.23(116.39, 148.07) | 21.36(17.93, 24.78) | 28.64(25.24, 32.05) | 0.17(0.15, 0.19) | 0.19(0.17, 0.21) | 0.17(0.15, 0.19) | 0.19(0.17, 0.21) |
| **IDII** | -600.24(-752.76, -447.71) | -556.61(-669.55, -443.68) | -22.43(-28.31, -16.56) | -21.22(-25.68, -16.75) | -0.23(-0.28, -0.18) | -0.23(-0.28, -0.18) | -0.21(-0.26, -0.17) | -0.21(-0.27, -0.16) |
| Female | -550.39(-693.26, -407.53) | -519.50(-633.14, -405.86) | -20.00(-25.66, -14.34) | -18.58(-23.05, -14.10) | -0.23(-0.27, -0.18) | -0.22(-0.27, -0.17) | -0.21(-0.26, -0.16) | -0.20(-0.25, -0.15) |
| Male | -635.49(-788.62, -482.36) | -576.91(-689.76, -464.06) | -24.32(-30.49, -18.15) | -23.17(-27.99, -18.35) | -0.23(-0.28, -0.18) | -0.24(-0.29, -0.18) | -0.22(-0.27, -0.17) | -0.22(-0.28, -0.17) |
| **Schizophrenia** | 2.35(-1.22, 5.92) | 3.01(-0.42, 6.43) | 2.08(-0.34, 4.50) | 2.15(-0.16, 4.46) | 0.02(0.00, 0.03) | 0.02(0.00, 0.03) | 0.02(0.00, 0.03) | 0.02(0.00, 0.03) |
| Female | -2.95(-5.80, -0.10) | -2.05(-4.92, 0.83) | -1.57(-3.46, 0.32) | -1.32(-3.23, 0.60) | -0.00(-0.02, 0.01) | -0.00(-0.01, 0.01) | -0.00(-0.01, 0.01) | -0.00(-0.01, 0.01) |
| Male | 8.46(3.77, 13.15) | 8.31(3.70, 12.92) | 6.21(2.97, 9.45) | 5.64(2.55, 8.73) | 0.03(0.01, 0.06) | 0.04(0.02, 0.06) | 0.04(0.01, 0.06) | 0.04(0.02, 0.06) |

ASPR, the age-standardized prevalence rate; ASDR, the age-standardized DALYs rate; ADHD, attention-deficit hyperactivity disorder; ASD, autism spectrum disorders; IDII, idiopathic developmental intellectual disability.

**Supplementary Table 8.** Predictions of prevalence and DALYs for mental disorders from 2022 to 2050 at the global level.

|  | **Prevalence** | | **DALYs** | |
| --- | --- | --- | --- | --- |
|  | Number (95% CI) | ASPR (95% CI) | Number (95% CI) | ASDR (95% CI) |
| **Global** | 188946955.35(123066101.64,254827809.06) | 6120.71(3973.57,8267.85) | 26061361.15(16335728.5,35786993.72) | 844.71(529.48,1159.94) |
| **Sex** |  |  |  |  |
| Female | 81712441.33(50319599.59,113105283.06) | 5461.36(3363.18,7559.54) | 12371362.07(7302029.37,17440694.77) | 826.86(488.04,1165.67) |
| Male | 107619671.00(72194659.10,143044682.91) | 6772.50(4543.26,900.90) | 13614007.11(8941591.99,18286422.22) | 856.74(562.70,1150.78) |
| **Age** |  |  |  |  |
| <5 years | 8825960.00(2494911.72,15157008.29) | 1496.68(432.59,2560.76) | 893733.47(194598.81,1592868.12) | 151.56(33.00,270.12) |
| 5-9 years | 23113495.91(10219265.71,36007726.12) | 3815.97(1704.39,5927.55) | 2228249.62(863540.91,3592958.34) | 367.79(142.54,593.05) |
| 10-14 years | 45995423.58(26523292.79,65467554.36) | 7438.05(4311.37,10564.72) | 5659614.18(2996406.13,8322822.24) | 914.90(484.38,1345.43) |
| 15-19 years | 58727859.17(40203919.82,77251798.51) | 9331.14(6403.88,12258.40) | 8699235.05(5585404.35,11813065.76) | 1381.67(887.11,1876.23) |
| 20-24 years | 67723018.29(51526528.13,83919508.46) | 10560.41(8042.34,13078.48) | 11269190.42(8120407.36,14417973.49) | 1756.71(1265.86,2247.56) |
| **Anxiety disorders** | 148009866.43(68361256.14,227658476.73) | 4797.35(2215.75,7378.95) | 18300198.55(8513248.55,28087148.5) | 593.15(275.93,910.37) |
| Female | 90842020.84(41545342.53,140138699.14) | 6071.55(2776.74,9366.36) | 11190912.70(5156467.88,17225357.52) | 747.96(344.64,1151.28) |
| Male | 57031327.44(26578742.16,87483912.72) | 3589.02(1672.62,5505.42) | 7080273.34(3308056.87,10852489.81) | 445.57(208.18,682.95) |
| **ADHD** | 61583661.70(37969729.78,85197593.61) | 1996.07(1230.69,2761.46) | 766407.71(464651.98,1068163.43) | 24.84(15.06,34.62) |
| Female | 16971882.60(10555670.01,23388095.19) | 1134.34(705.50,1563.17) | 211835.72(124662.23,299009.21) | 14.16(8.33,19.98) |
| Male | 44715198.48(27515773.12,61914623.83) | 2813.96(1731.59,3896.33) | 556779.64(336163.90,777395.37) | 35.04(21.16,48.92) |
| **ASD** | 26721132.04(17896129.32,35546134.76) | 866.10(580.06,1152.13) | 5184379.48(3468258.45,6900500.52) | 168.04(112.41,223.66) |
| Female | 8617154.48(5602890.46,11631418.50) | 575.94(374.48,777.40) | 1664275.33(1076059.51,2252491.15) | 111.23(71.92,150.55) |
| Male | 18113739.94(12215745.87,24011734.01) | 1139.91(768.75,1511.07) | 3522664.79(2370082.95,4675246.63) | 221.68(149.15,294.22) |
| **Bipolar disorder** | 7508543.02(3615358.09,11401727.96) | 411.66(411.37,411.95) | 1662950.79(797797.19,2528104.38) | 88.00(42.22,133.78) |
| Female | 3800557.51(1830336.14,5770778.89) | 414.64(199.69,629.59) | 830390.61(396890.69,1263890.54) | 90.60(43.30,137.89) |
| Male | 3698021.69(1771509.98,5624533.41) | 396.10(395.70,396.50) | 830508.46(394035.82,1266981.11) | 85.35(40.49,130.20) |
| **Conduct disorder** | 39224861.78(21805145.65,56644577.90) | 1571.79(873.76,2269.82) | 4824440.08(2671687.71,6977192.45) | 193.32(107.06,279.58) |
| Female | 13654510.55(7347890.13,19961130.98) | 1128.19(607.11,1649.27) | 1666163.19(882703.15,2449623.22) | 137.66(72.93,202.40) |
| Male | 25690181.15(14425194.86,36955167.44) | 1998.84(1122.36,2875.32) | 3170836.14(1768552.40,4573119.88) | 246.71(137.60,355.81) |
| **Depressive disorders** | 79354180.88(35941824.04,122766537.72) | 2572.06(1164.96,3979.15) | 15767599.01(6008139.24,25527058.77) | 511.07(194.74,827.39) |
| Female | 47888460.30(21291631.46,74485289.14) | 3200.69(1423.06,4978.33) | 9483537.92(3538315.85,15428759.99) | 633.85(236.49,1031.20) |
| Male | 31394932.62(14460080.01,48329785.23) | 1975.70(909.98,3041.43) | 6178001.28(2367106.38,9988896.19) | 388.79(148.96,628.61) |
| **Eating disorders** | 6389901.43(4267442.57,8512360.29) | 256.05(171.00,341.10) | 1364896.45(897112.92,1832679.97) | 54.69(35.95,73.44) |
| Female | 4132480.41(2729426.70,5535534.12) | 341.44(225.52,457.37) | 879689.63(567350.85,1192028.41) | 72.68(46.88,98.49) |
| Male | 2220307.93(1463475.85,2977140.02) | 172.75(113.87,231.64) | 475252.01(305516.30,644987.71) | 36.98(23.77,50.18) |
| **IDII** | 35096787.74(18513788.93,51679786.56) | 1137.57(600.08,1675.06) | 1473003.15(774566.63,2171439.67) | 47.74(21.10,70.38) |
| Female | 17352031.84(8905867.11,25798196.57) | 1159.75(595.24,1724.26) | 720878.10(365507.9,1076248.29) | 48.18(24.43,71.93) |
| Male | 17671339.00(9515191.25,25827486.75) | 1112.07(598.80,1625.34) | 749076.60(400979.93,1097173.27) | 47.14(25.23,69.05) |
| **Schizophrenia** | 2136249.62(1235473.02,3037026.22) | 113.05(65.38,160.71) | 1431660.15(820959.89,2042360.40) | 7.76(43.44,108.08) |
| Female | 990093.18(556870.07,1423316.30) | 108.02(60.75,155.28) | 658003.95(363916.80,952091.09) | 71.79(39.70,103.87) |
| Male | 1150764.40(666911.09,1634617.71) | 118.26(68.53,167.98) | 776474.01(446186.66,1106761.36) | 79.79(45.85,113.73) |

ASPR, the age-standardized prevalence rate; ASDR, the age-standardized DALYs rate; ADHD, attention-deficit hyperactivity disorder; ASD, autism spectrum disorders; IDII, idiopathic developmental intellectual disability.
